# Selection from a pool of self-assembling lipid replicators

**DOI:** 10.1038/s41467-019-13903-x

**Published:** 2020-01-10

**Authors:** Ignacio Colomer, Arseni Borissov, Stephen P. Fletcher

**Affiliations:** Department of Chemistry, Chemistry Research Laboratory, University of Oxford, Mansfield Road, Oxford, OX1 3TA United Kingdom

**Keywords:** Origin of life, Self-assembly

## Abstract

Replication and compartmentalization are fundamental to living systems and may have played important roles in life’s origins. Selection in compartmentalized autocatalytic systems might provide a way for evolution to occur and for life to arise from non-living systems. Herein we report selection in a system of self-reproducing lipids where a predominant species can emerge from a pool of competitors. The lipid replicators are metastable and their out-of-equilibrium population can be sustained by feeding the system with starting materials. Phase separation is crucial for selective surfactant formation as well as autocatalytic kinetics; indeed, no selection is observed when all reacting species are dissolved in the same phase. Selectivity is attributed to a kinetically controlled process where the rate of monomer formation determines which replicator building blocks are the fittest. This work reveals how kinetics of a phase-separated autocatalytic reaction may be used to control the population of out-of-equilibrium replicators in time.

## Abstract (web summary)

Selection in compartmentalized self-replicating systems might provide a way for life to arise from abiotic environments. Here, the authors explore selection in a system of transient autocatalytic lipids and find that autocatalytic kinetics and phase separation are the key selection factors.

## Introduction

Living systems are comprised of various functional assemblies and machines which control molecular and greater length-scale processes. These systems operate far-from-equilibrium to control self-organization and synthetic processes including self-replication^1–4^. Encoding, transmitting and reading information are widely understood to be important in self-replication, along with the presence of a metabolism and the ability of a system to undergo evolution.

**Fig. 1:**
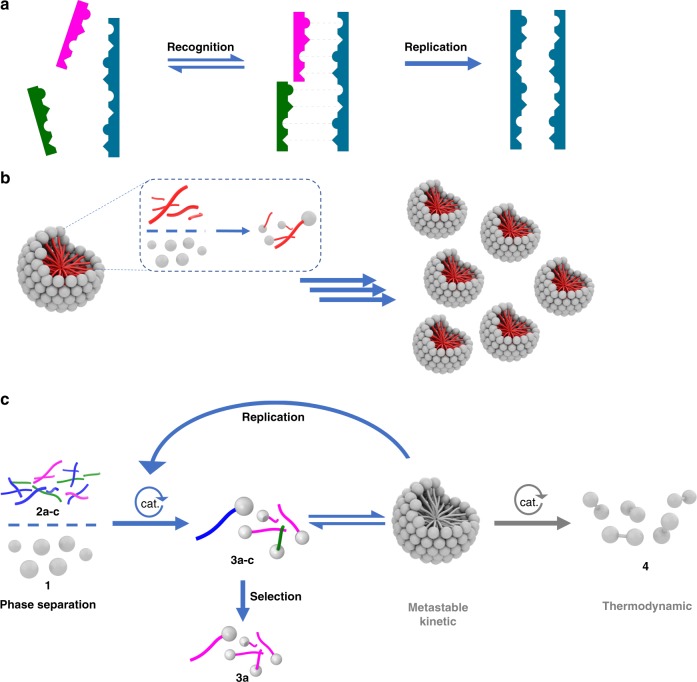
Selectivity in autocatalytic species. **a** Template replicators operate by a recognition mechanism, directing their own formation via template-directed processes. Synthetic template replicators are often inspired by biological replicators such as DNA and RNA. **b** Surfactant replicators operate by self-assembly of lipid aggregates. Aggregates, such as micelles and vesicles promote reactions between phase-separated components to form more lipid building blocks and additional self-assembled aggregates. **c** This work: selectivity in a pool of competing replicators. Reaction between hydrophilic **1** and hydrophobic **2** leads to amphiphilic metastable building-blocks **3**, which self-assemble and are consumed to form thermodynamic waste product **4**. Selection from a pool of competitive reactants **2a–c** is observed to preferentially form metastable building block **3a** during replication under out-of-equilibrium conditions. Selection is attributed to kinetically controlled preferential formation of the fastest replicator.

Self-replication has been described in a variety of synthetic systems^5,6^. The most widely studied class of replicators are based on templated autocatalysis where interactions between self-replicating product and precursors form additional product (Fig. 1a). This molecular replication mechanism is well-known in living systems where RNA and DNA store, transmit and duplicate information. The “RNA world” is based on the idea that chemical processes generated autocatalytic oligomers of RNA, which could then undergo selection and evolution.

Cellular compartments concentrate and isolate the machinery of life, including informational template molecules like RNA, from the environment^7^. Another prebiotic hypothesis involves the “lipid world”, which postulates that autocatalytic amphiphilic boundary structures preceded biopolymers and could have provided suitable microenvironments for the emergence of cellular life^8^.

Self-replicating surfactants are therefore intriguing species that operate by different autocatalytic mechanisms than template replicators (Fig. 1). Lipid-based systems self-replicate by forming supramolecular aggregates, which catalyze interfacial reactions to form monomeric lipids, the building blocks of the aggregates^9,10^. The formation of compartments by product self-assembly is inherent in these systems. Compartmentalization localizes and encloses chemical components and reactions, and drives replication (Fig. 1b).

One critical difference between autocatalysts that self-reproduce by a template-directed mechanism and those that replicate using lipid-aggregate mechanisms is in the emergent properties that have been demonstrated in each case. For example, template replicators have been shown to be capable of complex behavior such as selection, where a predominant species can emerge from competing components^5,11–13^. Such experiments are generally viewed as being supportive of prebiotic ‘replicator-first scenarios’ such as the RNA world. In contrast, examples of self-replicator selection in systems of lipid aggregates which spontaneously form cell-like structures are unknown^14^.

A number of theoretical studies have used non-equilibrium models to provide insight into how selective autocatalysts may emerge, grow and replicate^15,16^. The graded autocatalysis replication domain (GARD) model has been used to study how compositional genomes may appear and evolve in a lipid world scenario^17–19^. Intriguingly, this model predicts that selection will not be observed in organized, mutually catalytic networks at equilibrium, but will be enabled when replication occurs out-of-equilibrium, and that at a threshold called the Morowitz boundary such a system may lead to natural selection^14^.

Synthetic replicators generally form kinetically trapped or thermodynamic products^20–26^ until their precursors are consumed, at which point the system moves toward thermodynamic equilibrium. We recently reported a metastable autocatalytic lipid that is both created and destroyed, so that the replicator is a kinetic rather than thermodynamic product^27^. These lipids are produced by a catalyst-mediated interfacial reaction from two phase-separated species, followed by self-organization into micelles. The lipid subsequently undergoes a catalyst-mediated destruction. Transiently formed self-aggregating lipids represent a unique opportunity to explore replication under out-of-equilibrium conditions. Synthetic systems that mimic the non-equilibrium dynamic functions seen in biology may be capable of performing work^28–31^. Supramolecular structures that can maintain dynamic steady states should make it possible to observe functions that cannot be observed at equilibrium ^32–37^.

Herein we report selection in a system of self-reproducing lipids that form micelles and mimic primitive cell-like aggregates. Our system consists of phase-separated substrates: hydrophilic alkene **1** and hydrophobic alkenes **2a**–**c** (Fig. 1c) that react across the interface to produce a set of metastable building blocks **3a**–**c** which are then consumed to form the thermodynamic product **4**.

When all reacting species are dissolved in the same phase, **3a**–**c** form as thermodynamic products and no selection in their formation is observed. However, selection—where one lipid replicator preferentially forms over another—does occur under phase-separated conditions where **3a**–**c** form transiently as kinetic products. Selection in building blocks **3a**–**c** produces a lipid population enriched in certain replicators, which can be sustained in time via continuous influx of reagents in an open system (continuously stirred tank reactor). Mechanistic analysis of this system suggests that selection is based on kinetic amplification of the replicator that is formed fastest.

## Results

### Phase separation triggers replicator selection

Using the recently reported metastable surfactant replicators **3a** or **3c**^27^ as a platform, we decided to explore what differences may be observed between self-replicators kept out-of-equilibrium and (already well-explored) self-replication to a thermodynamically stable product^5,6^.

**Fig. 2:**
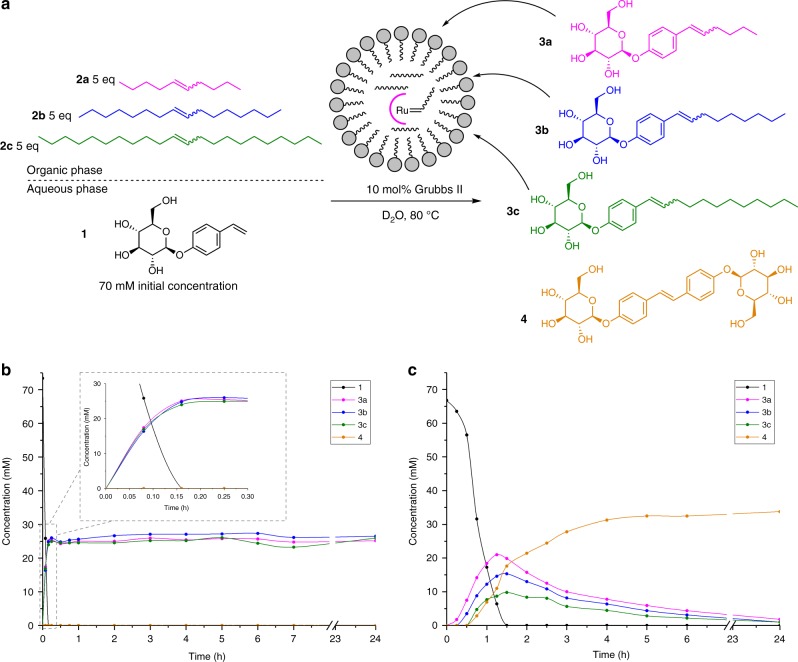
Formation of transient surfactants under homogeneous and phase-separated conditions. Concentration vs. time of hydrophilic **1** (black), amphiphiles **3a** (magenta), **3b** (blue), **3c** (green) and waste product **4** (yellow), determined via UPLC separation of an aliquot of the reaction mixture. **a** Reaction scheme and conditions. **b** Under homogeneous conditions in *t*-BuOH:D_2_O, no autocatalytic kinetics were observed and formation of **3a–c** was non-selective. **c** Reaction under biphasic conditions, using D_2_O and a 1:1:1 mixture of **2a:2b:2c**. Autocatalytic kinetics in the consumption of **1** and selectivity in lipid formation are observed. Source data are provided as a Source Data file.

Hydrophobic alkenes **2a**–**c** differ only in their carbon chain lengths by three methylene unit increments. Using Grubbs 2nd generation catalyst, we first studied alkene metathesis between **1** and **2a**–**c** without phase separation, using a *t*-BuOH:D_2_O solvent mixture where all reaction components are soluble. The hydrophobic reagents **2a**–**c** were used in excess (5 eq) to minimize the effect of their consumption on reaction rates. These single-phase reactions gave amphiphiles **3a**, **3b** and **3c**. Here, the kinetics of amphiphile formation were linear and no lag period or autocatalytic kinetics were seen (Fig. 2a). No selection was observed between **3a**–**c** which formed in an ~1:1:1 ratio. We also note that under these conditions, amphiphiles **3a**–**c** appear thermodynamically stable and very little, if any, **4** was formed.

Using D_2_O as solvent so that hydrophilic **1** and excess hydrophobic **2a–c** are phase separated, the concentration profiles of building blocks **3a–c** follow the expected pattern for the replication of a metastable species. The exact profiles vary by building block, but each features an initial lag period, followed by exponential increase and finally consumption after reaching a concentration maximum (Fig. 2b). Selection, in the form of preferential formation of one species over another, for the formation of **3a** over **3b** and **3c** is clearly observed. This is most notable at the beginning of the reaction when only **3a** is formed in detectable amounts. Formation of **3a** is followed by the appearance of **3b** and then **3c**. There is a direct correlation between the length of the initial lag period and the length of the hydrophobic chain. This trend between the reaction rate and chain length is maintained until the limiting reagent **1** is fully consumed. These results are related to the known effects of phase separation on the composition of dynamic covalent libraries^38–40^, albeit in this case selectivity is observed in transient kinetic rather than thermodynamic products.

In this system, both autocatalysis and cross-catalysis are readily demonstrated by seeding the reaction of **1** and **2c** to produce **3c** with **3c** (Supplementary Fig. 29) or with **3a** (Supplementary Fig. 30). In these experiments, the lag periods are no longer present and the formation of **3c** is accelerated. On this basis, surfactants **3a–c** are all expected to act as autocatalysts and cross-catalysts, although no other pathways were explicitly visualized. Cross-catalysis where **3a** promotes formation of **3c** is slightly less effective than autocatalysis with **3c**, presumably due to the much higher critical micelle concentration (CMC) of **3a** (CMC’s at 60 °C, **3a**: 0.68 mM; **3b**: 0.09 mM; **3c**: 0.004 mM, see Supplementary Figs. 39–41).

Surfactants **3a–c** dispersed in aqueous solutions at low concentrations (<0.3 mM for **3b** and **3c**) formed spherical micelles close to 30 nm in diameter. For **3a**, these assemblies persisted at higher concentrations, while in the case of **3b** and **3c** granular aggregates were observed by transmission electron microscopy (TEM) and particle sizes of 100–600 nm were detected by dynamic light scattering (DLS).

### CSTR allows sustained populations of metastable replicators

**Fig. 3:**
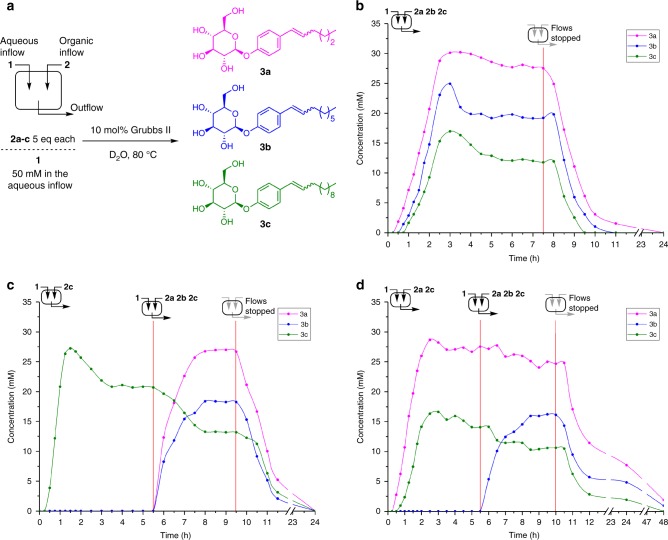
Competition experiments between different replicator building blocks using a CSTR. Concentration vs. time is shown for **3a** (magenta), **3b** (blue) and **3c** (green). **a** Scheme of the CSTR setup and the competing transient replicators. **b** Using a 1:1:1 mixture of **2a**, **2b** and **2c**, different steady-state concentrations of amphiphiles are established after transient peaks allowed by initial reaction conditions. **c** Starting with only **2c** establishes a steady state of **3c**; changing to a 1:1:1 mixture of **2a**:**2b**:**2c**, moves the system to a similar state as above. **d** Initially using a 1:1 mixture of alkenes **2a**:**2c** leads to a steady state of both **3a** and **3c**. Switching to a 1:1:1 mixture of **2a**:**2b**:**2c** again forms a distribution of **3a–c**. Source data are provided as a Source Data file.

A steady-state replicator population can be achieved under open-system conditions using a continuous stirred tank reactor (CSTR), with a balanced influx of starting material and efflux of the reaction mixture (Fig. 3)^41,42^. The system is thus kept in a non-equilibrium dynamic steady state when a single building block is involved (see Supplementary Figs. 32, 33). With the system operating in a CSTR using a 1:1:1 mixture of alkenes **2a**, **2b** and **2c**, an initial lag period was observed, followed by exponential growth of the building blocks **3a**, **3b** and **3c** (Fig. 3a). A dynamic steady state dependent on reagent influx rate is then achieved after a transient concentration maximum resulting from consumption of **1** present in the initial reaction mixture. The three amphiphiles reached different steady-state concentrations; the one with the highest population was **3a**, followed by **3b** and **3c**. This is the same selectivity trend as observed under batch conditions (Fig. 2b).

The difference in the populations of **3a**–**c** is maintained for as long as the CSTR operates, or for as long as energy in the form of chemical reactants is provided, allowing the consumption of **1** and **2a**–**c** and their transformation into replicators **3a**–**c**. After 7.5 h, turning off the flows results in the system moving toward equilibrium, with complete consumption of the remaining **1** and **3a**–**c** to form **4** (Fig. 3a).

To see how the populations of competing building blocks adapt to changes in the composition of hydrophobic phase, we designed an experiment to establish a steady state of the least fit building block **3c** starting only with **2c** (Fig. 3b). After 5.5 h, the hydrophobic channel was switched to a 1:1:1 mixture of alkenes **2a**:**2b**:**2c**. Surfactants **3a** and **3b** started to form without a lag period, due to the amphiphile **3c** being already present. While the concentrations of **3a** and **3b** increased, the concentration of the originally present **3c** and **1** decreased, leading to a new steady state which is essentially the same as shown in Fig. 3a. The system readily adapts to the new conditions where the relative concentrations are established by balancing the kinetics of formation/destruction and the flow parameters. Again, when the flows were turned off (after 9.5 h), the system moved toward thermodynamic equilibrium (Fig. 3b).

Intrigued by this robust display of adaptability, we conducted an experiment where a steady state of two amphiphiles “fittest” **3a** and “least fit” **3c** was first obtained (Fig. 3c). Using a 1:1 mixture of **2a**:**2c**, the population of **3a** was approximately double that of **3c**. After 5.5 h, switching to a 1:1:1 mixture of **2a**:**2b**:**2c** caused the system to respond by slightly decreasing the concentrations of **3a** and **3c**, and concomitant formation of **3b**. The population of **3b** eventually outcompeted **3c** and the system reached the same steady state as above, independent of the initial conditions. Turning off the flows once again led to the equilibrium state, with complete conversion of the remaining amphiphiles into **4** (Fig. 3c).

### Kinetically controlled selectivity

**Fig. 4:**
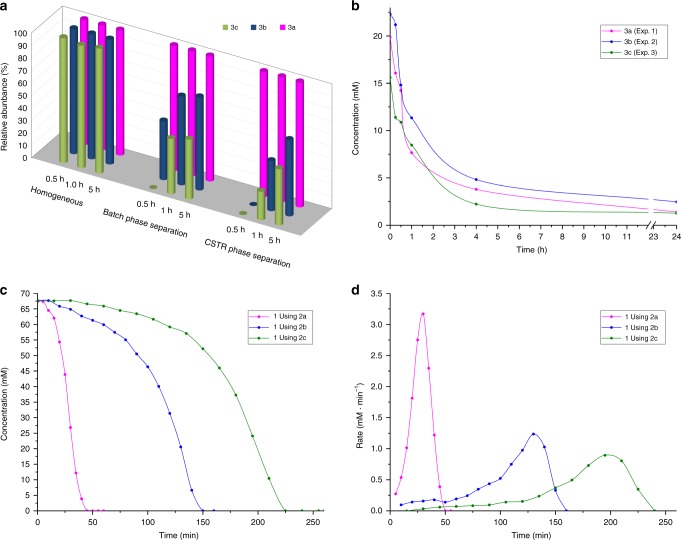
Selection of replicators arises from phase separation and their different kinetic properties. **a** Relative abundance of building blocks **3a** (magenta), **3b** (blue) and **3c** (green) at different reaction times under homogeneous and biphasic (batch and CSTR) conditions. **b** No significant difference in destruction rates of **3a**–**c** was observed. **c** Concentration vs. time of **1** using **2a**, **2b** or **2c** for phase-separated batch conditions. **d** Rate of consumption of **1** vs. time using **2a**, **2b** or **2c** under biphasic batch conditions, showing the expected bell-shaped curves of autocatalytic reactions. Source data are provided as a Source Data file.

The data presented are summarized in a graph comparing phase-separated (under batch conditions and using CSTR) and homogeneous (without phase separation) experiments, at different reaction times (Fig. 4a). The main conclusions that can be extracted are: (1) Phase separation, which is crucial for keeping the system out-of-equilibrium, is also essential for selection. (2) Kinetic factors and selection are highly correlated, so that there is a strong time dependence on selectivity—for example, in batch reactions higher selectivity is observed at shorter reaction times.

To investigate the origin of selection in building block formation, we designed a series of experiments discussed below, which led us to the conclusion that this process is kinetically controlled. Destruction of the metastable building blocks was studied by independently subjecting **3a**, **3b** or **3c** to biphasic reaction conditions involving D_2_O, Grubbs 2nd generation catalyst and a 1:1:1 mixture of **2a**:**2b**:**2c**. Interestingly, every amphiphile was first converted into a mixture of **3a**, **3b** and **3c** (see Supplementary Fig. 34), consistent with a destruction mechanism where Ru-activated hydrophobic alkenes react with **3a**–**c**^27^. Finally, building blocks **3a**–**c** were consumed to form thermodynamically stable **4**. There is little, if any, significant difference in the rates of destruction of the different building blocks, especially at the early stages (Fig. 4b, consumption of **3a**–**c** from three independent experiments). As the destruction is non-selective, it follows that different rates of formation of **3a**–**c** must be responsible for selection.

Kinetic parameters of amphiphile formation were investigated to explain the observed selection. Carefully comparing the consumption of **1** with **2a** or **2b** or **2c** under batch reaction conditions shows sigmoidal kinetic profiles and reveals that the reaction is fastest with **2a** (Fig. 4c). Plotting the rate of consumption of **1** over time for each case (Fig. 4d and Supplementary Figs. 35–37) shows the expected bell-shaped profile for an autocatalytic reaction^11^, with an acceleration and a decay period. The maximum rate is almost three times faster for **3a** than for **3b** or **3c** (Fig. 4d, magenta line vs. blue or green).

While many processes are undoubtedly involved here^5^, insight may be gained by considering a highly simplified scenario. Assuming pseudo-first-order conditions, the process can be deconvoluted into two mechanisms: an uncatalyzed contribution responsible for initial building block formation (*v* = *k*_uncat_ · [**1**]), and a catalytic contribution that uses product to accelerate the reaction (*v* = *k*_cat_ · [**1**] · [**3**]), which allows an estimation of both rate constants^43^. While for **3b** and **3c** all rate constants are within one order of magnitude, the rate constants for **3a** are an order of magnitude higher (see Supplementary Figs. 35–38).

We propose that the rate of both catalyzed and uncatalyzed surfactant formation is dependent on the hydrophobicity of the alkyl chains being incorporated into the surfactant molecule. Indeed, shorter alkanes are known to have higher solubility in water^44^, in correlation with the observed kinetic selection where both uncatalyzed and catalyzed rate constants are in the order **3a**>**3b**>**3c**. However, water solubility of **2a**–**c** cannot by itself explain selection, as **3a**–**c** are formed via organometallic complexes where the respective alkyl chain is bound to Grubbs II (activated fatty ruthenocarbenes). Water solubility of these species is expected to be extremely low regardless of the length of Ru-bound alkyl chain, thus necessitating an interfacial reaction. We therefore propose that these Ru carbene intermediates have an increasing tendency to approach the aqueous-organic interface in the order of decreasing hydrophobicity, which translates to higher rates of surfactant synthesis.

As dispersions of pure **3a** tend to form smaller particles than **3b** and **3c** (Supplementary Figs. 43–50), it is also necessary to consider a potential influence of particle sizes on selectivity. For example, micelles made of **3a** may be more catalytically active due to their smaller size and therefore higher surface to volume ratio. Such an effect would require self-segregation of the surfactant mixture into small **3a**-rich micelles and larger **3b**/**3c**-rich assemblies. Additionally, selective uptake of hydrophobic alkenes into micelles would be required. An equimolar mixture of **3a**–**c** was shown by DLS to form particles in 100–300 nm range with no evidence of such partitioning. Furthermore, influence of particle size on reaction rate cannot explain the observed differences in uncatalyzed rate constants which quantify the rate of surfactant formation in absence of micelles. This leads us to conclude that differences in particle size are a highly unlikely contribution to the observed selectivity.

**Fig. 5:**
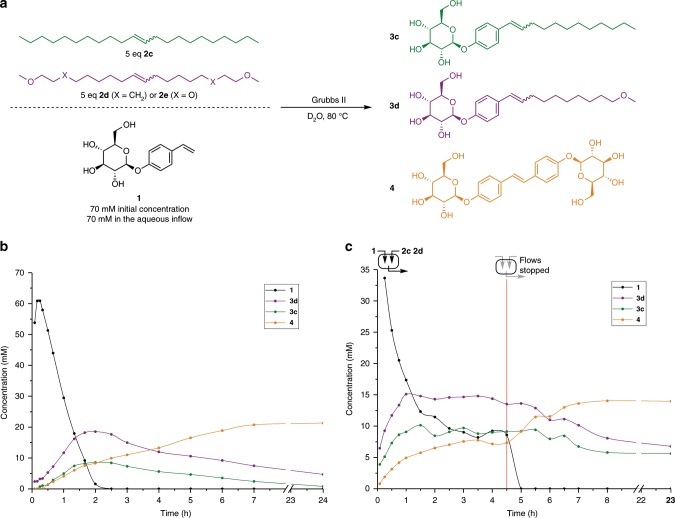
Selective formation of a surfactant with decreased hydrophobicity. Concentration vs. time is shown for **1** (black), **3c** (green), **3d** (purple) and **4** (orange). **a** Scheme showing the reaction and experimental conditions. **b** Experiment performed under biphasic batch conditions. **c** Experiment performed under CSTR conditions. Amphiphile **3d** is amplified relatively to **3c** in both cases. Source data are provided as a Source Data file.

To further test our hypothesis about the origin of selectivity, we conducted experiments using less hydrophobic ether-containing analogs of **2c**. Here, amphiphile **3d** was amplified relative to its pure alkyl chain counterpart **3c** under both batch (Fig. 5a) and CSTR (Fig. 5b) conditions using a 1:1 mixture of **2c** and **2d**. In both cases, concentrations of **3d** were ~2× higher than of **3c**, a similar strength of selection as observed for **3a** relative to **3c**. This demonstrates that very minor structural differences can lead to significant selectivity in amphiphile formation. When even more hydrophilic **2e** with four ether moieties was used in competition with **2c**, almost exclusive formation of the corresponding hydrophilic amphiphile occurred but was not followed by replicator destruction (see Supplementary Fig. 31). Additionally, no cross-catalytic behavior was observed while seeding the formation of **3c** with **2e**-derived amphiphile, implying that excessive hydrophilicity leads to a breakdown of self-assembly and disables the transient surfactant behavior.

## Discussion

In conclusion, we have experimentally demonstrated that selection can occur in a system of autocatalytic lipids that compete for a common precursor. Minor structural differences strongly affect which building blocks are selected for. Selection does not arise from formation of the thermodynamically most stable replicator, but rather from kinetic factors involved in the synthesis of building blocks. The system is highly dynamic in that it rapidly adapts and responds to changes in precursor concentration or composition. While the autocatalytic behavior is not the direct cause of selection, both of these phenomena are enabled by the same set of prerequisites. In particular, both require phase-separated reagents undergoing an interfacial reaction.

This work sheds light on unexplored aspects of autocatalysis and contributes to understanding of how replicator selection can occur in self-reproducing systems. While template-based replicators such as RNA or DNA have well understood mechanisms of selection and evolution, such mechanisms in other classes of replicators are scarcely studied. Here we reveal how kinetic effects may be used to exert selection and control the populations of dynamic species in time. This work also has implications for understanding how amphiphile-based prebiotic chemical systems can evolve in time.

## Methods

### General experimental details

Reagents were obtained from Sigma-Aldrich, Alfa, Fluorochem and TCI suppliers and used without further purification. Argon atmosphere and/or flame-dried glassware were used where anaerobic or anhydrous conditions were required. All reactions were stirred with magnetic followers. See Supplementary Methods for synthetic procedures and details of kinetic analysis. Flash column chromatography was performed using silica gel (60 Å, 0.033–0.070 mm, BDH). TLC analysis was done on Merck Kiesegel 60 F_254_ 0.25-mm precoated silica plates. Reactant flow in CSTR experiments was applied using World Precision Instruments AL-1000 syringe pumps.

### UPLC chromatography

Analytical separations were done using Waters Acquity Ultra Performance Liquid Chromatography (UPLC) H-Class with PDA detector and the data were analyzed with Empower software. Acquity UPLC BEH C18 column, 2.1 × 50 mm with a 1.7-µm-size particle was used, eluting with MeOH/H_2_O (5:95–95:5 over 5 min).

### CMC determination

Fluorimetry was done using Edinburgh Instruments Spectrofluorometer FS5 and the data collected with Fluoracle software. A literature method was followed^45^, using 1,6-diphenyl-1,3,5-hexatriene (DPH) as a fluorescent probe with excitation wavelength of 358 nm and emission wavelength of 430 nm. The CMC for alkenes 3a–d was extracted from a plot of emission vs. concentration (Supplementary Figs. 39–42). Experiments were performed at 60 °C to stay within optimal instrument parameters and the trends observed at 60 °C are expected to hold under reaction conditions at 80 °C.

### Dynamic light scattering

DLS analysis was done using a Malvern Zetasizer Nano ZEN5600 with Zetasizer software. For each measurement, 1.0 ml of sample solution was placed in a disposable plastic cuvette and thermostatted at 60 °C. See Supplementary Figs. 43–47 for DLS results.

### TEM experiment

Analysis was done using a FEI Tecnai 12 TEM at 120 kV with a Gatan OneView CMOS camera (Supplementary Figs. 48–50). Images were produced using negative staining. Freshly glow discharged carbon Formvar 200 mesh copper grids were treated with 10 µl of sample solution for 2 min, blotted with filter paper and stained with 2% uranyl acetate for 10 s, then blotted and air dried.

### Compounds characterization

^1^H NMR and ^13^C NMR spectra were recorded on Bruker AVIII HD Nanobay 400 MHz or Bruker AVIII HD 500 MHz spectrometers and referenced to residual solvent signals (Supplementary Figs. 1–11). High-resolution mass spectra were recorded on a Bruker MicroTOF under electrospray ionization, or alternatively using electronic or chemical ionization where necessary. Melting points (m.p.) were obtained from recrystallized samples using a Leica Galen III heated-stage microscope and are uncorrected.

## Supplementary information

Supplementary Information

## Data Availability

All reported data are freely available from the authors on request. Correspondence and requests for materials should be addressed to S.P.F. The source data underlying Figs. 2b, c, 3b–d, 4a–d, 5b, c, and Supplementary Figs. 29–31 and 33–42 are provided as a Source Data file.

## Source data

Source Data
