# Supplementary material for: Selection from a pool of self-assembling lipid replicators
Source: Nat Commun. 2020 Jan 10;11:176. doi: 10.1038/s41467-019-13903-x (PMC6954257; doi:10.1038/s41467-019-13903-x)
Supplement: Supplementary file 1 — Supplementary Information [file 41467_2019_13903_MOESM1_ESM.pdf]

Supplementary Information

Selection from a pool of self-assembling lipid replicators

Colomer et al.

Supplementary Information for

Selection from a pool of self-assembling lipid replicators

Ignacio Colomer, Arseni Borissov and Stephen P. Fletcher

Table of Contents

Supplementary Figures 3

Supplementary Methods 14

General experimental details 14

Synthesis of compounds 15

Kinetic analysis 28

Seeding experiment of the system **2c** / **3c** with 20 mol% of **3c**. 28

Seeding experiment of the system **2c** / **3c** with 20 mol% of **3a**. 29

Continuous stirring tank reactor (CSTR) experiment details 31

Kinetic study of replicator destruction 34

Kinetic study to determine rate and rate constants 35

Critical micelle concentration (CMC) determination 40

Particle size measure using DLS 42

TEM experiments 47

Supplementary References 49

Supplementary Figures

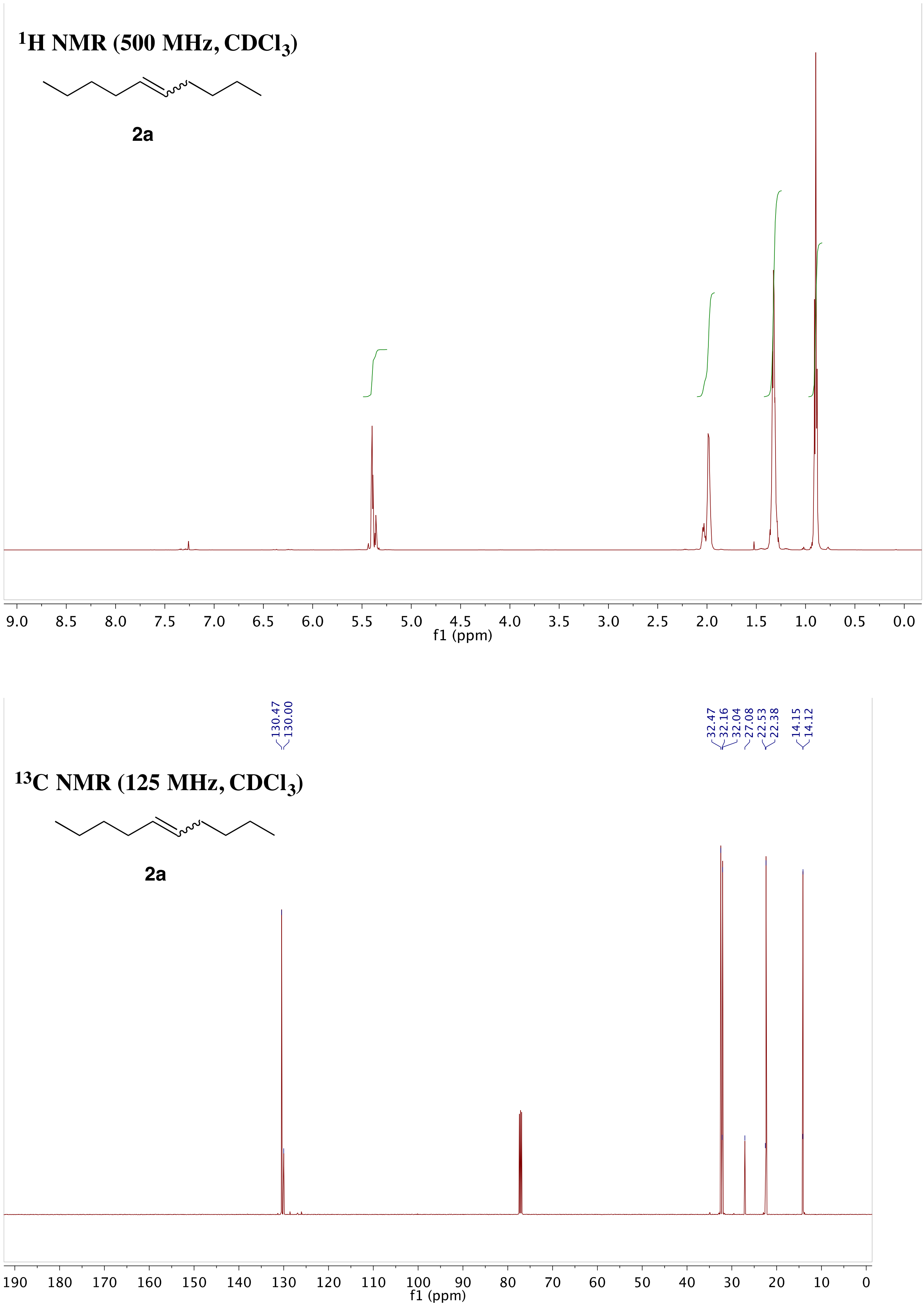

Supplementary Figure 1 ^{1}H NMR and ^{13}C NMR spectra of **2a**.

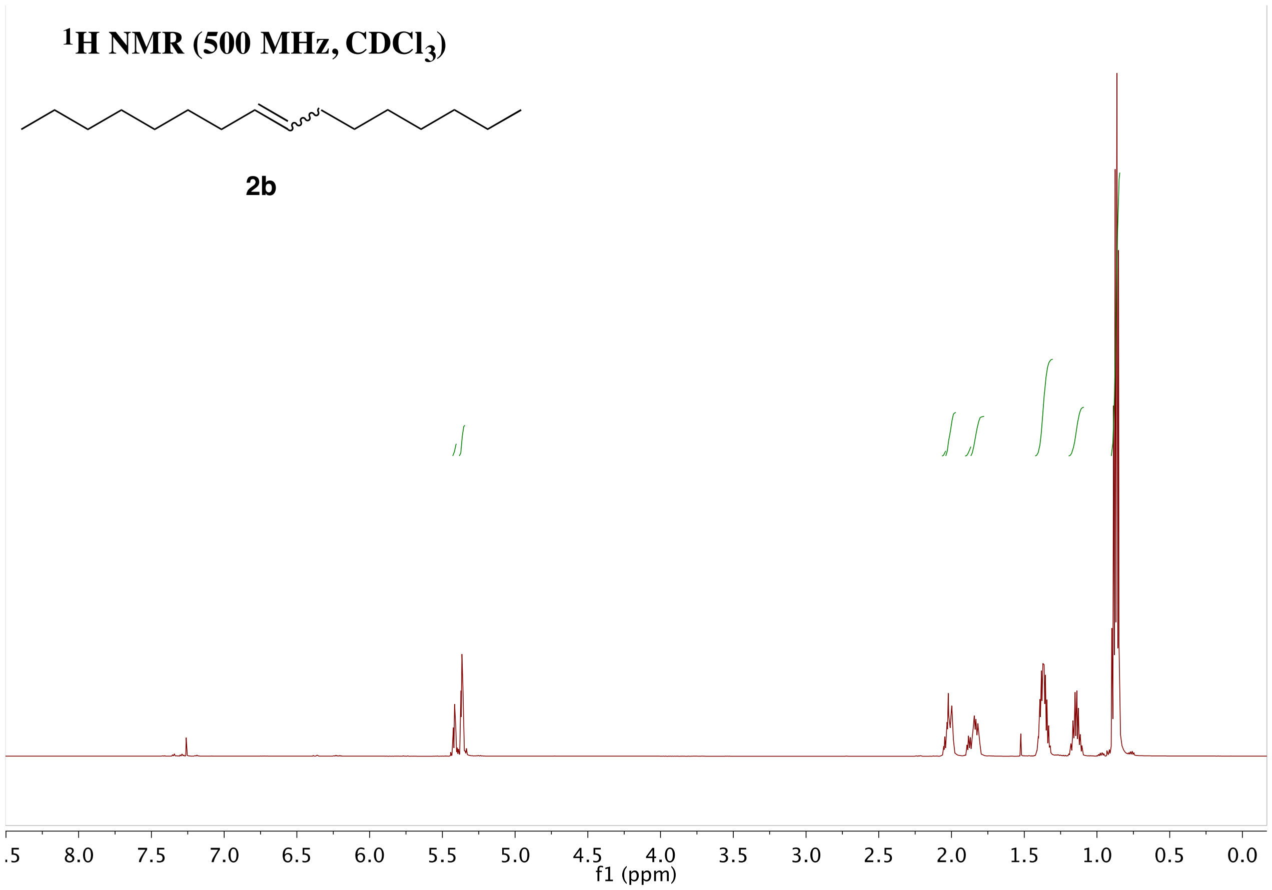

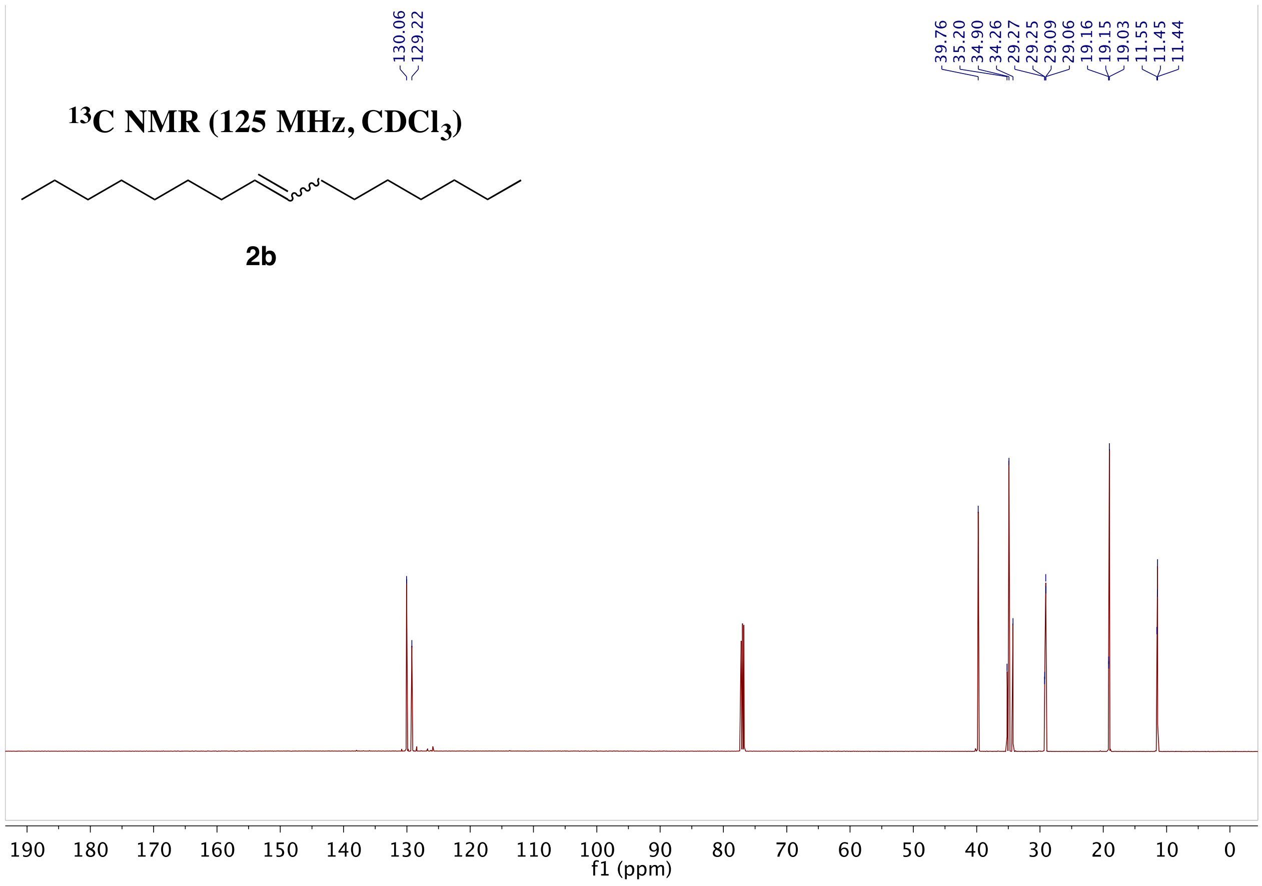

Supplementary Figure 2 ^{1}H NMR and ^{13}C NMR spectra of **2a**.

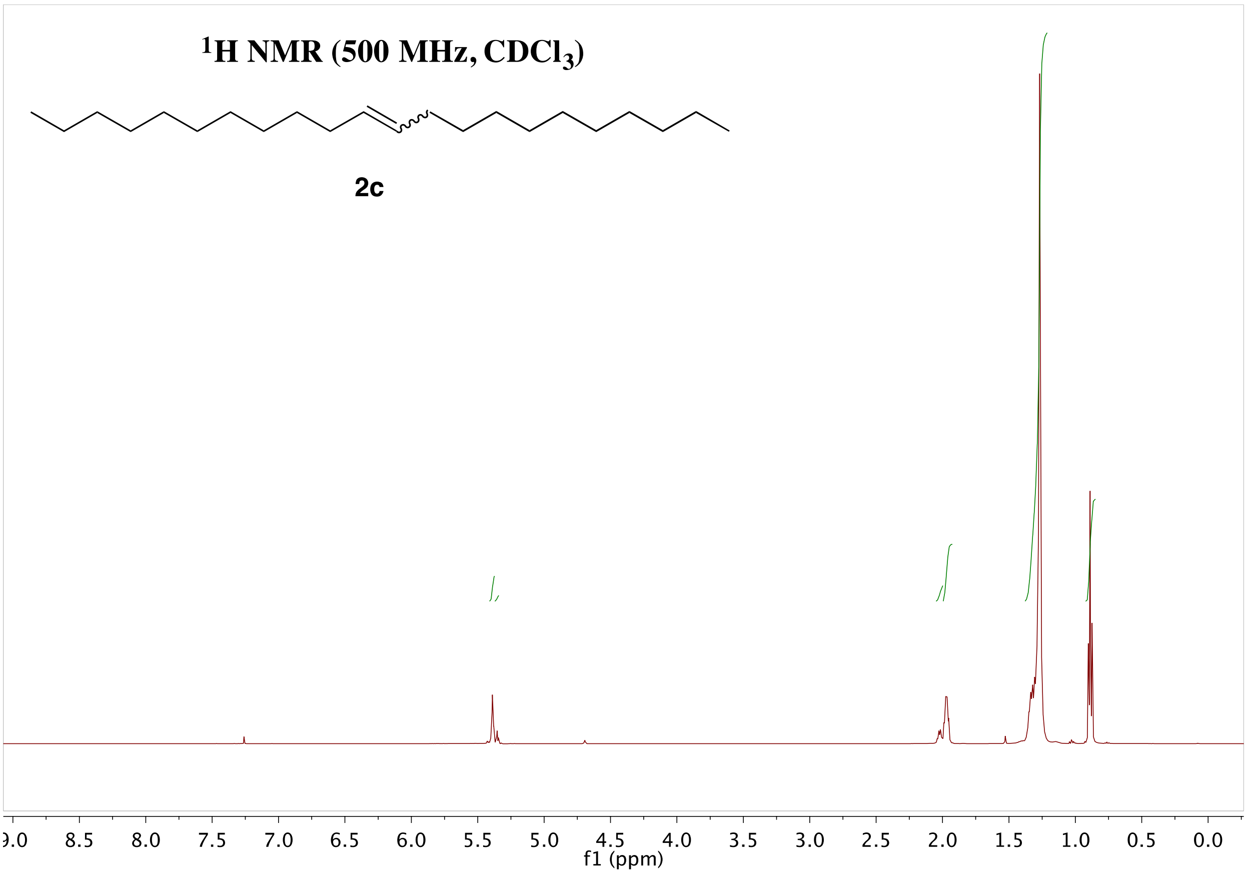

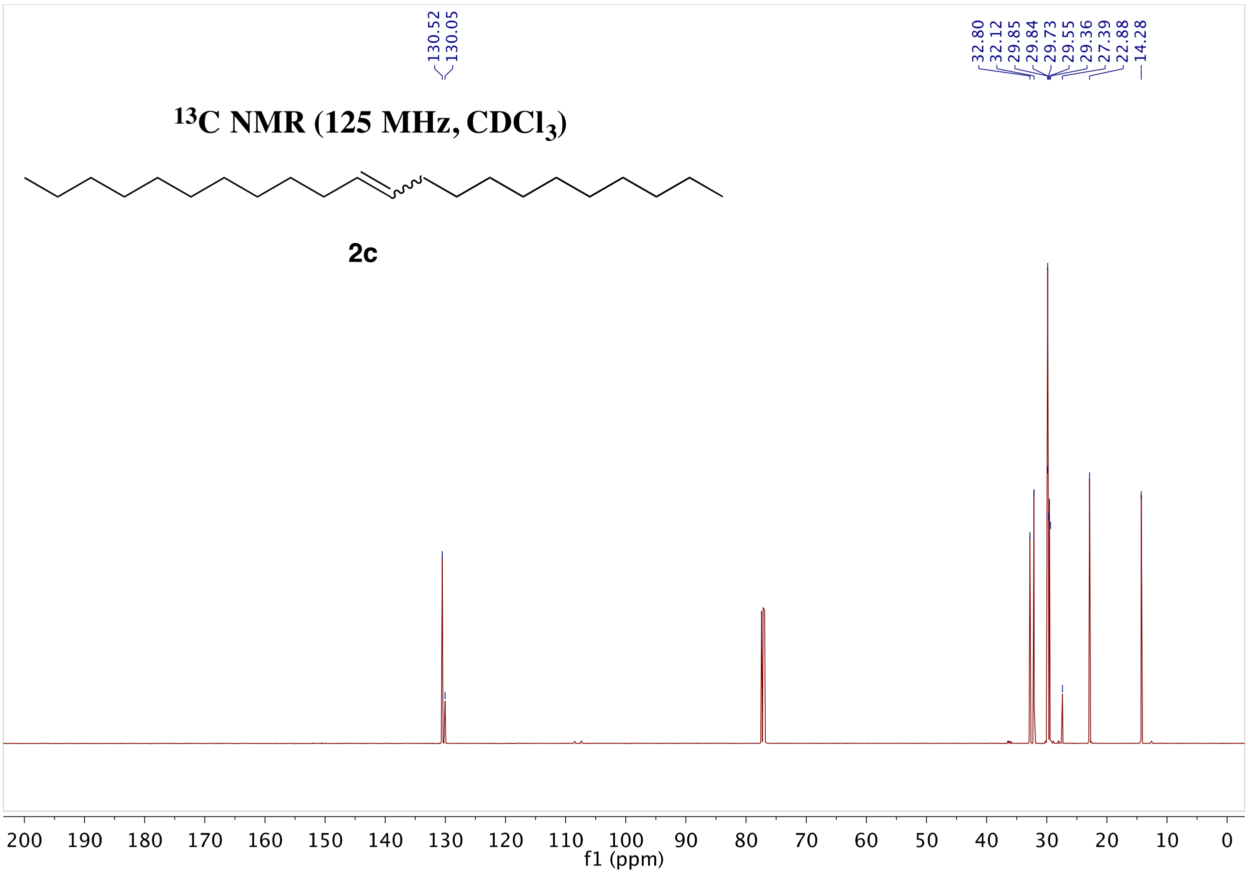

Supplementary Figure 3 ^{1}H NMR and ^{13}C NMR spectra of **2c**.

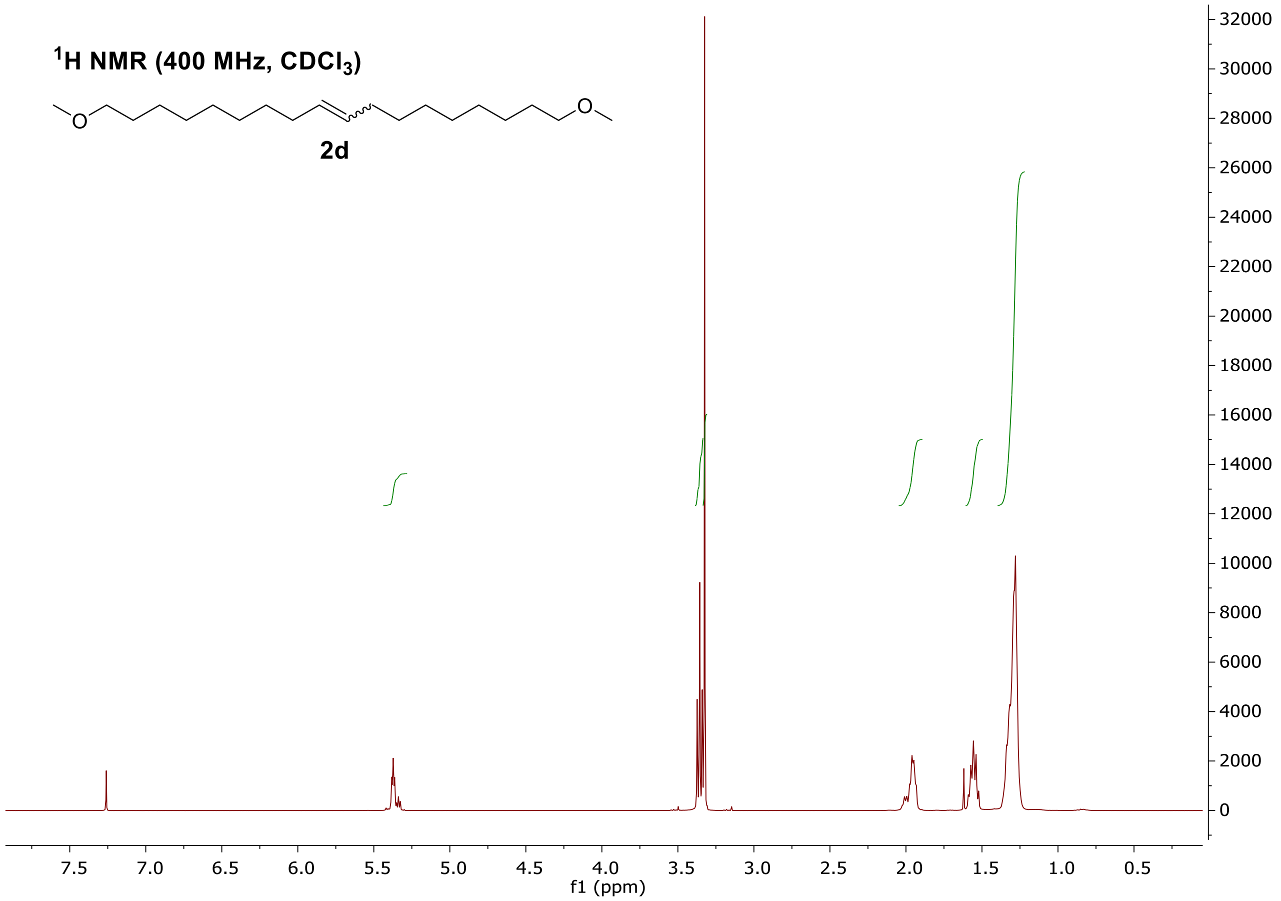

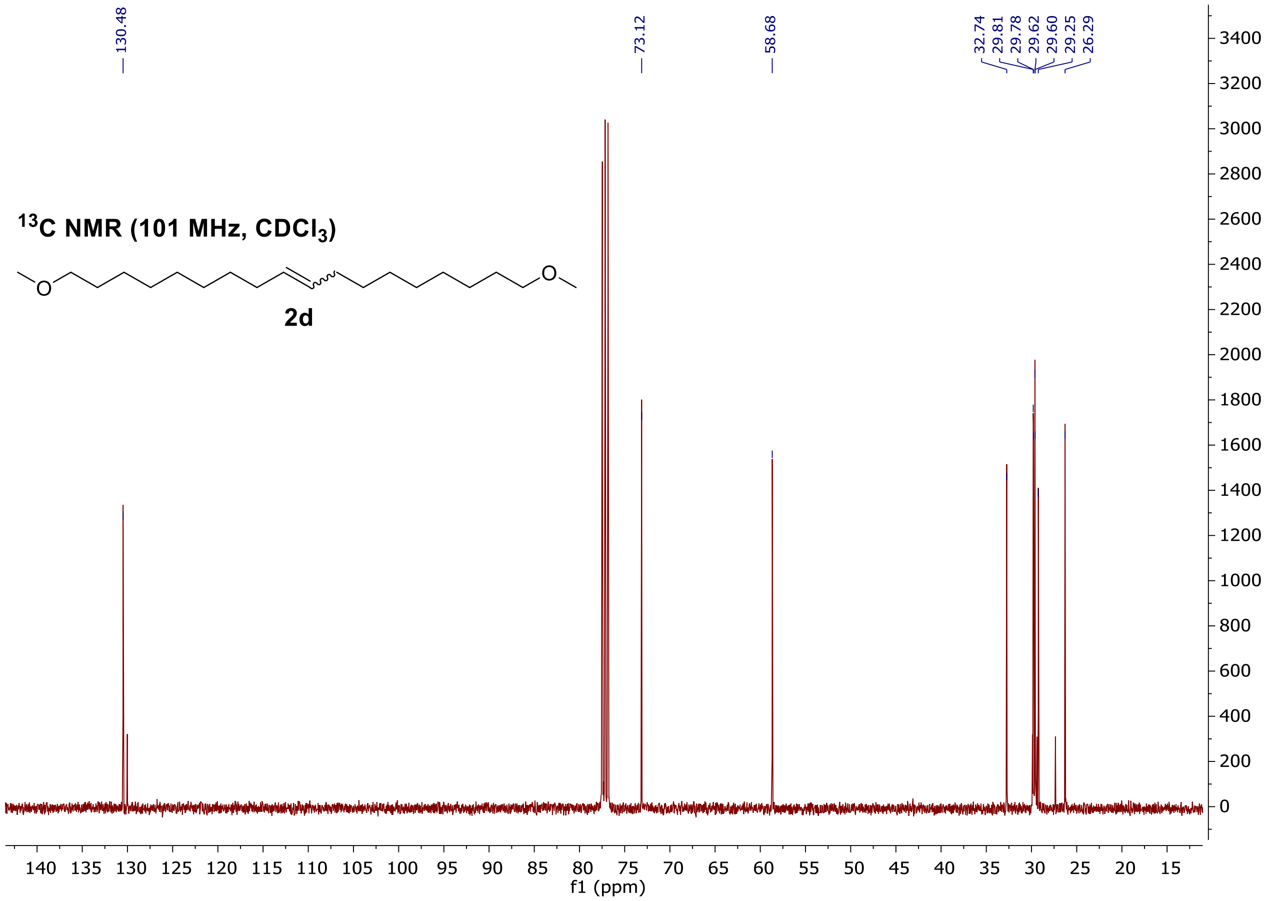

Supplementary Figure 4 ^{1}H NMR and ^{13}C NMR spectra of **2d**.

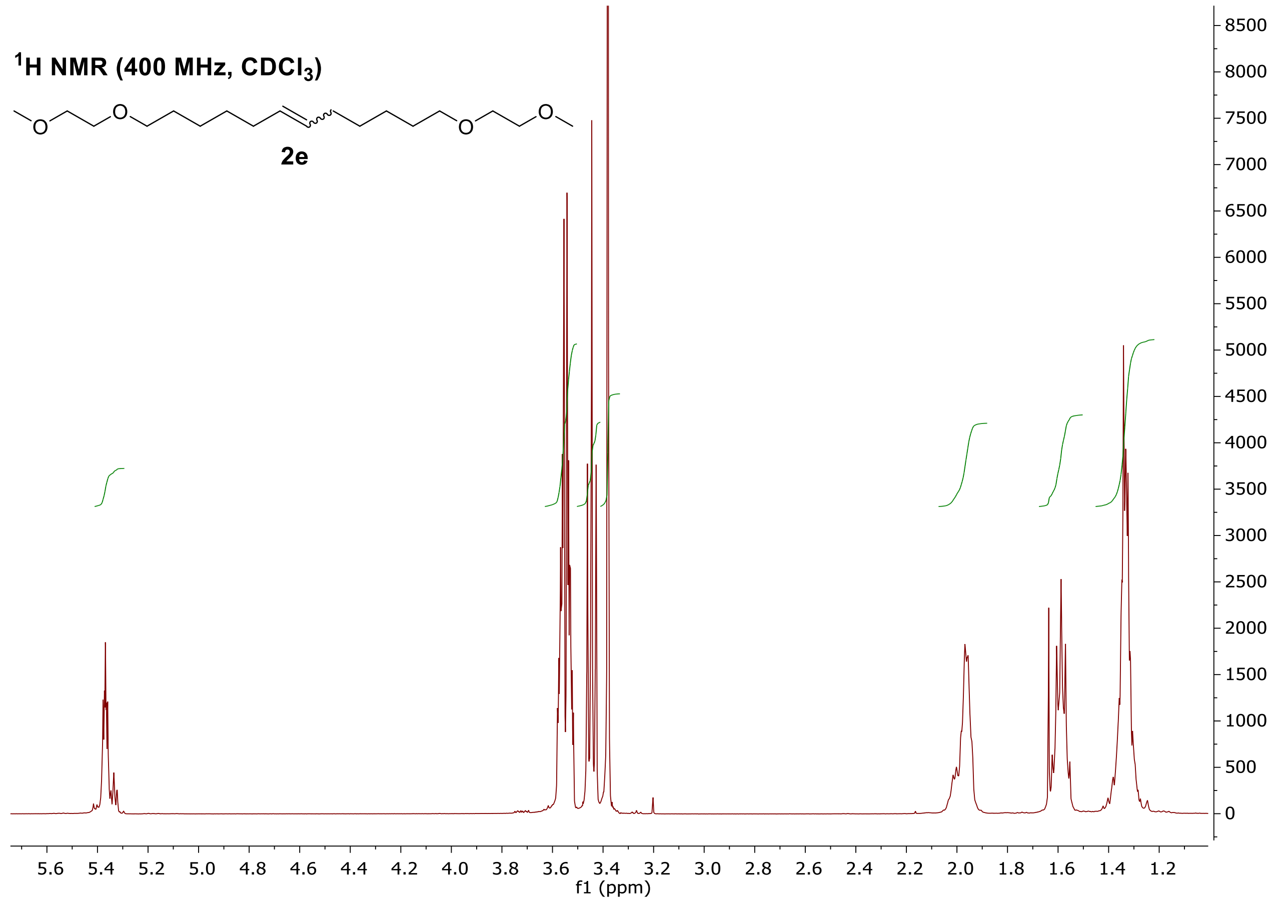

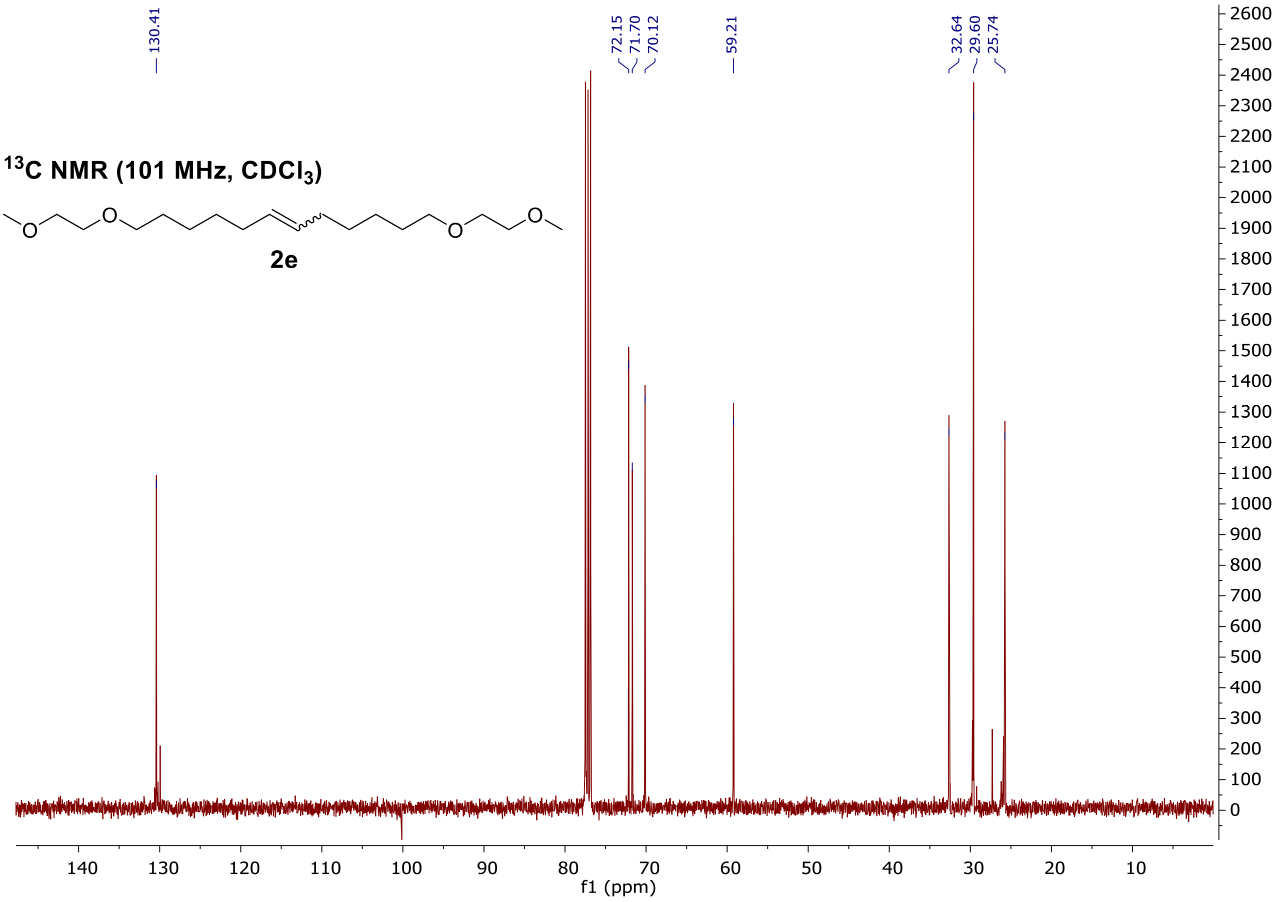

Supplementary Figure 5 ^{1}H NMR and ^{13}C NMR spectra of **2e**.

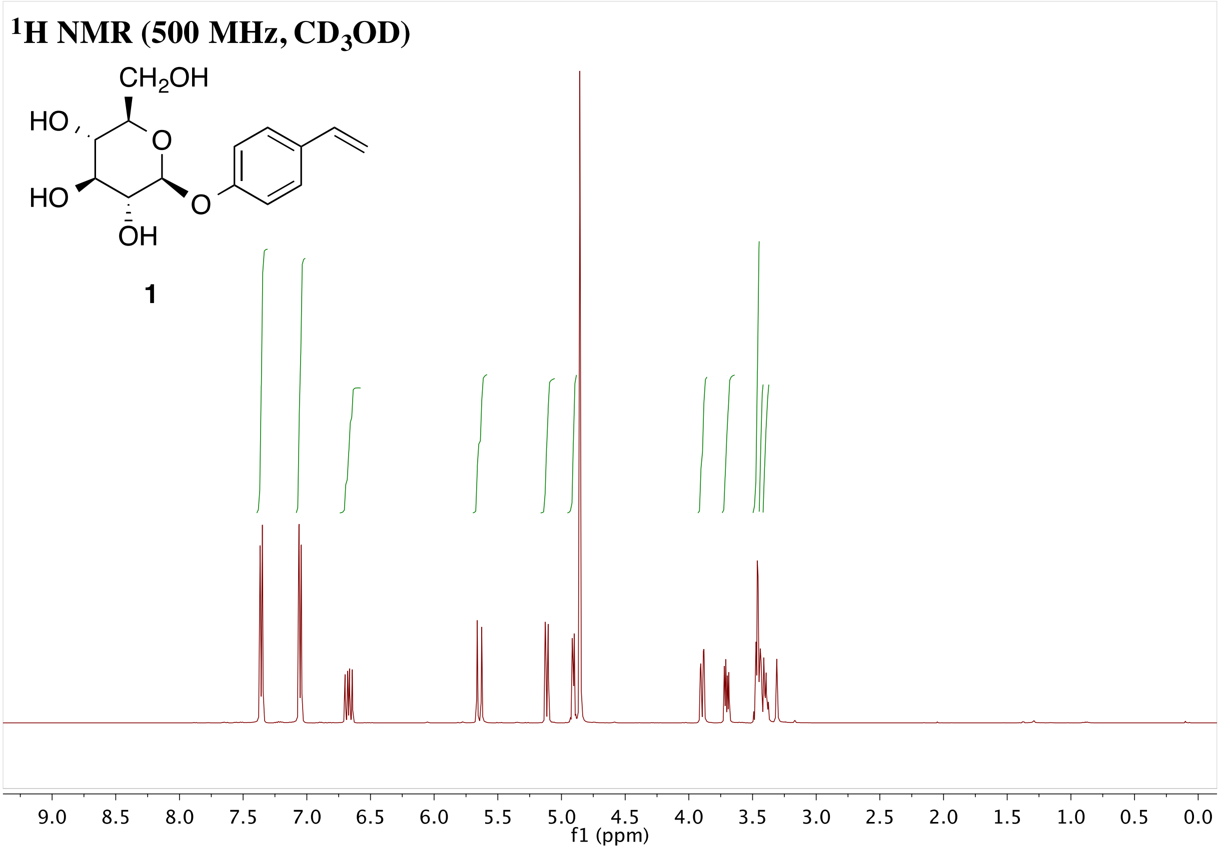

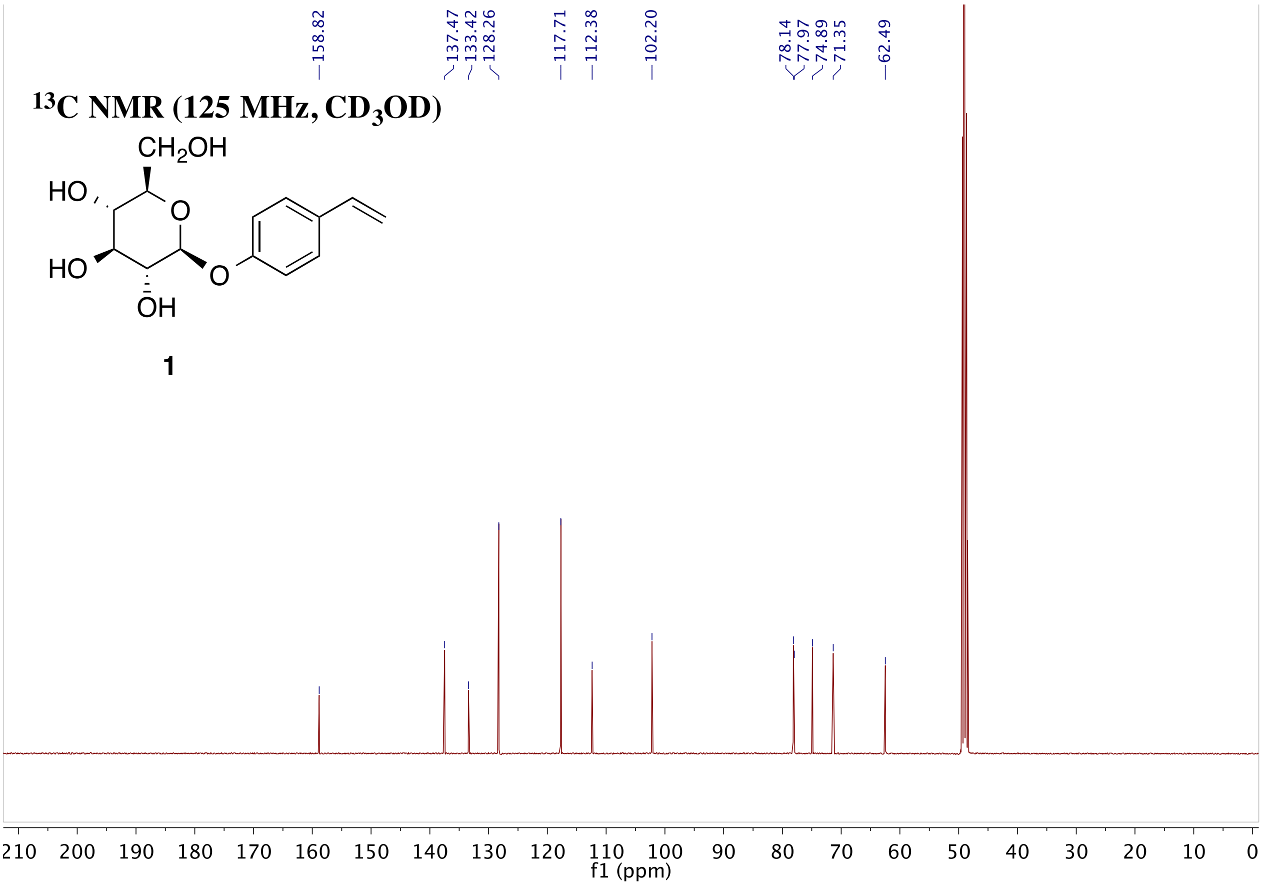

Supplementary Figure 6 ^{1}H NMR and ^{13}C NMR spectra of **1**.

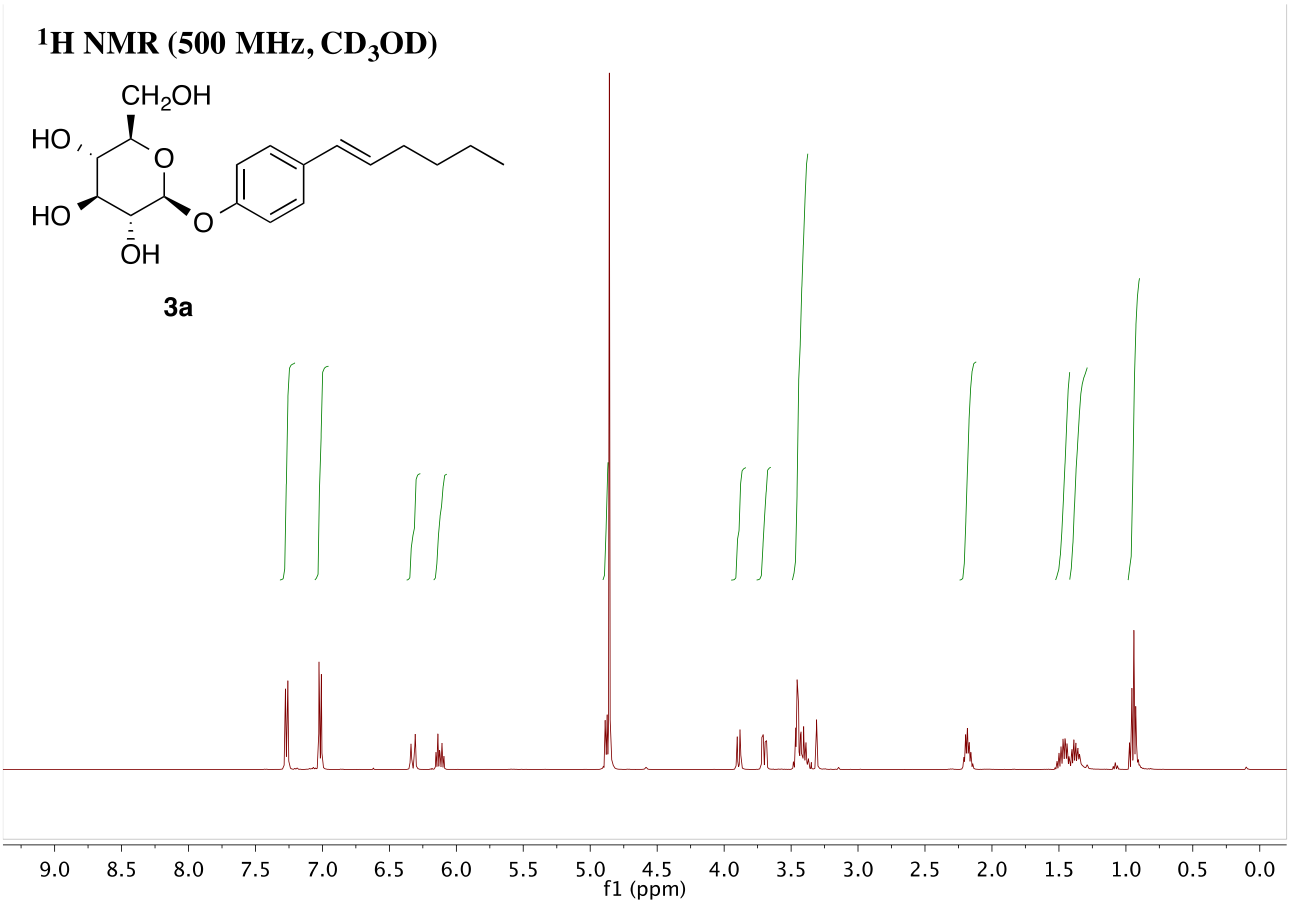

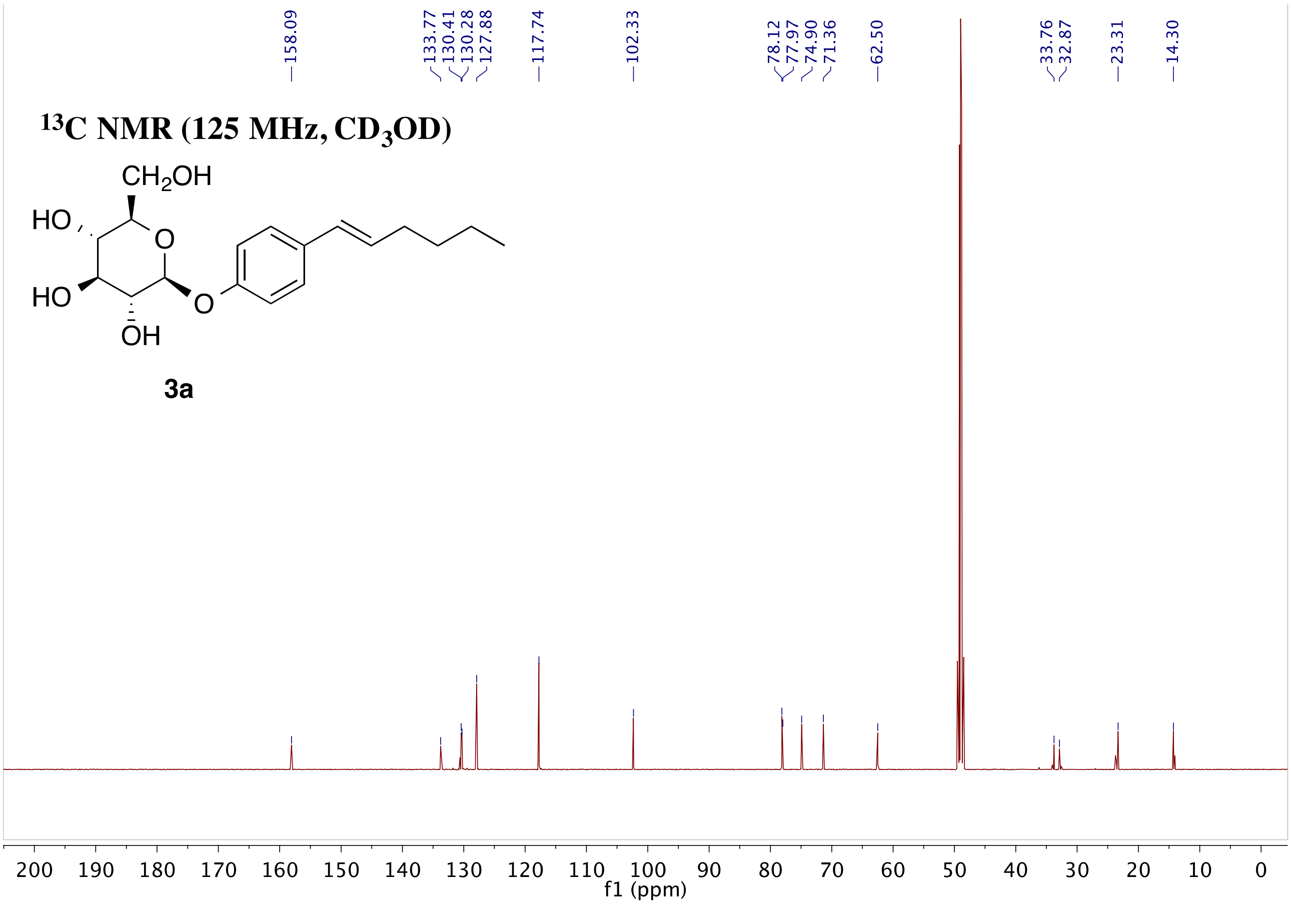

Supplementary Figure 7 ^{1}H NMR and ^{13}C NMR spectra of **3a**.

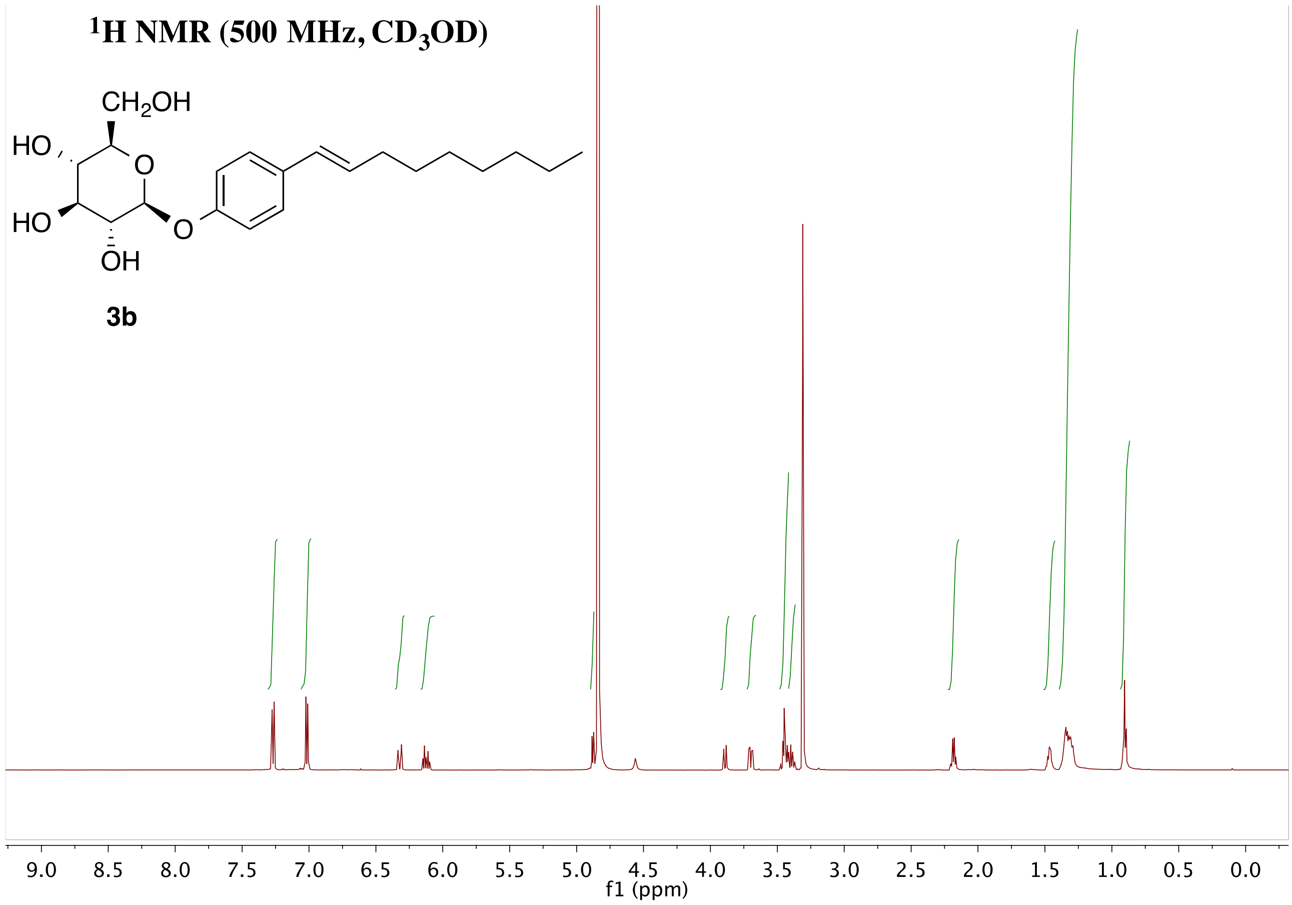

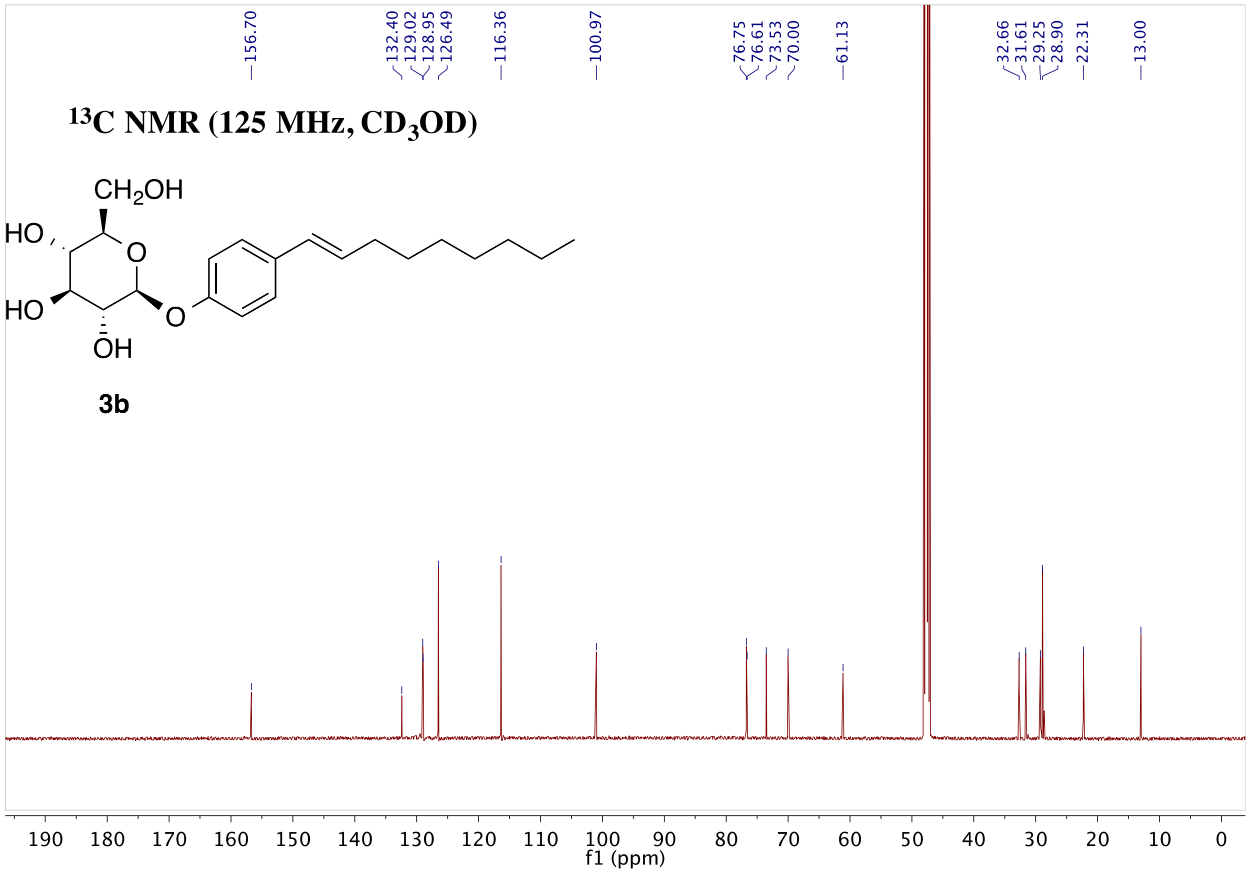

Supplementary Figure 8 ^{1}H NMR and ^{13}C NMR spectra of **3b**.

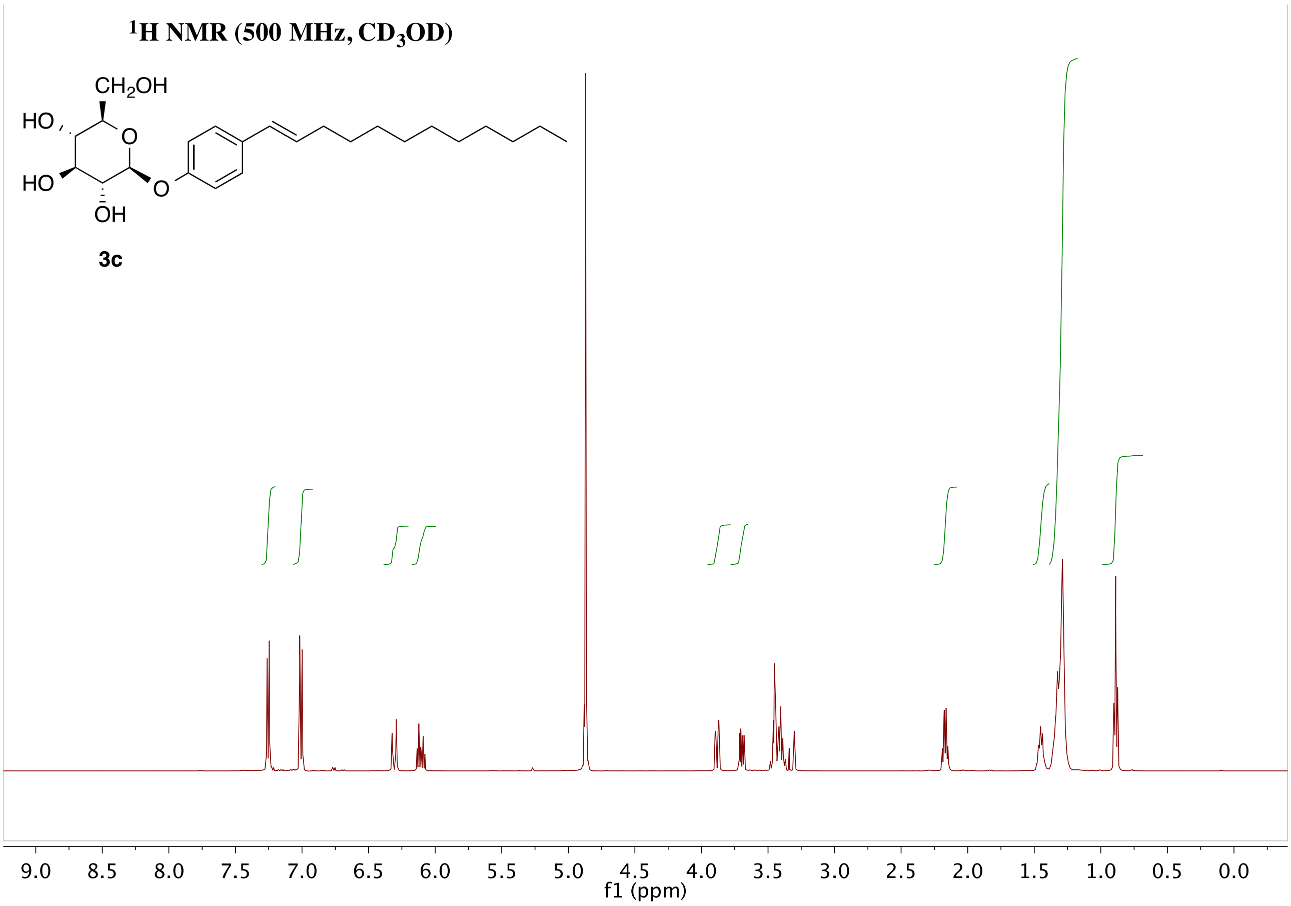

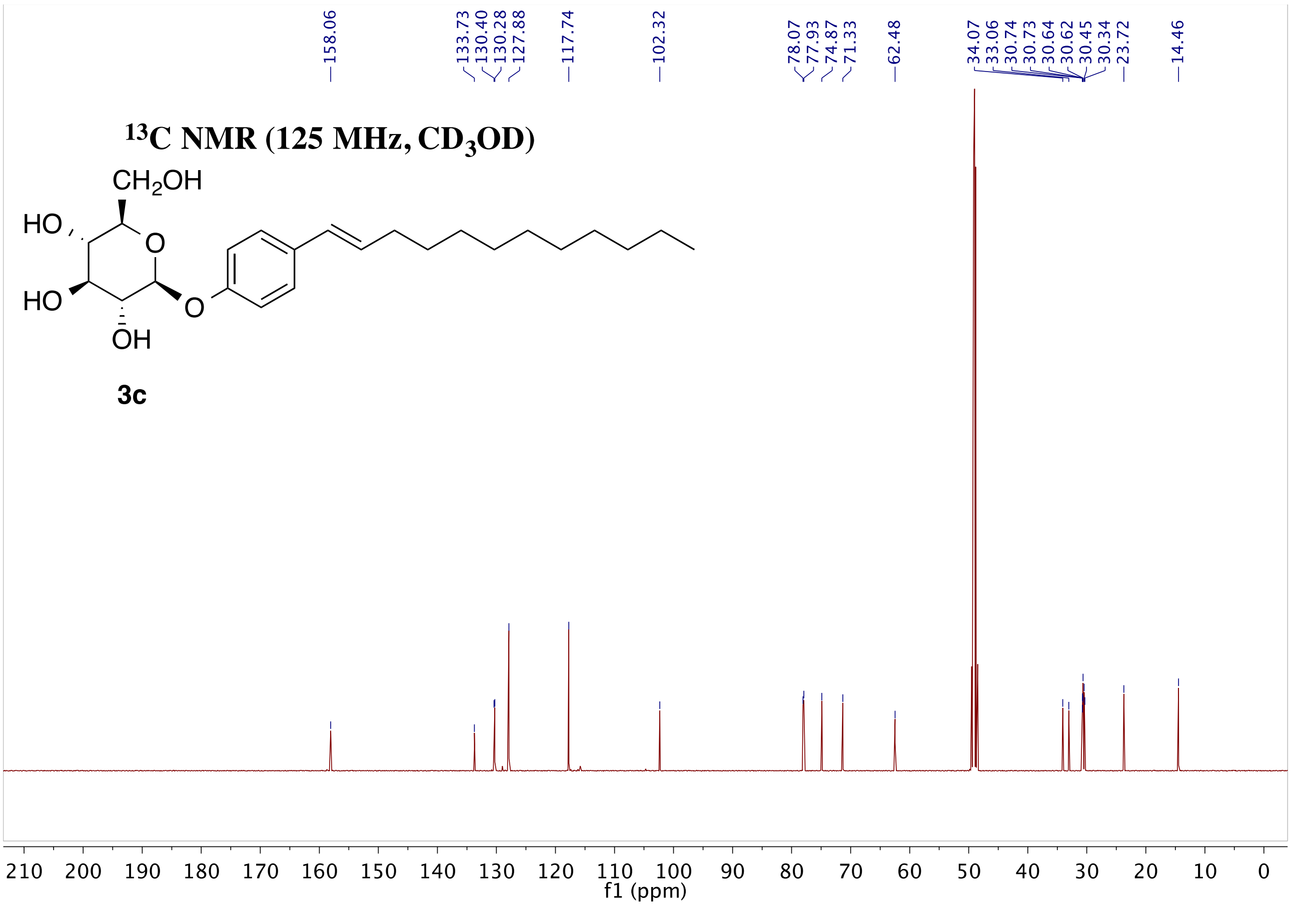

Supplementary Figure 9 ^{1}H NMR and ^{13}C NMR spectra of **3c**.

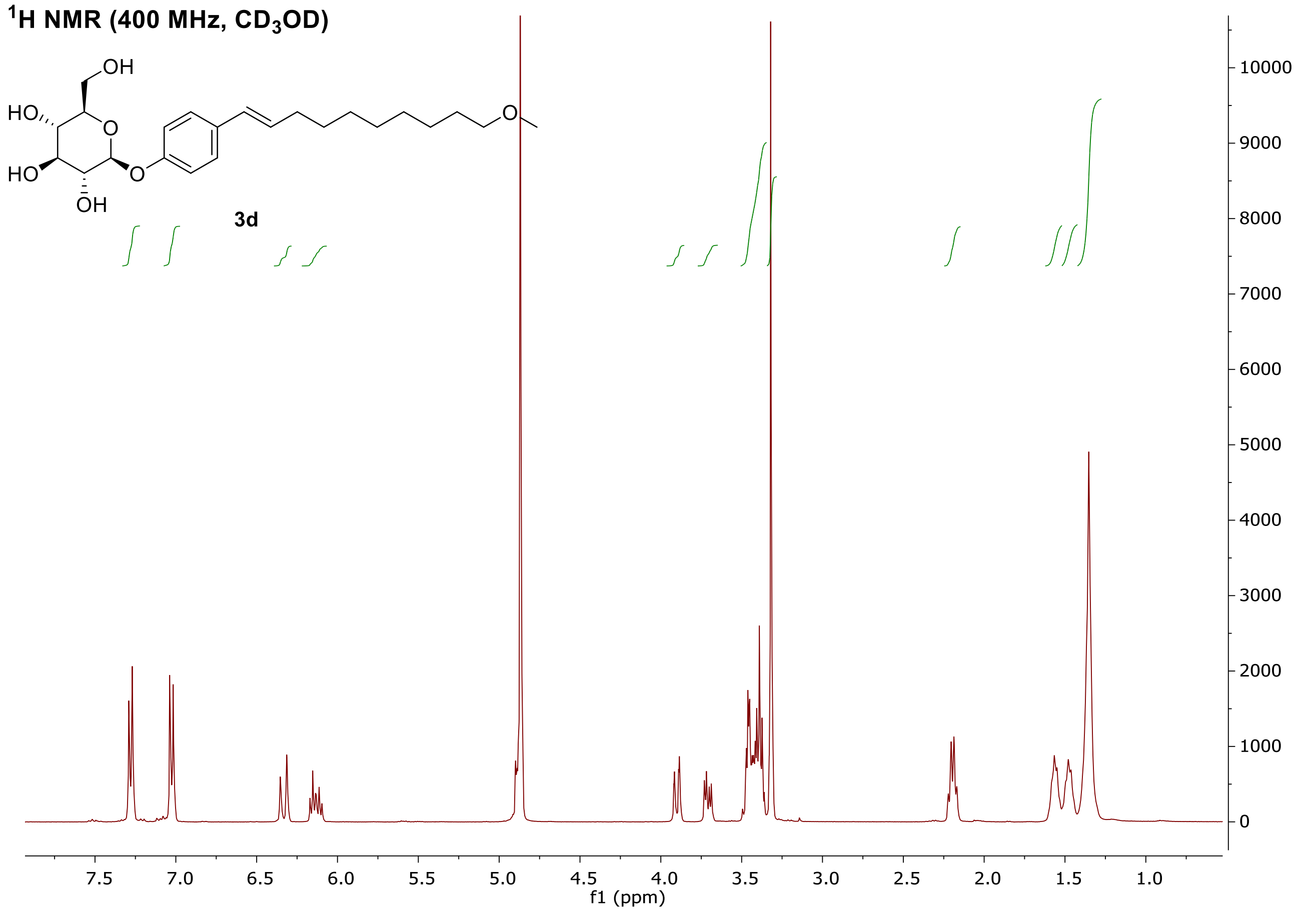

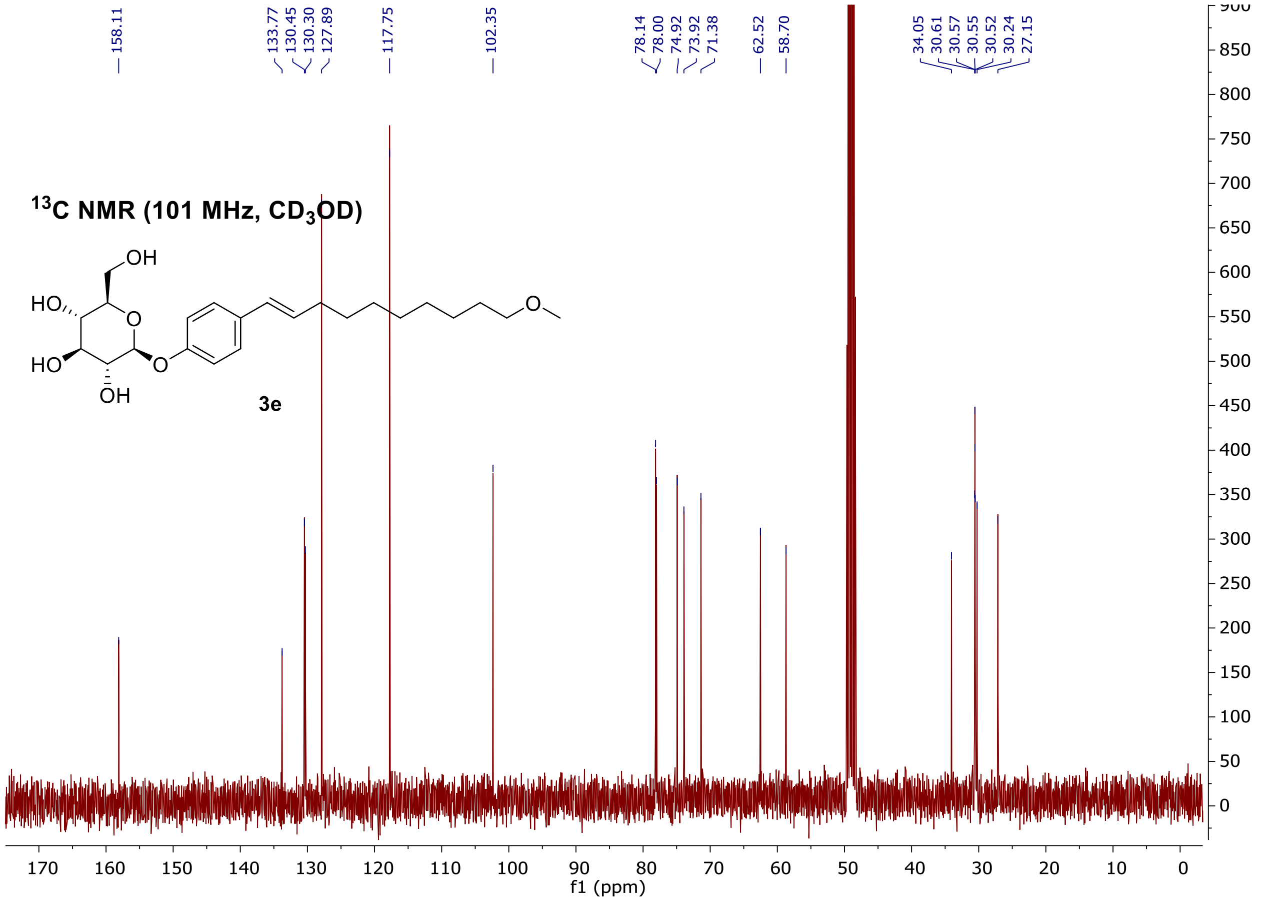

Supplementary Figure 10 ^{1}H NMR and ^{13}C NMR spectra of **3d** .

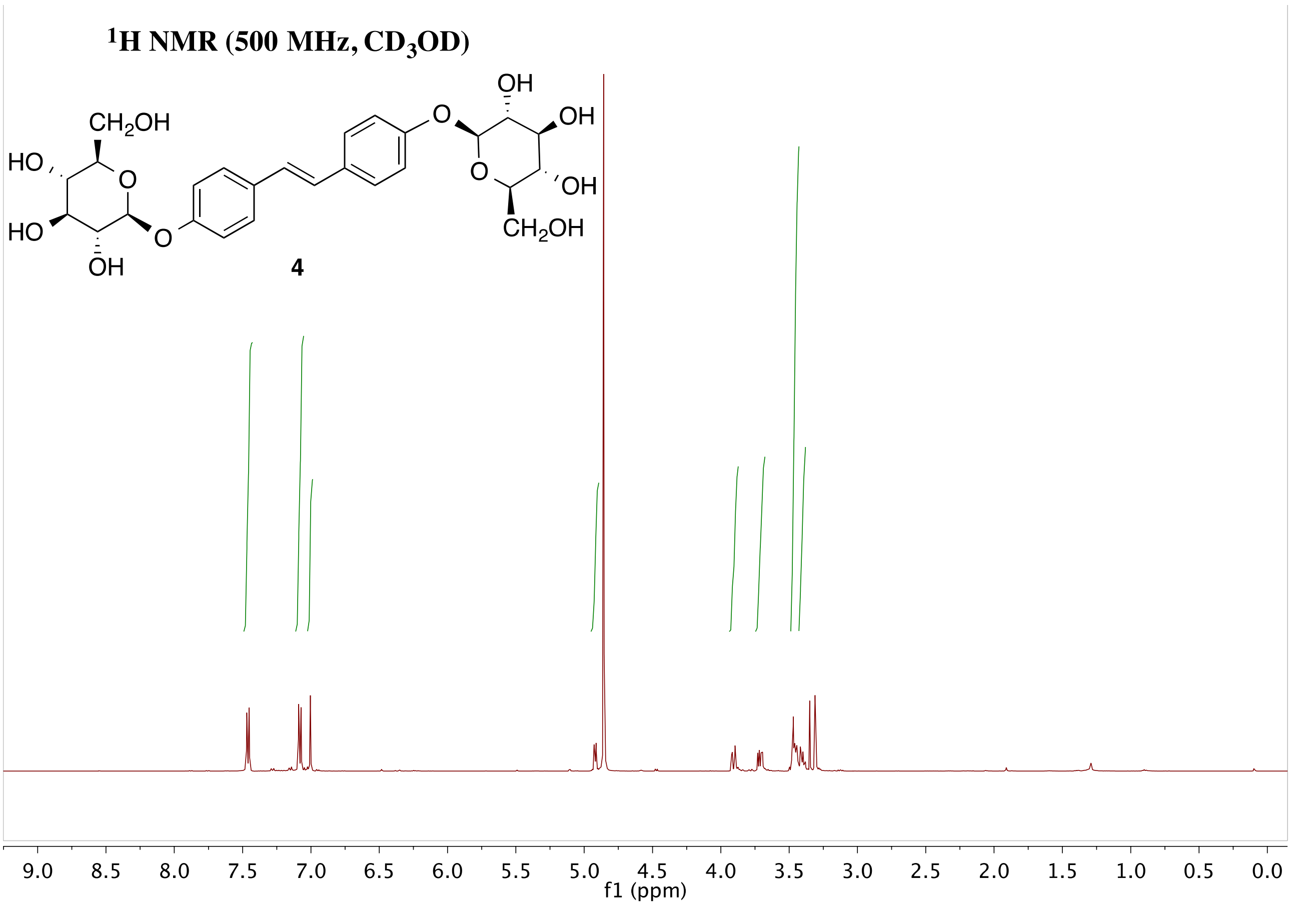

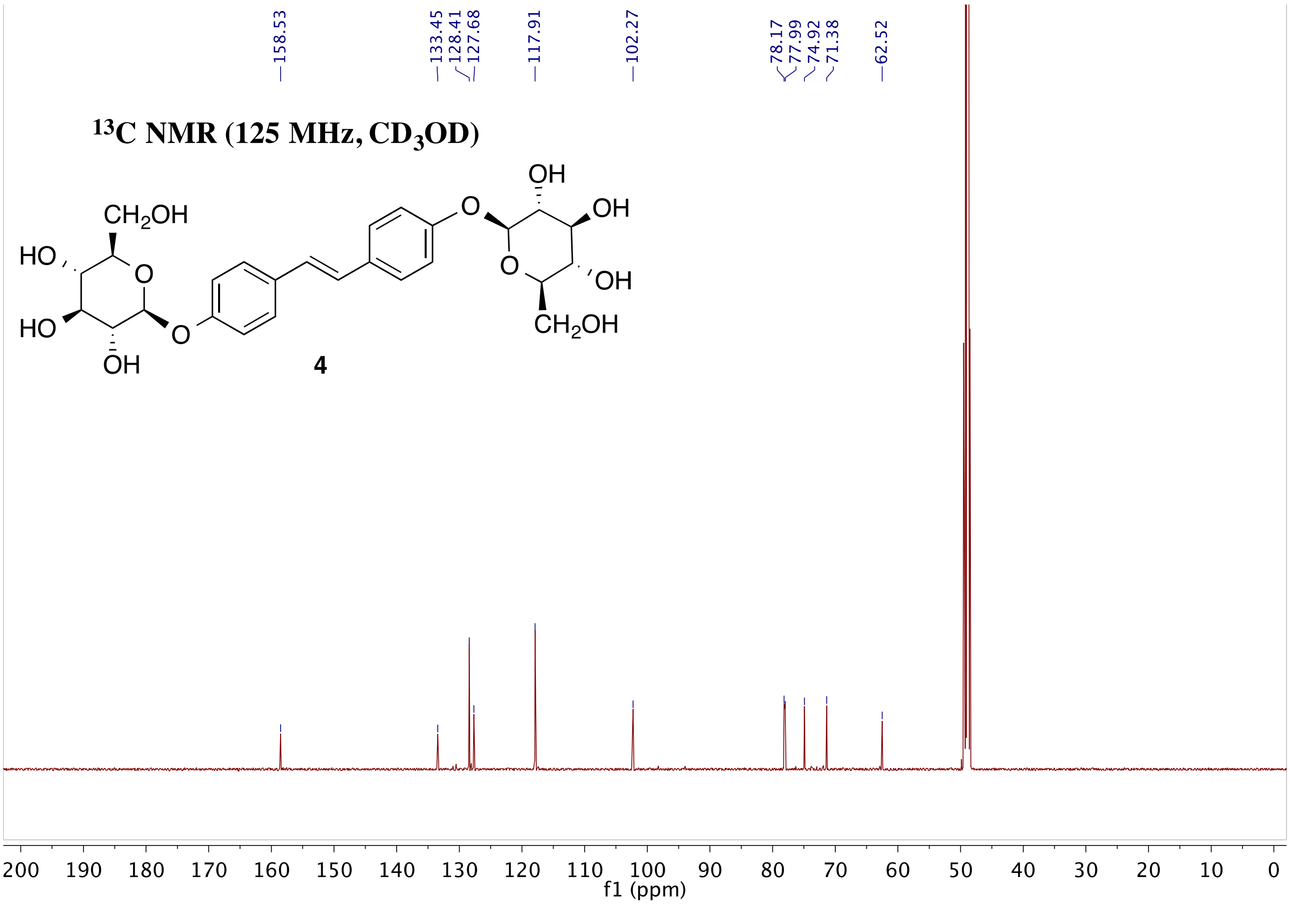

Supplementary Figure 11 ^{1}H NMR and ^{13}C NMR spectra of **4**.

Supplementary Methods

General experimental details

^{1}H NMR and ^{13}C NMR spectra were recorded on a 400 MHz or 500MHz spectrometer in $CDCl_3$ or CD_3OD and referenced to residual solvent peaks. Chemical shifts are quoted in ppm (parts per million) to the nearest 0.01 ppm with signal splitting recorded as singlet (s), doublet (d), triplet (t), quartet (q), quintet (quint), septet (sept.), multiplet (m) and broad singlet (br. s). Coupling constants, *J*, are measured in Hz to the nearest 0.1 Hz. ^{1}H and ^{13}C NMR spectra were recorded at room temperature. NMR signals were assigned by referring to COSY, HSQC and where necessary HMBC spectra. Infrared spectra were recorded as thin films of neat samples on a Bruker Tensor 27 FT-IR spectrometer equipped with Attenuated Total Reflectance sampling accessories. High resolution mass spectra are given to four decimal places and were recorded on a Bruker MicroTof (resolution = 10000 FWHM) under conditions of electrospray ionization (ESI), electronic ionization (EI) or chemical ionization (CI). Optical rotations were measured at 25 ºC using a sodium lamp in the appropriate solvent. Melting points (m.p.) were obtained from recrystallized samples using a Lecia VMTG heated-stage microscope and are uncorrected. The solvent systems used for recrystallization are quoted in parentheses. Flash column chromatography was performed using silica gel (60 Å, 0.033-0.070 mm, BDH). TLC analyses were performed on Merck Kiesegel 60 F_{254} 0.25 mm precoated silica plates. Reagents obtained from Sigma-Aldrich, Alfa, Fluorochem and TCI suppliers were used directly as supplied. All anhydrous reactions were carried out in flame-dried glassware and under an inert atmosphere of argon provided by a balloon. All reactions were stirred with magnetic followers.

Synthesis of compounds

General procedure for homogeneous alkene cross-metathesis: To a flame-dried flask, charged with a catalytic amount of Ru-catalyst, under Ar, at room temperature, was added 4.0 mL/mmol of dry solvent (previously degassed, bubbling Ar over 30 min.). A solution of the mixture of alkenes in dry and degassed solvent (1.0 mL/mmol) was added and the mixture was stirred at the appropriate temperature. The reaction was monitored by TLC until completion, and the solvent was evaporated under reduced pressure to give the corresponding alkene, which was purified by chromatography on silica gel using the appropriate mixture of eluents.

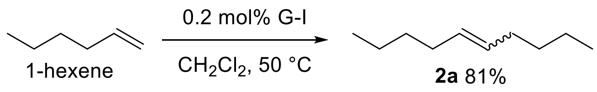

Supplementary Figure 12 Synthesis of 5-decene **2a**. See below for details.

From 1-hexene (12.412 mL, 100 mmol) and Grubbs 1st generation catalyst (165 mg, 0.2 mmol), in 40 mL of CH_2Cl_2, following the general procedure, a 4.7:1 *E*:*Z* mixture of alkene **2a** was obtained. The product was purified by distillation (25 mbar) to provide **2a** (5.650 g, 40.28 mmol, 81%), as a colourless oil. Spectroscopic properties matched those previously reported.[1]

Data for **2a**: ***R_f*** 0.80 (pentane). **^{1}H NMR (500 MHz, $CDCl_3$)** δ 5.38-5.41 (2 H, m, C*H*=C*H* *E* isom), 5.35-5.37 (2 H, m, C*H*=C*H* *Z* isom), 2.01-2.06 (4 H, m, 2 × CH_2-CH=CH *Z* isom), 1.96-2.01 (4 H, m, 2 × CH_2-CH=CH *E* isom), 1.29-1.36 (8 H, m, 4 × CH_2), 0.91 (6 H, t, *J* = 7.1 Hz, 2 × CH_3 *Z* isom), 0.90 (6 H, t, *J* = 7.2 Hz, 2 × CH_3 *E* isom). **^{13}C NMR (125 MHz, $CDCl_3$)** δ 130.5 (2C, *C*H=*C*H *E* isom), 130.0 (2C, *C*H=*C*H *Z* isom), 32.5 (2C, *C*H_2-CH=CH-*C*H_2 *E* isom), 32.2 (2C, 2 × CH_2 *Z* isom), 32.0 (2C, 2 x CH_2 *E* isom), 27.1 (2C, *C*H_2-CH=CH-*C*H_2 *Z* isom), 22.5 (2C, 2 × CH_2 *Z* isom), 22.4 (2C, 2 × CH_2 *E* isom), 14.2 (2C, 2 × CH_3 *Z* isom), 14.1 (2C , 2 × CH_3 *E* isom). **HRMS** (EI): calculated for $C_{10}H_{20}$ $[M]^+$ requires *m*/*z* 140.1560, found *m*/*z* 140.1556.

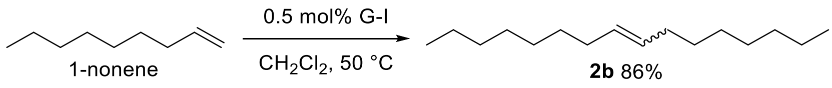

Supplementary Figure 13 Synthesis of 8-hexadecene **2b**. See below for details.

From 1-nonene (20.0 mL, 115 mmol) and Grubbs 1st generation catalyst (475 mg, 0.58 mmol), in 50 mL of CH_2Cl_2, following the general procedure, a 4.5:1 *E*:*Z* mixture of alkene **2b** was obtained. Chromatographic purification (pentane) gave a 4.5:1 *E*:*Z* mixture of alkene **2b** (22 g, 86%), as a colorless oil. Spectroscopic properties matched those previously reported.[2]

Data for **2b**: R_f 0.80 (pentane). **^{1}H NMR (500 MHz, $CDCl_3$)** δ 5.40-5.43 (2 H, m, C*H*=C*H* *Z* isom), 5.35-5.38 (2 H, m, C*H*=C*H* *E* isom), 2.03-2.07 (4 H, m, 2 × CH_2-CH=CH *Z* isom), 1.98-2.03 (4 H, m, 2 × CH_2-CH=CH *E* isom), 1.87-1.91 (4 H, m, 2 × CH_2 *Z* isom), 1.80-1.86 (4 H, m, 2 × CH_2 *E* isom), 1.31-1.44 (12 H, m, 6 x CH_2), 1.10-1.19 (4 H, m, 2 x CH_2), 0.89 (6 H, t, *J* = 6.3 Hz, 2 × CH_3 *Z* isom), 0.86 (6 H, t, *J* = 6.4 Hz, 2 × CH_3 *E* isom). **^{13}C NMR (125 MHz, $CDCl_3$)** δ 130.1 (2C, *C*H=*C*H *E* isom), 129.2 (2C, *C*H=*C*H *Z* isom), 39.8 (2C, 2 × CH_2 *E* isom), 35.2 (2C, 2 × CH_2 *Z* isom), 34.9 (4C, 4 x CH_2 *E* isom), 34.3 (4C, 4 x CH_2 *Z* isom), 29.3 (4C, 4 × CH_2 *Z* isom), 29.1 (4C, 4 × CH_2 *E* isom), 19.2 (2C, 2 × CH_2 *Z* isom), 19.0 (2C, 2 × CH_2 *E* isom), 11.6 (2C, 2 × CH_3 *Z* isom), 11.4 (2C, 2 × CH_3 *E* isom). **HRMS** (EI): calculated for $C_{16}H_{32}$ $[M]^+$ requires *m*/*z* 224.2504, found *m*/*z* 224.2501.

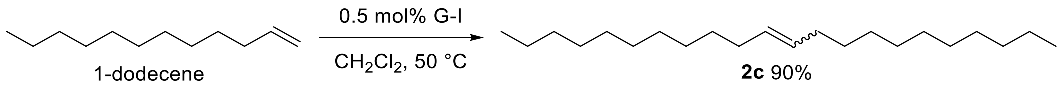

Supplementary Figure 14 Synthesis of 11-docosene, **2c**. See below for details.

From 1-dodecene (20 mL, 90 mmol) and Grubbs 1st generation catalyst (370 mg, 0.45 mmol), in 50 mL of CH_2Cl_2, following the general procedure, a 4.2:1 *E*:*Z* mixture of alkene **2c** was obtained. Chromatographic purification (pentane) gave a 4.2:1 *E*:*Z* mixture of alkene **2c** (24.9 g, 90%), as a colourless oil. Spectroscopic properties matched those previously reported.[3]

Data for **2c**: R_f 0.80 (pentane). **^{1}H NMR (500 MHz, $CDCl_3$)** δ 5.38-5.40 (2 H, m, C*H*=C*H* *E* isom), 5.34-5.37 (2 H, m, C*H*=C*H* *Z* isom), 2.00-2.05 (4 H, m, 2 × CH_2-CH=CH *Z* isom), 1.94-2.00 (4 H, m, 2 × CH_2-CH=CH *E* isom), 1.24-1.36 (32 H, m, 16 x CH_2), 0.89 (6 H, t, *J* = 6.9 Hz, 2 × CH_3). **^{13}C NMR (125 MHz, $CDCl_3$)** δ 130.5 (2C, *C*H=*C*H *E* isom), 130.1 (2C, *C*H=*C*H *Z* isom), 32.8 (2C, *C*H_2-CH=CH-*C*H_2 *E* isom), 32.1 (2C, 2 × CH_2), 29.9 (4C, 4 × CH_2), 29.8 (2C, 2 × CH_2), 29.7 (2C, 2 × CH_2), 29.6 (2C, 2 × CH_2), 29.4 (2C, 2 × CH_2), 27.4 (2C, *C*H_2-CH=CH-*C*H_2 *Z* isom), 22.9 (2C, 2 × CH_2), 14.3 (2C, 2 × CH_3). **HRMS** (EI): calculated for $C_{22}H_{44}$ $[M]^+$ requires *m*/*z* 308.3438, found *m*/*z* 308.3440.

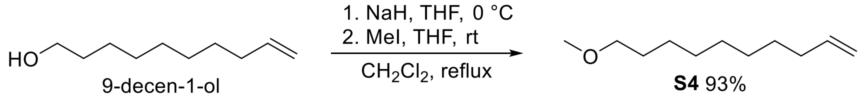

Supplementary Figure 15 Synthesis of **S4**. See below for details.

9-decen-1-ol (5 g, 32 mmol) was added to dry THF (60 ml) and cooled to 0 °C. NaH (60% in mineral oil, 1.920 g, 48 mmol) was added and the mixture stirred at 0 °C for 30 min. MeI (3 ml, 48 mmol) was then added and the reaction was stirred at rt overnight. Excess NaH was quenched by slow addition of MeOH, the mixture was then concentrated and partitioned in water and CH_2Cl_2. Phases were separated, the organic phase was dried with anhydrous Na_2SO_4, decanted and concentrated under vacuum. Crude product was purified by silica chromatography (0-15% EtOAc/petrol) to provide **S4** (5.073 g, 29.79 mmol, 93%) as a colourless oil.

Data for **S4**: **^{1}H NMR (400 MHz, $CDCl_3$)** δ 5.81 (ddt, J = 16.9, 10.2, 6.7 Hz, 1H, C*H*=CH_2), 4.99 (ddt, J = 17.1, 2.3, 1.6 Hz, 1H, CH=CH_2), 4.92 (ddt, J = 10.2, 2.3, 1.2 Hz, 1H, CH=CH_2), 3.36 (t, J = 6.7 Hz, 2H, OCH_2), 3.33 (s, 3H, OCH_3), 2.08 – 1.99 (m, 2H, CH_2=CHCH_2), 1.60 – 1.51 (m, 2H, OCH_2CH_2), 1.42 – 1.23 (m, 10H, CH_2). **^{13}C NMR (101 MHz, $CDCl_3$)** δ 139.35 (*C*H=CH_2), 114.25 (CH=*C*H_2), 73.11 (O*C*H_2), 58.68 (O*C*H_3), 33.95, 29.80, 29.59, 29.22, 29.07, 26.28. **HRMS** (ESI): calculated for $C_{11}H_{23}O$ $[M+H]^+$ requires *m*/*z* 171.17434, found *m*/*z* 171.17452.

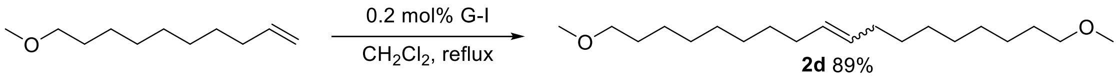

Supplementary Figure 16 Synthesis of **2d**. See below for details.

From **S4** (5.073 g, 29.79 mmol) and Grubbs 1st generation catalyst (49 mg, 0.060 mmol), in 30 mL of CH_2Cl_2, following the general procedure, a 4.1:1 *E*:*Z* mixture of alkene **2d** was obtained. Chromatographic purification (3-10% Et_2O/petrol) gave a 4.1:1 *E*:*Z* mixture of alkene **2d** (4.133 g, 13.22 mmol, 89%), as a colorless oil.

Data for **2d**: **^{1}H NMR (400 MHz, $CDCl_3$)** δ 5.42 – 5.30 (m, 2H, C*H*=C*H*), 3.36 (t, J = 6.7 Hz, 4H, OCH_2), 3.32 (s, 6H, CH_3), 2.05 – 1.88 (m, 4H, CH=CHCH_2), 1.60 – 1.50 (m, 4H, OCH_2CH_2), 1.40 – 1.20 (m, 20H, CH_2). **^{13}C NMR (101 MHz, $CDCl_3$)** δ 130.48 (*C*H=*C*H *E* isom), 130.02 (*C*H=*C*H *Z* isom), 73.12 (O*C*H_2), 58.68 (O*C*H_3), 32.74, 29.90, 29.81, 29.78, 29.62, 29.60, 29.40, 29.25, 27.35, 26.29. **HRMS** (ESI): calculated for $C_{20}H_{41}O_2$ $[M+H]^+$ requires *m*/*z* 313.3101, found *m*/*z* 313.3098.

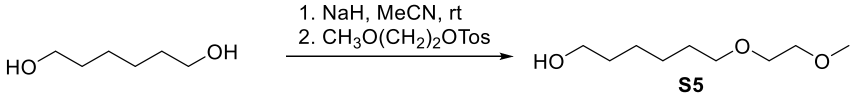

Supplementary Figure 17 Synthesis of **S5**. See below for details.

Hexanediol (4.727 g, 40 mmol) was added to dry MeCN (100 ml). NaH (60% in mineral oil, 800 mg, 20 mmol) was added and the mixture was stirred for 2 h at rt. Ethylene glycol monomethyl ether tosylate (4.606 g, 20 mmol) was then added and the reaction was heated at reflux overnight. The mixture was then concentrated under vacuum and the residue partitioned in water and petrol. Organic phase was further extracted with water, combined aqueous phases were saturated with NaCl and extracted with EtOAc. The organic phases were dried with anhydrous Na_2SO_4, decanted, and concentrated under vacuum. The product was purified by silica chromatography to provide **S5** (2.067 g, 11.73 mmol, 59%) as a yellowish oil.

^{1}H NMR (400 MHz, CD_3OD) δ 3.63 (t, J = 6.6 Hz, 2H, OC*H*$_2$), 3.57 (ddd, J = 5.7, 3.1, 1.3 Hz, 2H, OC*H*$_2$), 3.53 (ddd, J = 5.9, 3.1, 1.3 Hz, 2H, OC*H*$_2$), 3.46 (t, J = 6.7 Hz, 2H, OC*H*$_2$), 3.38 (s, 3H, C*H*$_3$), 1.66 – 1.52 (m, 4H, C*H*$_2$), 1.43 – 1.32 (m, 4H, C*H*$_2$). **^{13}C NMR (101 MHz, CD_3OD)** δ 72.14 (O*C*H$_2$), 71.56 (O*C*H$_2$), 70.13 (O*C*H$_2$), 63.04 (O*C*H$_2$), 59.21 (*C*H$_3$), 32.83 (*C*H$_2$), 29.66 (*C*H$_2$), 26.03 (*C*H$_2$), 25.68 (*C*H$_2$).

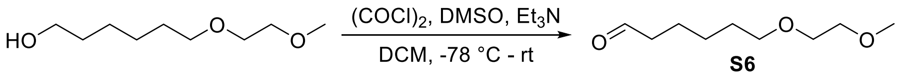

Supplementary Figure 18 Synthesis of **S6**. See below for details.

Oxalyl chloride (1.405 ml, 16.61 mmol) was added to dry CH_2Cl_2 (25 ml) and the solution cooled to -78 °C. DMSO (2.359 ml, 33.22 mmol) in dry DCM (5 ml) was then added slowly, and the reaction stirred for 5 min. Then compound **S5** (2.927 g) in dry CH_2Cl_2 (5 ml) was slowly added. After 15 min Et_3N (11.57 ml, 83 mmol) was slowly added, the reaction was stirred at -78 °C for 10 min and allowed to warm up to rt over 1 h. It was then combined with water and stirred for 10 min. Phases were separated and the organic phase was washed with citric acid solution, sat. aq. $NaHCO_3$, and brine. It was then dried with anhydrous Na_2SO_4, decanted, and concentrated under vacuum. The product was purified by silica chromatography (1.5-3% MeOH/CH_2Cl_2) to provide **S6** (2.400 g, 13.77 mmol, 83%) as a yellowish oil.

^{1}H NMR (400 MHz, CD_3OD) δ 9.75 (t, J = 1.8 Hz, 1H, C*H*O), 3.56 (ddd, J = 5.8, 3.1, 1.3 Hz, 2H, OC*H*$_2$), 3.52 (ddd, J = 5.8, 3.1, 1.3 Hz, 2H, OC*H*$_2$), 3.45 (t, J = 6.6 Hz, 2H, OC*H*$_2$), 3.37 (s, 3H, C*H*$_3$), 2.43 (td, J = 7.4, 1.8 Hz, 2H, C*H*$_2$CHO), 1.71 – 1.53 (m, 4H, C*H*$_2$), 1.45 – 1.32 (m, 2H, C*H*$_2$). **^{13}C NMR (101 MHz, CD_3OD)** δ 202.78 (*C*HO), 72.10 (O*C*H$_2$), 71.26 (O*C*H$_2$), 70.18 (O*C*H$_2$), 59.20 (*C*H$_3$), 43.96 (*C*H$_2$CHO), 29.48 (*C*H$_2$), 25.86 (*C*H$_2$), 22.03 (*C*H$_2$). **HRMS** (ESI): calculated for $C_9H_{19}O_3$ $[M+H]^+$ requires *m*/*z* 175.13287, found *m*/*z* 175.13297.

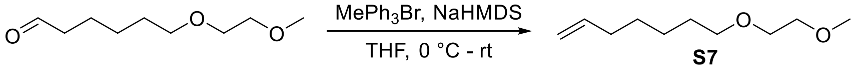

Supplementary Figure 19 Synthesis of **S7**. See below for details.

$MePPh_3Br$ (7.381 g, 20.66 mmol) was added to dry THF (140 ml) under Ar atmosphere and the mixture cooled to 0 °C. NaHMDS (2M, 10.33 ml, 20.66 mmol) was then added slowly and the mixture was stirred for 30 min at 0 °C. Compound **S6** (2.400 g, 13.77 mmol) was then added in a small amount of dry THF. The reaction was stirred at rt overnight. The mixture was then partitioned in EtOAc and sat. aq. NH_4Cl, and the aqueous phase further extracted with EtOAc. Combined organic phases were dried with anhydrous Na_2SO_4, decanted, and concentrated under vacuum. Crude product was purified by a short silica column (5-10% EtOAc/petrol) to provide **S7** (2.301 g, 13.36 mmol, 97%) as a colourless oil.

^{1}H NMR (400 MHz, CD_3OD) δ 5.80 (ddt, J = 16.9, 10.2, 6.7 Hz, 1H, C*H*=CH_2), 4.98 (ddt, J = 17.1, 2.2, 1.6 Hz, 1H, CH=CH_2), 4.92 (ddt, J = 10.2, 2.3, 1.2 Hz, 1H, CH=CH_2), 3.59 – 3.51 (m, 4H, OCH_2), 3.45 (t, J = 6.8 Hz, 2H, OCH_2), 3.38 (s, 3H, OCH_3), 2.04 (qd, J = 6.7, 3.4 Hz, 2H, CH_2=CHCH_2), 1.68 – 1.53 (m, 2H, OCH_2CH_2), 1.46 – 1.29 (m, 4H, CH_2). **^{13}C NMR (101 MHz, CD_3OD)** δ 139.11 (*C*H=CH_2), 114.41 (CH=*C*H_2), 72.14, 71.64, 70.13, 59.22 (*C*H_3), 33.85 (CH_2=CH*C*H_2), 29.58, 28.89, 25.72. **HRMS** (ESI): calculated for $C_{10}H_{21}O_2$ $[M+H]^+$ requires *m*/*z* 173.15361, found *m*/*z* 173.15393.

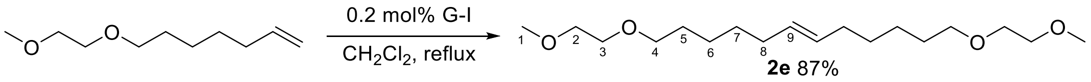

Supplementary Figure 20 Synthesis of **2e**. See below for details.

From **S7** (2.307 g, 13.39 mmol) and Grubbs 2nd generation catalyst (23 mg, 0.067 mmol), in 15 mL of CH_2Cl_2, following the general procedure, a 4.6:1 *E*:*Z* mixture of alkene **2e** was obtained. Chromatographic purification (1% MeOH/DCM) gave a 4.6:1 *E*:*Z* mixture of alkene **2e** (1.840 g, 5.81 mmol, 87%), as a yellowish oil.

^{1}H NMR (400 MHz, CD_3OD) δ 5.43 – 5.30 (m, 2H, 9-H), 3.59 – 3.55 (m, 4H, 2-H/3-H), 3.55 – 3.51 (m, 4H, 2-H/3-H), 3.47 – 3.42 (m, 4H, 4-H), 3.38 (s, 6H, 1-H), 2.05 – 1.92 (m, 4H, 8-H), 1.65 – 1.53 (m, 4H, 5-H), 1.41 – 1.27 (m, 8H, 6-H, 7-H). **^{13}C NMR (101 MHz, CD_3OD)** δ 130.41 (C9), 72.15, 71.70, 70.12, 59.21 (C1), 32.64, 29.60, 25.74. **HRMS** (ESI): calculated for $C_{18}H_{37}O_4$ $[M+H]^+$ requires *m*/*z* 317.26864, found *m*/*z* 317.26827.

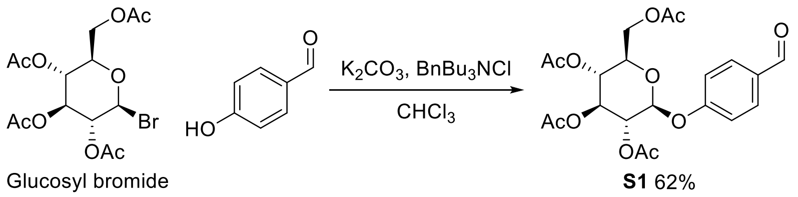

Supplementary Figure 21 Synthesis of **S1**. See below for details.

4-Hydroxybenzaldehyde (1.222 g, 10 mmol) and 2,3,4,6-tetra-*O*-acetyl-α-D-glucopyranosyl bromide (4.934 g, 12 mmol) were added to $CHCl_3$ (20 ml). K_2CO_3 (6.911 g, 50 mmol) and $BnBu_3NCl$ (456 mg, 2 mmol) were then added and the reaction was stirred overnight at 35 °C. It was then carefully neutralised with 1M HCl solution. The organic phase was separated and washed with sat. aq. $NaHCO_3$ and brine. It was then dried with anhydrous Na_2SO_4, decanted, and concentrated under vacuum. Crude product was purified by silica chromatography (30-50% EtOAc/pentane) to provide **S1** (2.820 g, 6.23 mmol, 62%) as a white solid. Spectroscopic properties matched those previously reported.[4]

Data for **S1**: $\boldsymbol{R_f}$ 0.25 (50% EtOAc – pentane). **m.p.** 140.5 °C (10% pentane – Et_2O). $[\alpha]^{25}_{D}$ = – 28.0 (*c* = 1.50, $CHCl_3$). **^{1}H NMR (500 MHz, $CDCl_3$)** δ 9.92 (1 H, s, CHO), 7.85 (2 H, d, *J* = 8.7 Hz, Ar), 7.10 (2 H, d, *J* = 8.7 Hz, Ar), 5.28-5.34 (2 H, m, 2-H and 3-H), 5.21 (1 H, d, *J* = 7.3 Hz, 1-H), 5.18 (1 H, t, *J* = 9.7 Hz, 4-H), 4.28 (1 H, dd, *J* = 12.3 and 5.5 Hz, 6-H_A), 4.18 (1 H, dd, *J* = 12.3 and 2.4 Hz, 6-H_B), 3.92 (1 H, ddd, *J* = 10.1, 5.5 and 2.4 Hz, 5-H), 2.07 (3 H, s, Me OAc), 2.06 (3 H, s, Me OAc), 2.05 (3 H, s, Me OAc), 2.04 (3 H, s, Me OAc). **^{13}C NMR (125 MHz, $CDCl_3$)** δ 190.8 (CHO), 170.6 (C=O Ac), 170.3 (C=O Ac), 169.5 (C=O Ac), 169.3 (C=O Ac), 161.4 (C Ar), 132.0 (C Ar), 131.9 (2 × CH Ar), 116.9 (2 × CH Ar), 98.2 (C-1), 72.7 (C-2), 72.5 (C-5), 71.1 (C-3), 68.3 (C-4), 62.0 (C-6), 20.8 (Me Ac), 20.75 (Me Ac), 20.74 (Me Ac), 20.72 (Me Ac). **HRMS** (ESI): calculated for $C_{21}H_{24}O_{11}Na$ $[M+Na]^+$ requires *m*/*z* 475.1211, found *m*/*z* 475.1210.

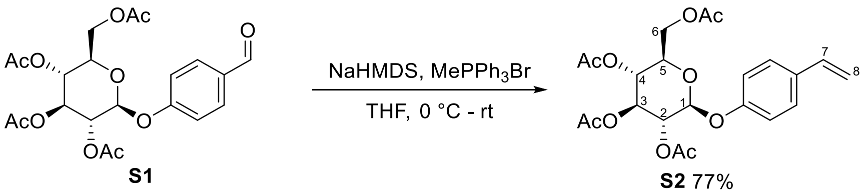

Supplementary Figure 22 Synthesis of **S2**. See below for details.

To a cold (0 ºC) suspension of $MePPh_3Br$ (4.9 g, 13.6 mmol) in dry THF, NaHMDS 1.0 M solution in THF (13.6 mL, 13.6 mmol) was added. The suspension turned into a bright yellow solution and was stirred at that temperature for 30 min. A solution of aldehyde **S1** (4.1 g, 9.06 mmol) in 90 mL of dry THF was added and the reaction was warmed to rt and stirred for 12h until completion. The reaction mixture was quenched with saturated NH_4Cl solution (50 mL) and extracted with EtOAc (3 x 100 mL). The combined organic layers were dried using Na_2SO_4, filtered, and the solvent was evaporated under reduced pressure. Chromatographic purification (gradient elution: 10:90 → 60:40 EtOAc – pentane) gave **S2** (3.1 g, 77%), as a white solid. Spectroscopic properties matched those previously reported.[5]

Data for **S2**: $\boldsymbol{R_f}$ 0.40 (50% EtOAc – pentane). **m.p.** 112.0 °C (10% pentane – Et_2O). $[\alpha]^{25}_D$ = – 22.5 (*c* = 1.20, $CHCl_3$). **^{1}H NMR (500 MHz, $CDCl_3$)** δ 7.34 (2 H, d, *J* = 8.6 Hz, Ar), 6.95 (2 H, d, *J* = 8.8 Hz, Ar), 6.66 (1 H, dd, *J* = 17.6 and 10.9 Hz, 7-H), 5.64 (1 H, dd, *J* = 17.7 and 0.9 Hz, 8-H_{trans}), 5.24-5.32 (2 H, m, 2-H and 3-H), 5.19 (1 H, d, *J* = 10.7 Hz, 8-H_{cis}), 5.17 (1 H, t, *J* = 9.6 Hz, 4-H), 5.08 (1 H, d, *J* = 7.5 Hz, 1-H), 4.29 (1 H, dd, *J* = 12.3 and 5.3 Hz, 6-H_A), 4.17 (1 H, dd, *J* = 12.3 and 2.5 Hz, 6-H_B), 3.86 (1 H, ddd, *J* = 10.1, 5.3 and 2.5 Hz, 5-H), 2.08 (3 H, s, Me OAc), 2.06 (3 H, s, Me OAc), 2.05 (3 H, s, Me OAc), 2.04 (3 H, s, Me OAc). **^{13}C NMR (125 MHz, $CDCl_3$)** δ 170.7 (C=O Ac), 170.4 (C=O Ac), 169.5 (C=O Ac), 169.4 (C=O Ac), 156.6 (C Ar), 136.0 (C-7), 133.2 (C Ar), 127.5 (2 × CH Ar), 117.2 (2 × CH Ar), 113.2 (C-8), 99.3 (C-1), 72.9 (C-2), 72.2 (C-5), 71.3 (C-3), 68.4 (C-4), 62.1 (C-6), 20.9 (Me Ac), 20.80 (Me Ac), 20.78 (Me Ac), 20.75 (Me Ac). **HRMS** (ESI): calculated for $C_{22}H_{26}O_{10}Na$ $[M+Na]^+$ requires *m*/*z* 473.1418, found *m*/*z* 473.1413.

General procedure for acetate deprotection: To a solution of acetate in dry MeOH (10.0 mL/mmol), NaOMe (0.1 equiv) was added in one portion at room temperature. The reaction was monitored by TLC until completion, and quenched with Amberlyst-15, to reach a neutral pH. The mixture was filtered and the solvent was evaporated under reduced pressure to give the corresponding free carbohydrate, which was purified by chromatography on silica gel using the appropriate mixture of eluents.

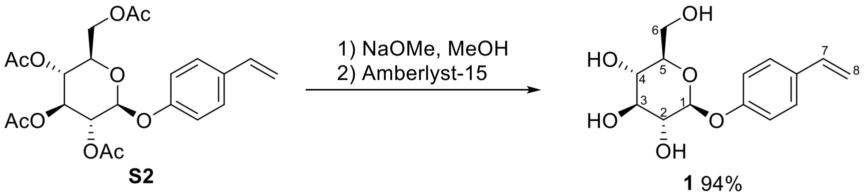

Supplementary Figure 23 Synthesis of **1**. See below for details.

From alkene **S2** (2.0 g, 4.4 mmol) and NaOMe (47 mg, 0.80 mmol), in 20.0 mL of MeOH, following the general procedure, alkene **1** was obtained. Chromatographic purification (gradient elution: 5:95 → 25:75 MeOH – CH_2Cl_2) gave **1** (1.24 g, 94%), as a white solid. Spectroscopic properties matched those previously reported.[5]

Data for **1**: ***R**f* 0.30 (20% MeOH – CH_2Cl_2). **m.p.** 187.0 °C (10% Et_2O – CH_2Cl_2). **$[\alpha]^{25}_D$** = – 57.5 (*c* = 2.01, MeOH). **^{1}H NMR (500 MHz, CD_3OD)** δ 7.36 (2 H, d, *J* = 8.7 Hz, Ar), 7.05 (2 H, d, *J* = 8.7 Hz, Ar), 6.67 (1 H, dd, *J* = 17.6 and 10.9 Hz, 7-H), 5.64 (1 H, dd, *J* = 17.6 and 1.0 Hz, 8-H_{trans}), 5.12 (1 H, dd, *J* = 10.9 and 1.0 Hz, 8-H_{cis}), 4.91 (1 H, d, *J* = 7.3 Hz, 1-H), 3.90 (1 H, dd, *J* = 12.1 and 2.2 Hz, 6-H_A), 3.70 (1 H, dd, *J* = 12.0 and 5.5 Hz, 6-H_B), 3.37-3.48 (4 H, m, 2-H, 3-H, 4-H and 5-H). **^{13}C NMR (125 MHz, CD_3OD)** δ 158.8 (C Ar), 137.5 (C-7), 133.4 (C Ar), 128.3 (2 × CH Ar), 117.7 (2 × CH Ar), 112.4 (C-8), 102.2 (C-1), 78.1 (C-2), 78.0 (C-5), 74.9 (C-3), 71.4 (C-4), 62.5 (C-6). **HRMS** (ESI): calculated for $C_{14}H_{18}O_6Na$ $[M+Na]^+$ requires *m*/*z* 305.0996, found *m*/*z* 305.0997.

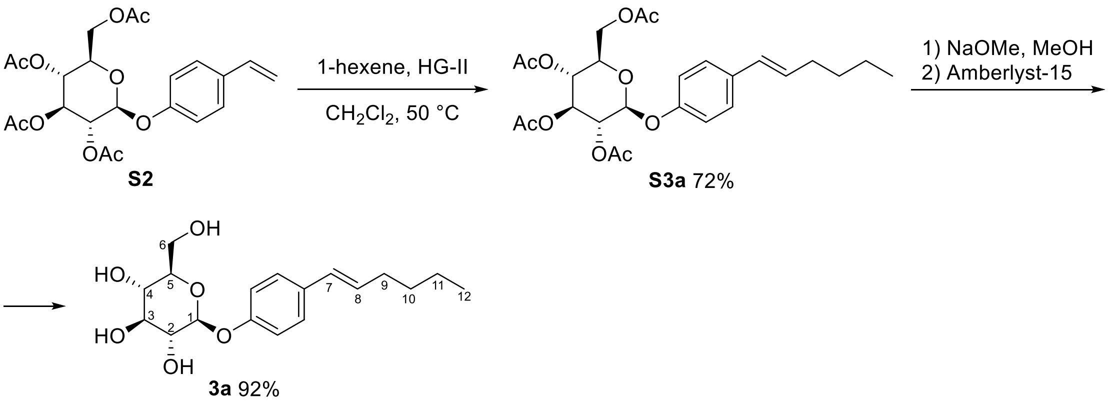

Supplementary Figure 24 Synthesis of **3a**. See below for details.

From alkene **S2** (300 mg, 0.67 mmol), 1-Hexene (0.4 mL, 3.33 mmol) and Hoveyda-Grubbs 2nd generation catalyst (20 mg, 0.03 mmol), in 7 mL of CH_2Cl_2, following the general procedure, alkene **S3a** was obtained. Chromatographic purification (gradient elution: 10:90 → 50:50 EtOAc – pentane) gave **S3a** (245 mg, 72%), as a white solid.

From alkene **S3a** (245 mg, 0.47 mmol) and NaOMe (7 mg, 0.14 mmol), in 5.0 mL of MeOH, following the general procedure, alkene **3a** was obtained. Chromatographic purification (gradient elution: 10:90 → 50:50 MeOH – CH_2Cl_2) gave **3a** (147 mg, 92%), as a white solid. Spectroscopic properties matched those previously reported.[5]

Data for **3a**: ***R**f* 0.35 (20% MeOH – CH_2Cl_2). **m.p.** 127.0 °C (10% Et_2O – CH_2Cl_2). **$[\alpha]^{25}_D$** = – 49.4 (*c* = 1.60, MeOH). **^{1}H NMR (500 MHz, CD_3OD)** δ 7.27 (2 H, d, *J* = 8.7 Hz, Ar), 7.02 (2 H, d, *J* = 8.7 Hz, Ar), 6.32 (1 H, d, *J* = 15.8 Hz, 7-H), 6.12 (1 H, dt, *J* = 15.7 and 6.9 Hz, 8-H), 4.88 (1 H, d, *J* = 7.3 Hz, 1-H), 3.89 (1 H, dd, *J* = 12.0 and 2.2 Hz, 6-H_A), 3.70 (1 H, dd, *J* = 12.1 and 5.3 Hz, 6-H_B), 3.37-3.47 (4 H, m, 2-H and 3-H and 4-H and 5-H), 2.14-2.22 (2 H, m, 9-H_2), 1.42-1.53 (2 H, m, 10-H_2), 1.33-1.42 (2 H, m, 11-H_2), 0.94 (3 H, t, *J* = 7.2 Hz, 12-H_3). **^{13}C NMR (125 MHz, CD_3OD)** δ 158.1 (C Ar), 133.8 (C Ar), 130.4 (C-7), 130.3 (C-8), 127.9 (2 × CH Ar), 117.7 (2 × CH Ar), 102.3 (C-1), 78.1 (C-2), 78.0 (C-5), 74.9 (C-3), 71.4 (C-4), 62.5 (C-6), 33.8 (C-9), 32.9 (C-10), 23.3 (C-11), 14.3 (C-12). **HRMS** (ESI): calculated for $C_{18}H_{26}O_6Na$ $[M+Na]^+$ requires *m*/*z* 361.1622, found *m*/*z* 361.1622.

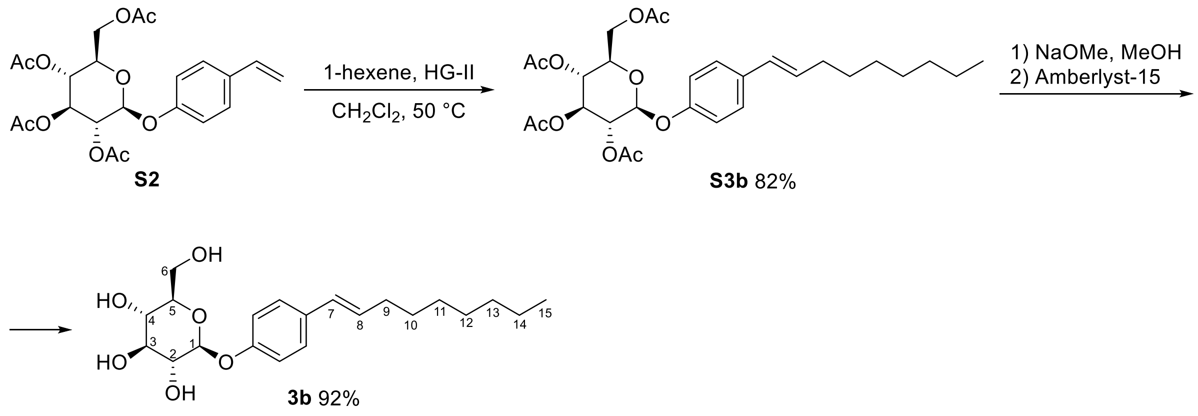

Supplementary Figure 25 Synthesis of **3b**. See below for details.

From alkene **S2** (300 mg, 0.67 mmol), 1-Nonene (0.58 mL, 3.35 mmol) and Hoveyda-Grubbs 2nd generation catalyst (20 mg, 0.03 mmol), in 7 mL of CH_2Cl_2, following the general procedure, alkene **S3b** was obtained. Chromatographic purification (gradient elution: 10:90 → 50:50 EtOAc – pentane) gave **S3b** (300 mg, 82%), as a white solid.

From alkene **S3a** (300 mg, 0.67 mmol) and NaOMe (7 mg, 0.14 mmol), in 5.0 mL of MeOH, following the general procedure, alkene **3b** was obtained. Chromatographic purification (gradient elution: 10:90 → 50:50 MeOH – CH_2Cl_2) gave **3b** (229 mg, 92%), as a white solid.

Data for **3b**: $\boldsymbol{R_f}$ 0.40 (20% MeOH – CH_2Cl_2). **m.p.** 120.0 °C (10% MeOH – CH_2Cl_2). $[\alpha]^{25}_{D}$ = – 40.4 (*c* = 2.40, H_2O). **^{1}H NMR (500 MHz, CD_3OD)** δ 7.27 (2 H, d, *J* = 8.6 Hz, Ar), 7.02 (2 H, d, *J* = 8.7 Hz, Ar), 6.32 (1 H, d, *J* = 15.8 Hz, 7-H), 6.12 (1 H, dt, *J* = 15.8 and 7.0 Hz, 8-H), 4.88 (1 H, d, *J* = 7.4 Hz, 1-H), 3.89 (1 H, dd, *J* = 12.1 and 2.3 Hz, 6-H_A), 3.70 (1 H, dd, *J* = 12.1 and 5.5 Hz, 6-H_B), 3.36-3.48 (4 H, m, 2-H and 3-H and 4-H and 5-H), 2.18 (2 H, qd, *J* = 7.1 and 1.5 Hz, 9-H_2), 1.42-1.51 (2 H, m, 10-H_2), 1.25-1.40 (8 H, m, 11-H_2 and 12-H_2 and 13-H_2 and 14-H_2), 0.90 (3 H, t, *J* = 7.1 Hz, 15-H_3). **^{13}C NMR (125 MHz, CD_3OD)** δ 156.7 (C Ar), 132.4 (C Ar), 129.02 (C-7), 128.95 (C-8), 126.5 (2 × CH Ar), 116.4 (2 × CH Ar), 101.0 (C-1), 76.8 (C-2), 76.6 (C-5), 73.5 (C-3), 70.0 (C-4), 61.1 (C-6), 32.7 (C-9), 31.6 (C-10), 29.3 (C-11), 28.9 (2C, C-12 and C-13), 22.3 (C-14), 13.0 (C-15). **HRMS** (ESI): calculated for $C_{21}H_{32}O_6Na$ $[M+Na]^+$ requires *m*/*z* 403.2097, found *m*/*z* 403.2091.

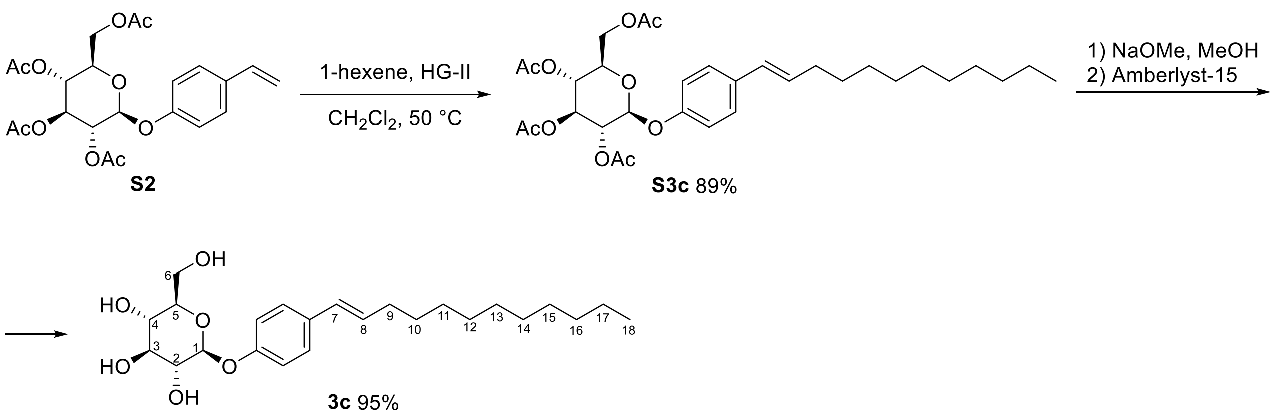

Supplementary Figure 26 Synthesis of **3c**. See below for details.

From alkene **S2** (300 mg, 0.67 mmol), 1-Dodecene (0.74 mL, 3.3 mmol) and Hoveyda-Grubbs 2nd generation catalyst (20 mg, 0.03 mmol), in 7 mL of CH_2Cl_2, following the general procedure, alkene **S3c** was obtained. Chromatographic purification (gradient elution: 10:90 → 50:50 EtOAc – pentane) gave **S3c** (350 mg, 89%), as a white solid.

From alkene **S3c** (280 g, 0.47 mmol) and NaOMe (5 mg, 0.09 mmol), in 5.0 mL of MeOH, following the general procedure, alkene **3c** was obtained. Chromatographic purification (gradient elution: 10:90 → 50:50 MeOH – CH_2Cl_2) gave **3c** (188 mg, 95%), as a white solid. Spectroscopic properties matched those previously reported.[5]

Data for **3c**: ***R**f* 0.40 (20% MeOH – CH_2Cl_2). **m.p.** 117.0 °C (10% Et_2O – CH_2Cl_2). **$[\alpha]^{25}_D$** = – 38.0 (*c* = 5.00, MeOH). **^{1}H NMR (500 MHz, CD_3OD)** δ 7.25 (2 H, d, *J* = 8.7 Hz, Ar), 7.01 (2 H, d, *J* = 8.8 Hz, Ar), 6.31 (1 H, d, *J* = 15.8 Hz, 7-H), 6.11 (1 H, dt, *J* = 15.7 and 6.9 Hz, 8-H), 4.87 (1 H, d, *J* = 7.5 Hz, 1-H), 3.88 (1 H, dd, *J* = 12.1 and 2.1 Hz, 6-H_A), 3.70 (1 H, dd, *J* = 12.0 and 5.1 Hz, 6-H_B), 3.38-3.47 (4 H, m, 2-H and 3-H and 4-H and 5-H), 2.17 (2 H, q, *J* = 7.0 Hz, 9-H_2), 1.45 (2 H, quint, *J* = 7.2 Hz, 10-H_2), 1.23-1.36 (14 H, m, 11-H_2 and 12-H_2 and 13-H_2 and 14-H_2 and 15-H_2 and 16-H_2 and 17-H_2), 0.89 (3 H, t, *J* = 6.9 Hz, 18-H_3). **^{13}C NMR (125 MHz, CD_3OD)** δ 158.1 (C Ar), 133.7 (C Ar), 130.4 (C-7), 130.3 (C-8), 127.9 (2 × CH Ar), 117.7 (2 × CH Ar), 102.3 (C-1), 78.1 (C-2), 77.9 (C-5), 74.9 (C-3), 71.3 (C-4), 62.5 (C-6), 34.1 (C-9), 33.1 and 30.74 and 30.73 and 30.64 and 30.62 and 30.45 and 30.34 and 23.7 (C-10 and C-11 and C-12 and C-13 and C-14 and C-15 and C-16 and C-17), 14.5 (C-18). **HRMS** (ESI): calculated for $C_{24}H_{38}O_6Na$ $[M+Na]^+$ requires *m*/*z* 445.2561, found *m*/*z* 445.2565.

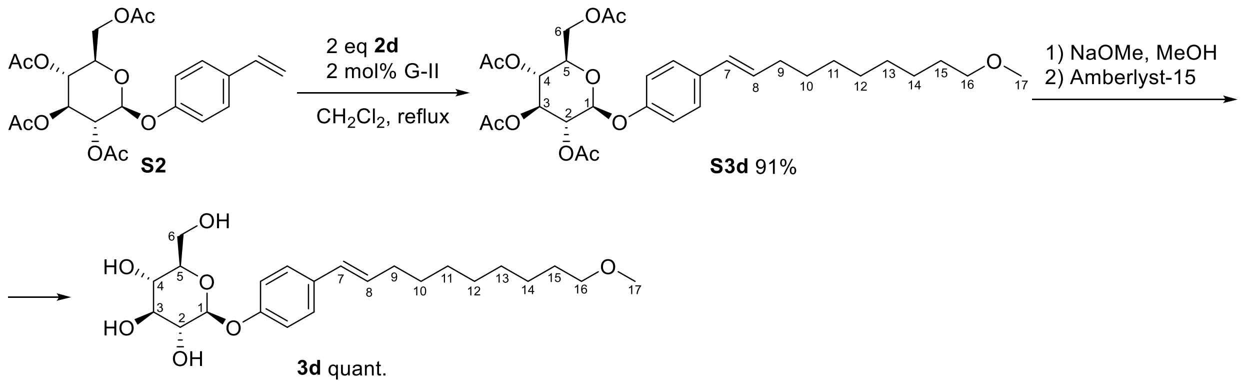

Supplementary Figure 27 Synthesis of **3d**. See below for details.

From alkenes **S2** (225 mg, 0.5 mmol), **2d** (313 mg, 1 mmol), and Grubbs 2nd generation catalyst (8 mg, 0.01 mmol), in 5 mL of CH_2Cl_2, following the general procedure, alkene **S3d** was obtained. Chromatographic purification (gradient elution: 20-40% EtOAc/petrol) gave **S3d** (268 mg, 91%), as an off-white solid.

From alkene **S3d** (195 mg, 0.33 mmol) and NaOMe (4 mg, 0.033 mmol), in 4 mL of MeOH, following the general procedure, alkene **3d** (140 mg, 0.33 mmol, quant.) was obtained.

Data for **3d**: **$[\alpha]^{25}_D$** = – 46.2 (*c* = 1.00, MeOH) **^{1}H NMR (400 MHz, CD_3OD)** δ 7.32 – 7.24 (m, 2H, Ar), 7.07 – 6.98 (m, 2H, Ar), 6.33 (d, *J* = 15.8 Hz, 1H, 7-H), 6.13 (ddd, *J* = 15.8, 7.8, 6.2 Hz, 1H, 8-H), 4.90 – 4.85 (m, 1H, 1-H), 3.90 (dd, *J* = 12.0, 2.1 Hz, 1H, 6-H), 3.71 (dd, *J* = 12.0, 5.2 Hz, 1H, 6-H), 3.52 – 3.34 (m, 6H, 2-H, 3-H, 4-H, 5-H, 16-H), 3.32 (d, *J* = 1.7 Hz, 3H, 17-H), 2.20 (q, *J* = 6.6 Hz, 2H, 9-H), 1.57 (t, *J* = 6.7 Hz, 2H, 15-H), 1.47 (q, *J* = 6.7 Hz, 2H, 10-H), 1.36 (d, *J* = 6.2 Hz, 8H, 11-H, 12-H, 13-H, 14-H). **^{13}C NMR (101 MHz, CD_3OD)** δ 158.11 (C Ar), 133.77 (C Ar), 130.45 (C-7), 130.30 (C-8), 127.89 (CH Ar), 117.75 (CH Ar), 102.35 (C-1), 78.14, 78.00, 74.92, 73.92 (C-16), 71.38, 62.52 (C-6), 58.70 (C-17), 34.05 (C-9), 30.61, 30.57, 30.55, 30.52, 30.24, 27.15. **HRMS** (ESI): calculated for $C_{23}H_{36}O_7Na$ $[M+Na]^+$ requires *m/z* 447.23532, found *m/z* 447.23514.

General procedure for biphasic alkene cross-metathesis: To a flame-dried flask, charged with a catalytic amount of Ru-catalyst, under Ar, at room temperature, was added a solution of hydrophilic alkene in D_2O (previously degassed, bubbling Ar over 30 min.). Hydrophobic alkene was added and the mixture was stirred at the appropriate temperature and stirring speed. The reaction was monitored using Ultra Performance Liquid Chromatography (UPLC) until completion. The solvent was evaporated under reduced pressure to give the corresponding product that was purified by chromatography on silica gel using the appropriate mixture of eluents.

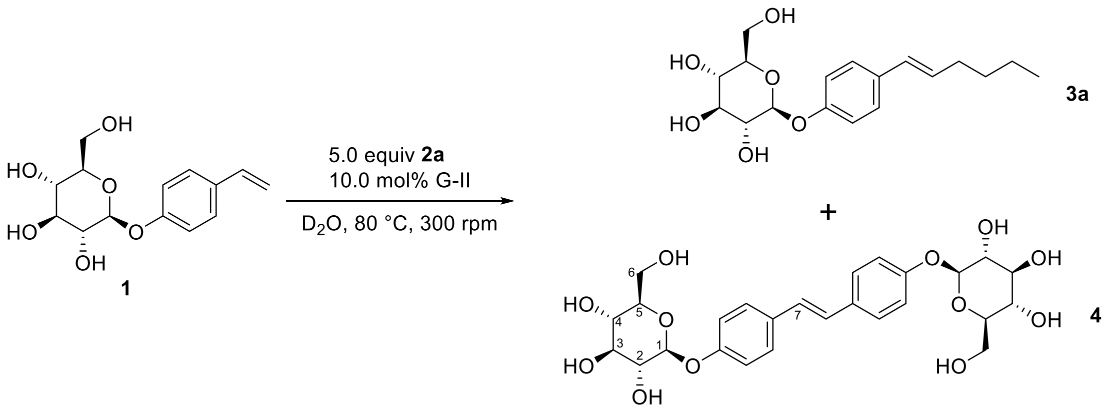

Supplementary Figure 28 Synthesis of **4**. See below for details.

From alkenes **1** (30 mg, 0.106 mmol) and **2a** (0.20 mL, 0.53 mmol) and Grubbs 2nd generation catalyst (10 mg, 0.01 mmol), in 1.5 mL of D_2O (70 mM), following the general procedure at 80 °C and 300 rpm, alkenes **3a** and **4** were obtained. Spectroscopic properties matched those previously reported.[5]

Stopping the reaction after 4h, after chromatographic purification (gradient elution: 10:90 → 50:50 MeOH – CH_2Cl_2) gave **3a** (25 mg, 70%) and **4** (4 mg, 18%), as white solids.

Stopping the reaction after 24h, after chromatographic purification (gradient elution: 10:90 → 50:50 MeOH – CH_2Cl_2) gave **4** (17 mg, 59%), as white solid.

Data for **4**: $\boldsymbol{R_f}$ 0.50 (50% MeOH – CH_2Cl_2). **m.p.** 245.0 °C (10% MeOH – CH_2Cl_2). $\mathbf{[\alpha]^{25}_D}$ = – 71.4 (*c* = 1.40, H_2O). **^{1}H NMR (500 MHz, CD_3OD)** δ 7.46 (4 H, d, *J* = 8.8 Hz, Ar), 7.08 (4 H, d, *J* = 8.8 Hz, Ar), 7.00 (2 H, s, 7-H), 4.92 (2 H, t, *J* = 7.6 Hz, 1-H), 3.91 (2 H, dd, *J* = 12.1 and 2.2 Hz, 6-H_A), 3.71 (2 H, dd, *J* = 12.1 and 5.5 Hz, 6-H_B), 3.44-3.48 (6 H, m, 2-H, 3-H and 5-H), 3.38-3.42 (2 H, m, 4-H). **^{13}C NMR (125 MHz, CD_3OD)** δ 158.5 (2C, 2 × C Ar), 133.5 (2C, 2 × C Ar), 128.4 (4C, 4 × CH Ar), 127.7 (C-7), 117.9 (4C, 4 × CH Ar), 102.3 (C-1), 78.2 (C-5), 78.0 (C-2), 74.9 (C-3), 71.4 (C-4), 62.5 (C-6). **HRMS** (ESI): calculated for $C_{26}H_{32}O_{12}Na$ $[M+Na]^+$ requires *m/z* 559.1786, found *m/z* 559.1786.

Kinetic analysis

Kinetic analyses were performed using a Waters Acquity Ultra Performance Liquid Chromatography (UPLC) H-Class system with Photodiode Array (PDA) detector. Instrument control and data processing were performed using Empower software. Acquity UPLC BEH C18 coloumn, 2.1 x 50 mm with a 1.7 µm size particle was used. A mixture of MeOH:H_2O with a gradient of 5:95 $\rightarrow$ 95:5 over 5 min was used as mobile phase.

Calibration showing linear fitting was obtained for every system. The response factor (Rf) for every alkene was extracted from the linear fitting and has already been reported.[5]

Quantitative results were obtained using a 0.5 mM solution of phloroglucinol in H_2O as standard. The reactions were monitored extracting an aliquot of 20 µL at each time point (usually every 30min or every 1h), diluting to 1.0mL using a 0.5mM solution of standard. We opted to used 214nm wavelength to analyse the data, where all the species, including the standard, have a local maximum of absorbance.

The products of alkene migration caused by Grubbs II catalyst can be observed in reactions done at 80 °C, and when this occurs the products are visible as minor chromatographic peaks in the UPLC traces. In the reactions reported here that form **3a-c**, there was not a great extent of such migration, and in practice the nearest chromatographic peaks due to the migration products were integrated together with the main peaks of **3a-c** in order to account for as much material as possible. On the other hand, significant migration was seen in reaction between **1** and **2e**, which was examined as a hydrophilic alkene to probe the selectivity of surfactant formation. In this case, alkene migration prevented us from isolating a pure sample of the corresponding surfactant **3e**.

Seeding experiment of the system 2c / 3c with 3c

The seeding experiments were performed using alkenes **1** (30 mg, 0.106 mmol) and **2c** (0.20 mL, 0.53 mmol) and Grubbs 2nd generation catalyst (10 mg, 0.01 mmol), in 1.5 mL of D_2O (70 mM), following the general procedure, at 80 °C and 300 rpm, using 20 mol% of amphiphile **3c** present from the beginning of the reaction (Supplementary Figure 29a). Adding 20 mol% of **3c** from the beginning decreases the initial lag-period in both the starting material hydrophilic alkene **1** consumption (Supplementary Figure 29b) and the cross-product **3b** formation (Supplementary Figure 29c).

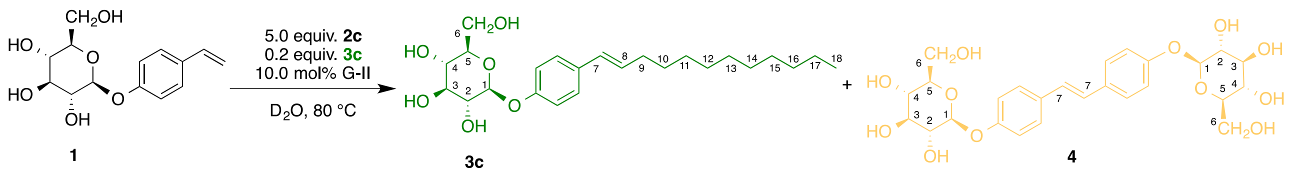

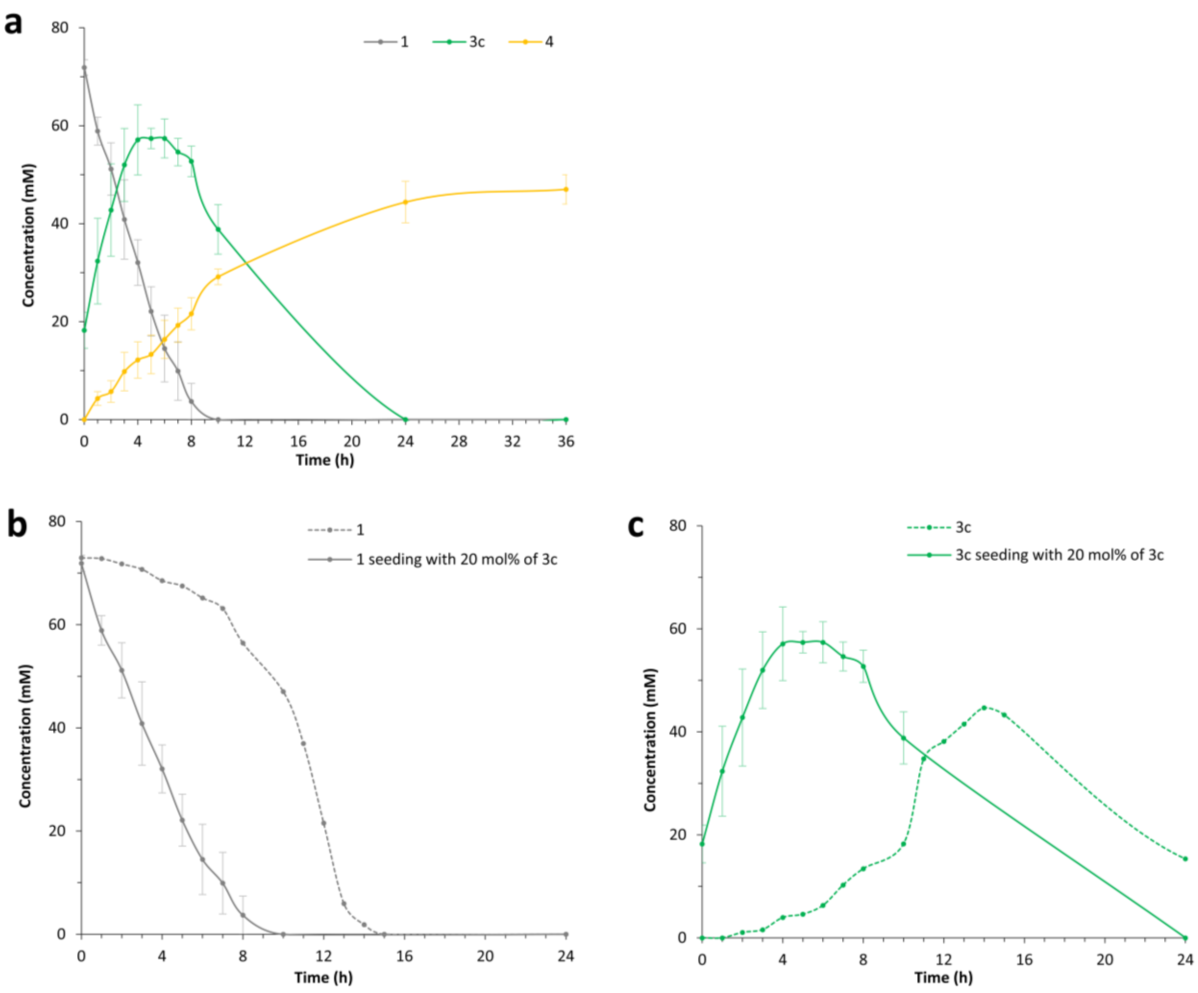

Supplementary Figure 29 Kinetic analysis of autocatalytic **3c** formation. Representing concentration vs. time of **1** (grey), **3c** (green) and **4** (yellow), seeding the reaction with 20 mol% of **3c**. **(a)** Comparison of non-seeded (dashed line) and seeded reaction (straight line), monitoring: **(b)** starting material **1** consumption **(c)** cross-product **3c** formation. Error bars represent standard deviation obtained from three repetitions. Source data are provided as a Source Data file.

Seeding experiment of the system 2c / 3c with 3a

The seeding experiments were performed using alkenes **1** (30 mg, 0.106 mmol) and **2c** (0.20 mL, 0.53 mmol) and Grubbs 2nd generation catalyst (10 mg, 0.01 mmol), in 1.5 mL of D_2O (70 mM), following the general procedure, at 80 °C and 300 rpm, using 20 mol% of amphiphile **3a** present from the beginning of the reaction. (Supplementary Figure 30a). Adding 20 mol% of **3a** from the

beginning decreases the initial lag-period in both the starting material hydrophilic alkene **1** consumption (Supplementary Figure 30b) and the cross-product **3c** formation (Supplementary Figure 30c). Over the course of the reaction, surfactant **3a**, used to seed the reaction, also gets consumed as a result of being metastable.

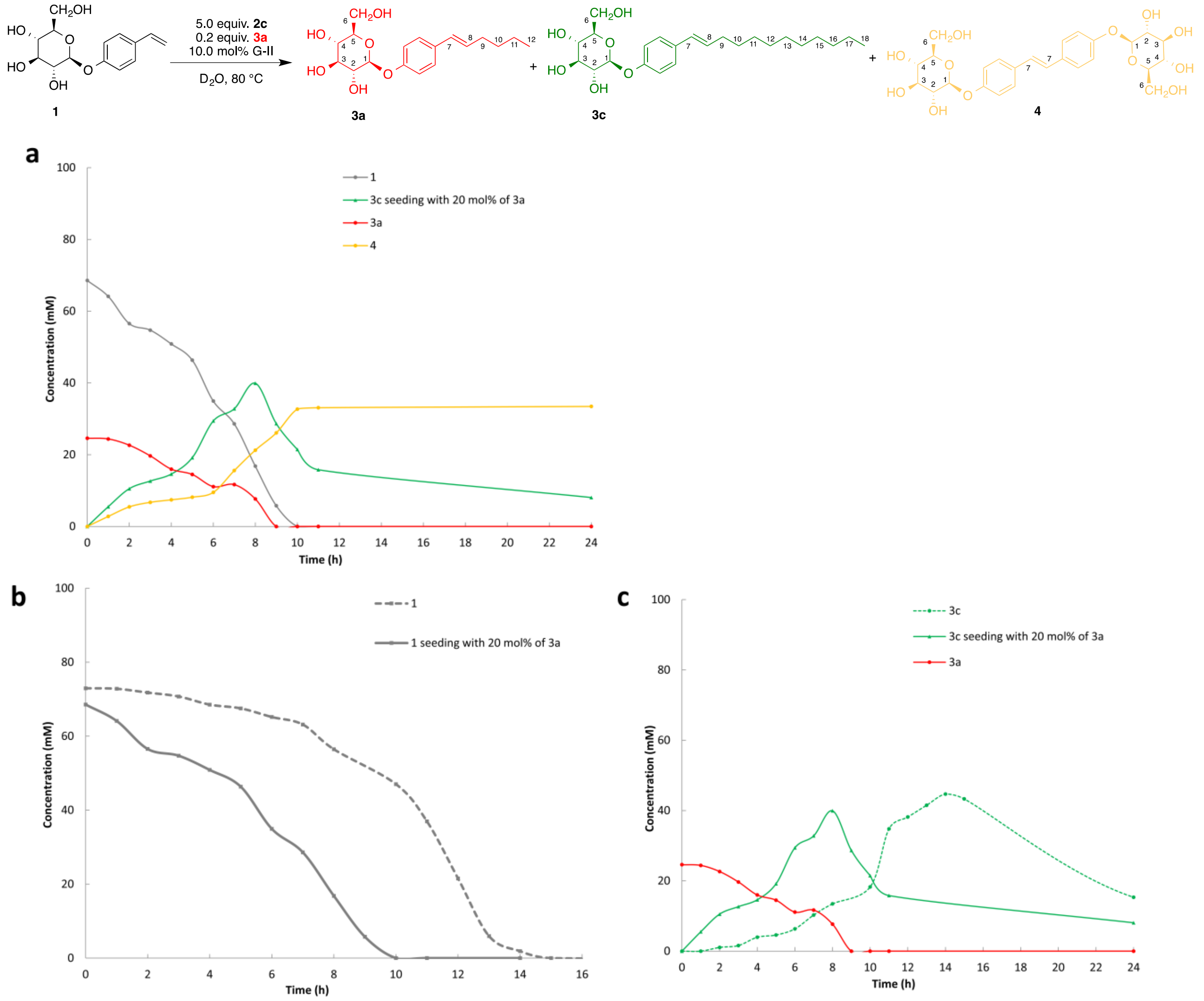

Supplementary Figure 30 Kinetic analysis of cross-catalytic **3c** formation. Showing concentration vs. time of **1** (grey), **3a** (red), **3c** (green) and **4** (yellow), seeding the reaction with 20 mol% of **3a**. **(a)** Comparison of non-seeded (dashed line) and seeded reaction (straight line), monitoring: **(b)** starting material **1** consumption **(c)** cross-product **3c** formation and surfactant **3a** used to seed the reaction. Source data are provided as a Source Data file.

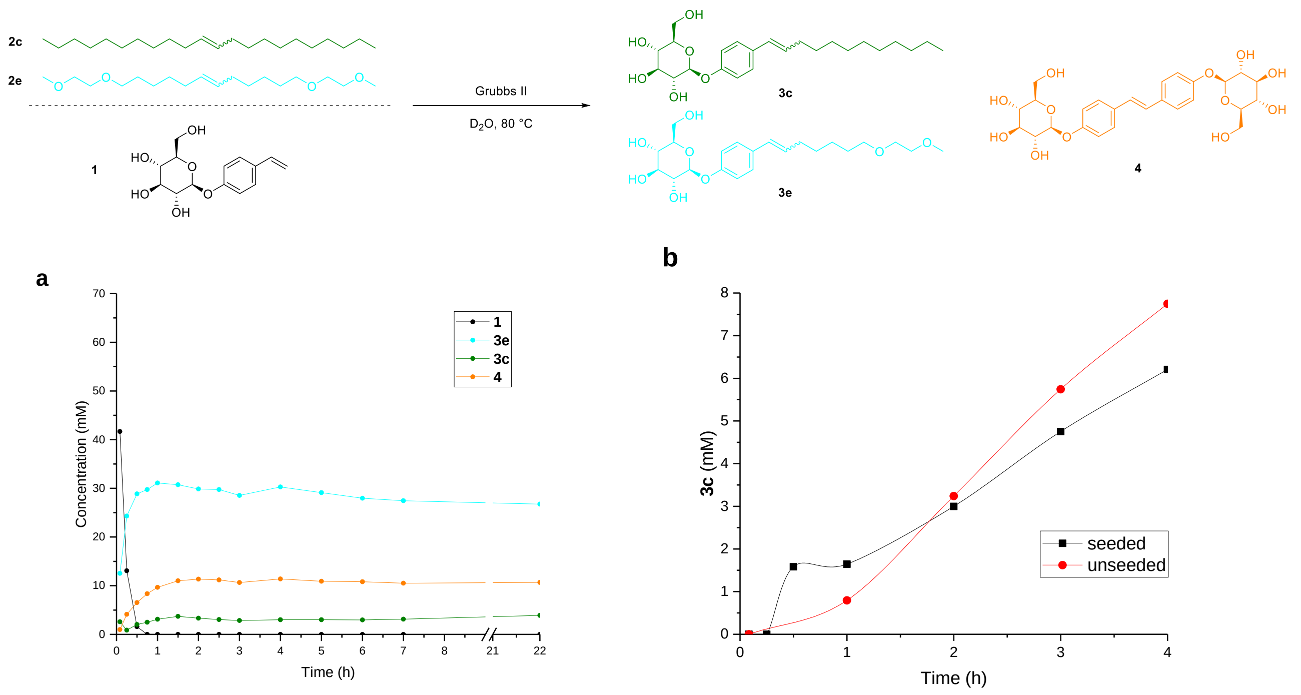

Supplementary Figure 31 Kinetic profiles of reactions including **2e**. **a)** Experiment using an equimolar mixture of **2c** and **2e** as a hydrophobic phase, showing highly selective formation of **3e** followed by very little amphiphile destruction. **b)** No cross-catalysis was observed when a reaction forming **3c** was seeded with 7.5 mol% of **3e**. Source data are provided as a Source Data file.

Continuous stirred tank reactor (CSTR) experiment details

The open system experiments, established in a continuous stirred tank reactor, were set up according to the procedure and scheme below (Supplementary Figure 32). Aqueous inflow syringe was filled with a solution of starting material **1** in degassed D_2O (70 mM for the experiment in Figure 5b, 50 mM for the experiments in Figure 3, or 25 mM for the experiment in Supplementary Figure 33). Where necessary, the syringe was kept at ca. 60 °C using a resistance heater to maintain the solubility of **1**. This syringe was placed in a syringe pump assembly. Organic inflow syringe was filled with a mixture of starting material **2** and Grubbs II catalyst (3.75 wt% for experiments in Figure 3 and Supplementary Figure 33 or 2.66 wt% for the experiment in Figure 5). To solubilise Grubbs II a small amount of DCM was added which was then removed using N_2 flow and vacuum. This syringe was placed in a second syringe pump assembly. Finally, a third empty syringe was set up in a third syringe pump assembly to continuously withdraw the reaction mixture.

For experiments in Figure 3 and Supplementary Figure 33, all syringes were placed in a flask charged with a catalytic amount of Ru-catalyst (10 mg, 0.01 mmol), under Ar. To the flask was added a solution of hydrophilic alkene (15 mg, 0.053 mmol) in 3.0 ml of degassed D_2O. Hydrophobic alkene **2** (0.1 mL, 0.27 mmol) was added directly to the flask and the mixture was stirred at 80 °C and 900 rpm using a magnetic follower.

For experiment in Figure 5b a simplified protocol was followed where the initial reaction mixture was composed by adding to the reaction flask 1.6 ml of solution from the aqueous inflow syringe and 0.4 ml from the organic inflow syringe, to a total volume of 2 ml.

At this point the syringe pumps were switched on, with flow rates adjusted so that influx and efflux were balanced, including a consideration of material withdrawn from the reaction for analysis.

For the experiments in Figure 3 and Supplementary Figure 33 the aqueous inflow was set to 1.0 ml/h, organic inflow to 0.3 ml/h and outflow to 1.3 ml/h including sampling for analysis, to give a space velocity of 0.43 h^{-1}.

For the experiment in Figure 5b, aqueous inflow was 1.2 ml/h, organic inflow 0.3 ml/h, and outflow 1.5 ml/h (at a total volume of 2 ml), providing a space velocity of 0.75 h^{-1}. In this case using a lower space velocity (0.38 h^{-1} was also attempted) led to steady-state concentrations of surfactants that were very low and took a long time to reach. Selectivity was however similar as observed at 0.75 h^{-1} and in batch experiments.

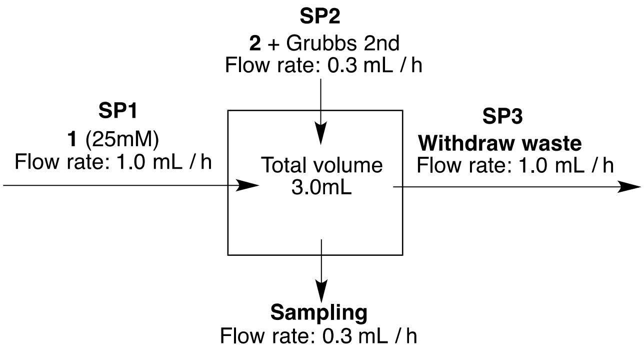

Supplementary Figure 32 Scheme of the continuous stirred tank reactor setup.

We first tested this approach using hydrophobic alkene **2a** in a non-competing scenario. This allows the system to be maintained at a non-equilibrium steady state, with the rate of formation and destruction of replicator **3a** neatly balanced. After 8h under flow parameters, turning off the flows results in movement towards the equilibrium state, consumption of remaining self-reproducing amphiphile **3a** and formation of the thermodynamic product **4** (Supplementary Figure 33).

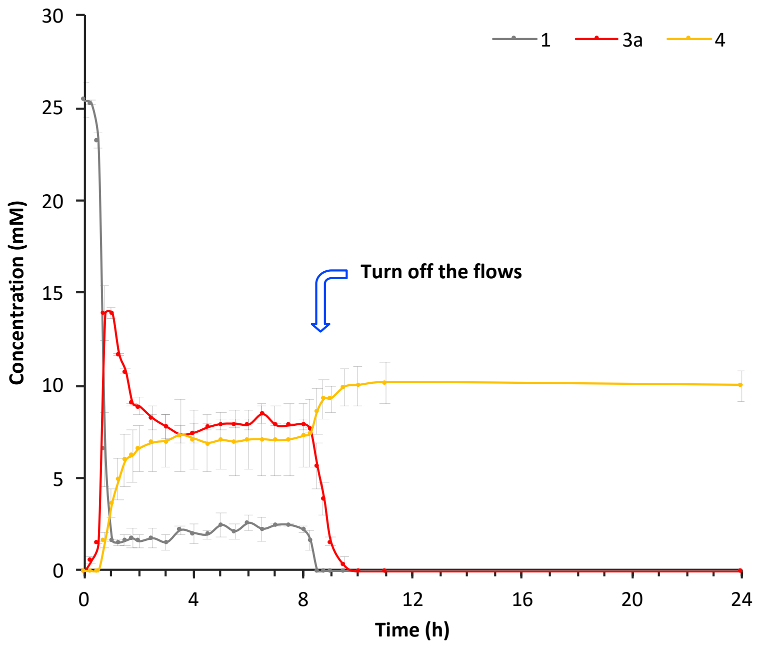

Supplementary Figure 33 Kinetic profile of CSTR experiment. Representing concentration vs. time of **1** (grey), **3a** (red), and **4** (yellow), using a continuous stirred tank reactor (CSTR) to keep the system in a non-equilibrium steady state. Error bars represent standard deviation obtained from three repetitions. Source data are provided as a Source Data file.

Kinetic study of replicator destruction

The graph presented in Figure 4b of main text is obtained by overlapping the results of three different experiments, independently studying the destruction of all the replicators. Subjecting surfactant replicators **3a** (Supplementary Figure 34a), **3b** (Supplementary Figure 34b) or **3c** (Supplementary Figure 34c) to the standard conditions of the competing reaction (Grubbs 2nd generation catalyst and equal amounts of hydrophobic alkenes **2a**, **2b** and **2c** in D_2O).

Experiment 1 (Supplementary Figure 34a): Replicator **3a** gets destroyed to initially form complementary surfactant replicators **3b** and **3c** and thermodynamic product **4**. The mixture of replicators is finally consumed to form thermodynamic product **4** as the only remaining species.

Experiment 2 (Supplementary Figure 34b): Similar as before, replicator **3b** is destroyed to form a mixture of surfactant replicators **3a**, **3b** and **3c** and thermodynamic product **4**. Finally thermodynamic product **4** is the only remaining species.

Experiment 3 (Supplementary Figure 34c): Replicator **3c** gets destroyed to initially form complementary surfactant replicators **3a** and **3b** and thermodynamic product **4**. The mixture of replicators is finally consumed to form thermodynamic product **4** as the only remaining species.

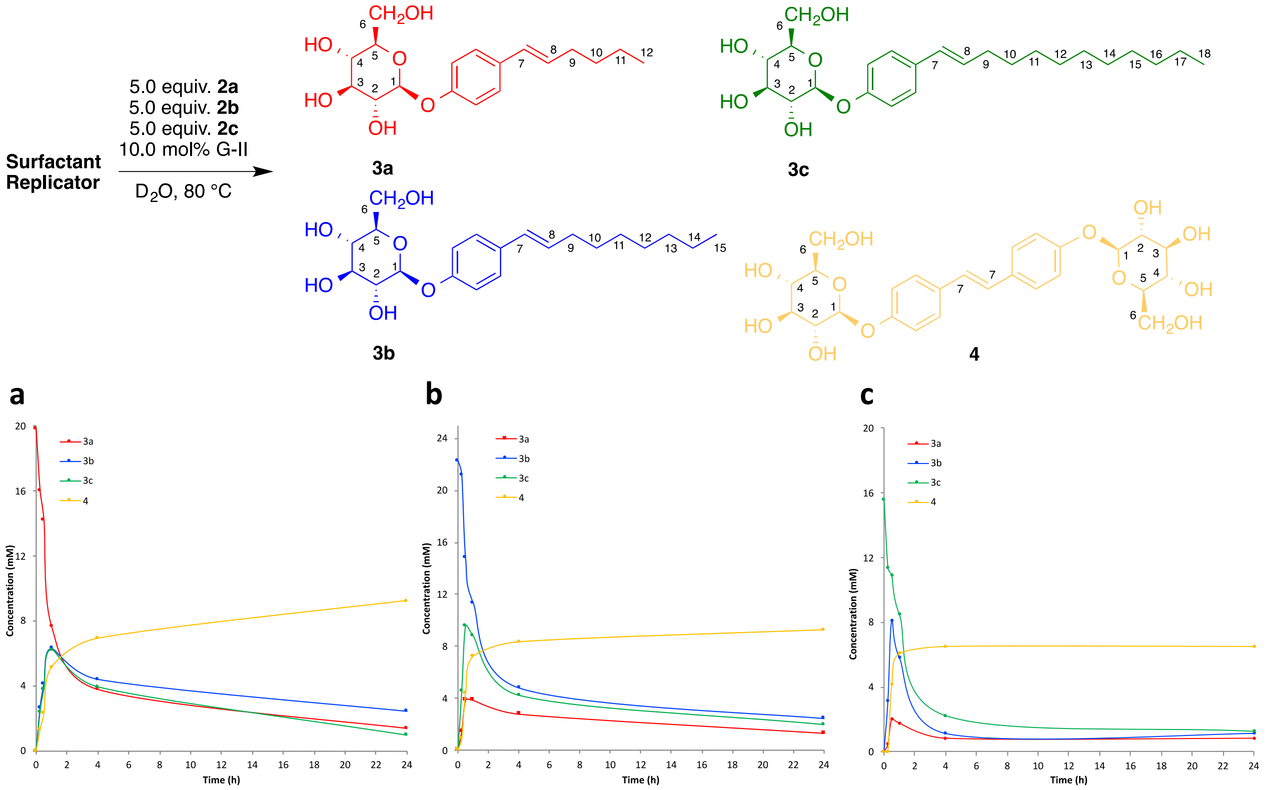

Supplementary Figure 34 Kinetic analysis of surfactant destruction. Concentration vs. time of **3a** (red), **3b** (blue), **3c** (green) and **4** (yellow), starting from: **(a)** 3a **(b)** 3b **(c)** 3c. Source data are provided as a Source Data file.

Kinetic study to determine rate constants

In a first approach to calculate rate constants for replicators **3a-c** we followed a previously reported method for autocatalytic reactions,[6] where the authors considered:

- Two mechanisms involved in the rate law: One responsible for the start of the reaction and initial formation of product and other using the product to accelerate the reaction, designated as "noncatalytic" and "catalytic" respectively. Pseudo-first-order conditions are used, and it has been assumed that "noncatalytic" mechanism is first order in one of the reactants (hydrophilic **1** in our case), as all the others being in great excess (this is fulfilled in our case, hydrophobic **2** being in excess). The "catalytic" mechanism is first order in both, limiting reactant (**1** in our case) and autocatalytic product (**3a-c** in our examples).

$$\text{Rate} = k_{\text{uncat}} \cdot [\mathbf{1}] + k_{\text{cat}} \cdot [\mathbf{1}] \cdot [\mathbf{3}] \qquad (1)$$

- Considering that one molecule of reactant consumed is converted to one molecule of product (this is fulfilled in our system, as we have shown that **4** is only formed from **3** and not directly from **1**), Supplementary Equation 1 can be written as a differential rate law:

$$-\,\mathrm{d}c/\mathrm{d}t = k_{\text{uncat}} \cdot c + k_{\text{cat}} \cdot c \cdot (c_0 - c) \qquad (2)$$

  where c represents the reactant **1** concentration and c_0 represents the reactant **1** initial concentration and $(c_0 - c)$ is the product **3a-c** concentration. In our system, this assumption is valid when the initial exponential pathway is dominant, when the destruction pathway of products **3a-c** is negligible.

- Operating, Eq. 2 can be written:

$$\text{Rate}/c = k_{\text{uncat}} + k_{\text{cat}} \cdot (c_0 - c) \qquad (3)$$

- Plotting "rate/c" against "c" linear fitting is obtained and both k_{uncat} and k_{cat} can be obtained.

- From the plot of concentration vs. time, the value of the rate at a certain time corresponds to the derivative at that time. An approximation to calculate the rate consists on consider three consecutive data points (t_1-c_1, t_2-c_2 and t_3-c_3). The rate at the middle point would be expressed as:

$$\text{Rate } (t_2\text{-}c_2) = (c_1 - c_3) / 2\Delta t \qquad (4)$$

For reactions forming **3a-c**, we report here:

a) carefully monitoring the concentration vs. time of reactant **1** (and also other species).

b) Values of concentration of **1**, calculated rate of consuming **1** and rate/[**1**] at any time.

c) Plot of rate vs. time, showing bell-shape profile.

d) Plot of rate/[**1**] vs. [**1**], with linear fitting and extracted value of rate constants.

a

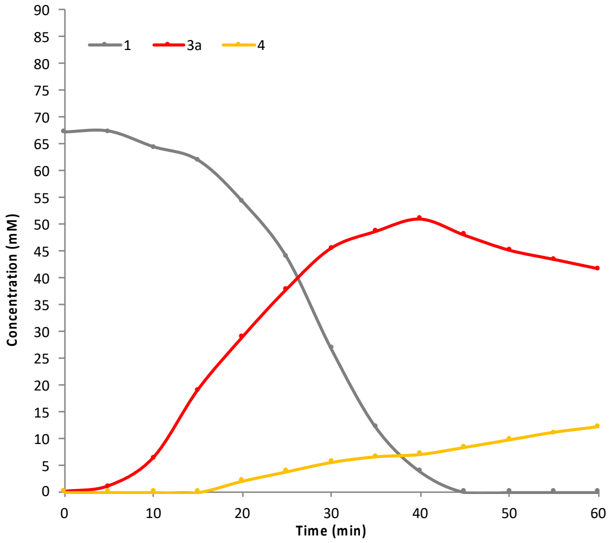

b

Time (min)	[**1**] (mM)	Rate	Rate / [**1**]
0	67.2239638	-	-
5	67.3796791	0.27419342	4.0693785
10	64.4820296	0.53641753	8.31886853
15	62.0155039	1.01455615	16.3597179
20	54.3364681	1.81283565	33.3631485
25	43.8871473	2.75165448	62.6984127
30	26.8199234	3.16962731	118.181818
35	12.1908743	2.29439544	188.20598
40	3.87596899	1.21908743	314.524556
45	0	0.3875969	-
50	0	0	-
55	0	0	
60	0	-	-

c

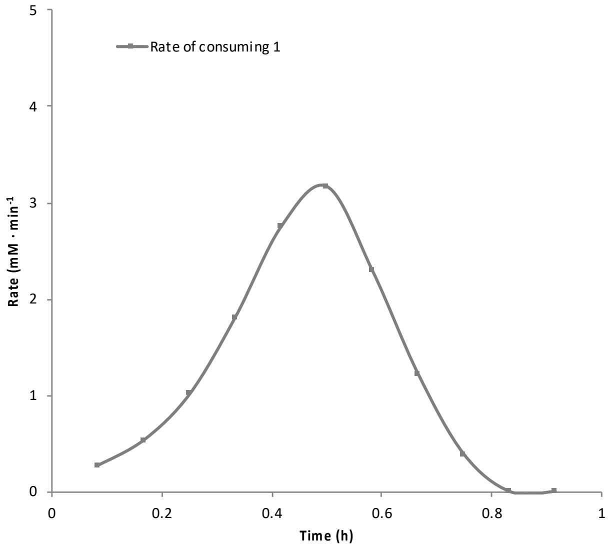

d

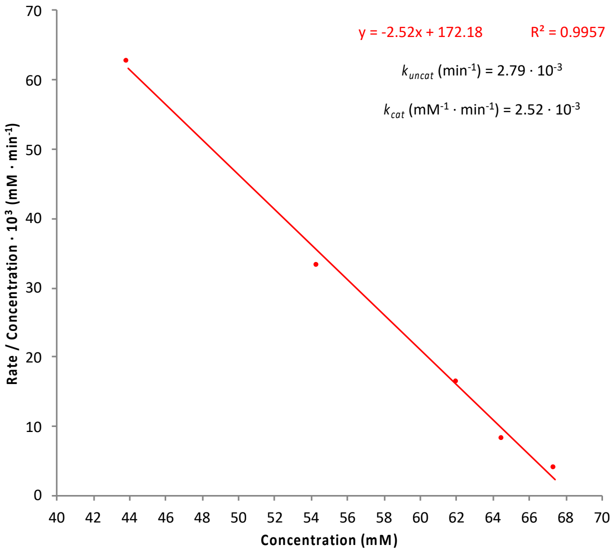

Supplementary Figure 35 Kinetic study of **3a** formation. **(a)** Kinetic analysis representing concentration vs. time of **1** (grey), **3a** (red) and **4** (yellow). **(b)** Values of concentration of reactant **1**, rate at every data point and calculation of rate/[**1**]. **(c)** Plot of rate of consuming 1 vs. time, showing characteristic bell-shape profile for autocatalytic reactions. **(d)** Plot of rate/[**1**] vs. [**1**], showing linear fitting and rate constants values. Source data are provided as a Source Data file.

a

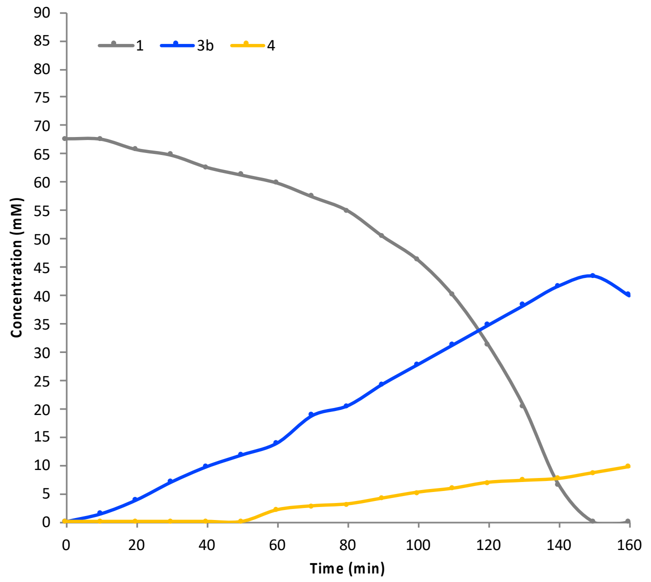

b

Time (min)	[**1**] (mM)	Rate	Rate / [**1**]
0	67.7361854	-	-
10	67.653277	0.09527322	1.40825727
20	65.830721	0.14094433	2.14101144
30	64.8343904	0.15673981	2.41754123
40	62.6959248	0.17658542	2.81653747
50	61.302682	0.13932428	2.27272727
60	59.9094392	0.19157088	3.19767442
70	57.4712644	0.24381749	4.24242424
80	55.0330895	0.34831069	6.32911392
90	50.5050505	0.43538837	8.62068966
100	46.3253222	0.52246604	11.2781955
110	40.0557297	0.74886799	18.6956522
120	31.3479624	0.97526994	31.1111111
130	20.5503309	1.23650296	60.1694915
140	6.61790317	1.02751654	155.263158
150	0	0.33089516	-
160	0	0	-

c

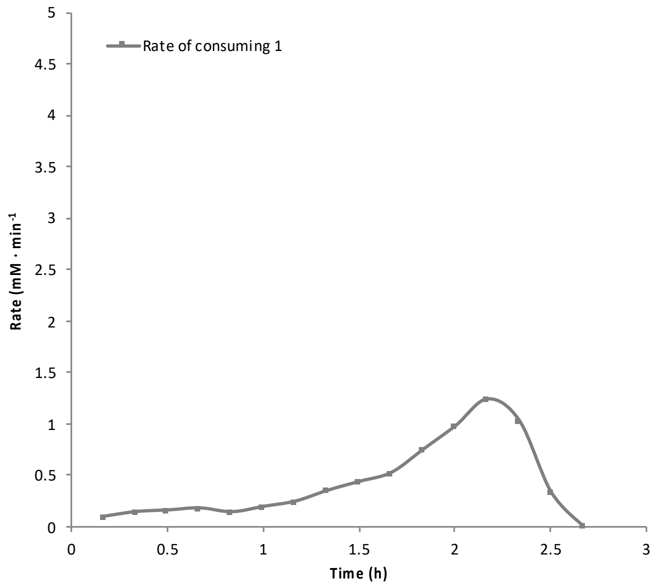

d

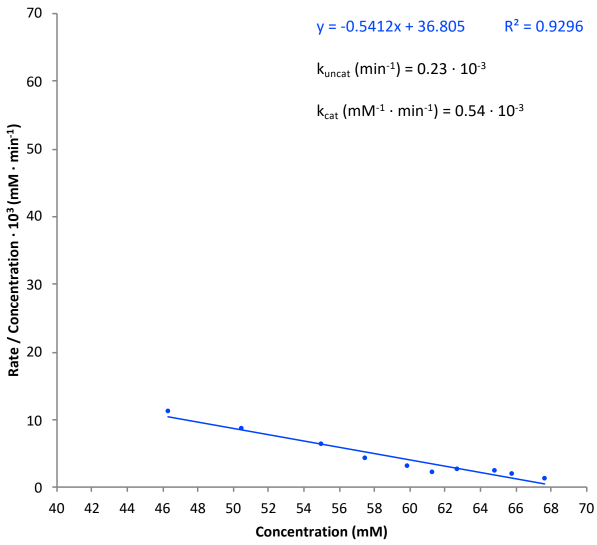

Supplementary Figure 36 Kinetic study of **3b** formation. **(a)** Kinetic analysis representing concentration vs. time of **1** (grey), **3b** (blue) and **4** (yellow). **(b)** Values of concentration of reactant **1**, rate at every data point and calculation of rate/[**1**]. **(c)** Plot of rate of consuming 1 vs. time, showing characteristic bell-shape profile for autocatalytic reactions. **(d)** Plot of rate/[**1**] vs. [**1**], showing linear fitting and rate constants values. Source data are provided as a Source Data file.

a

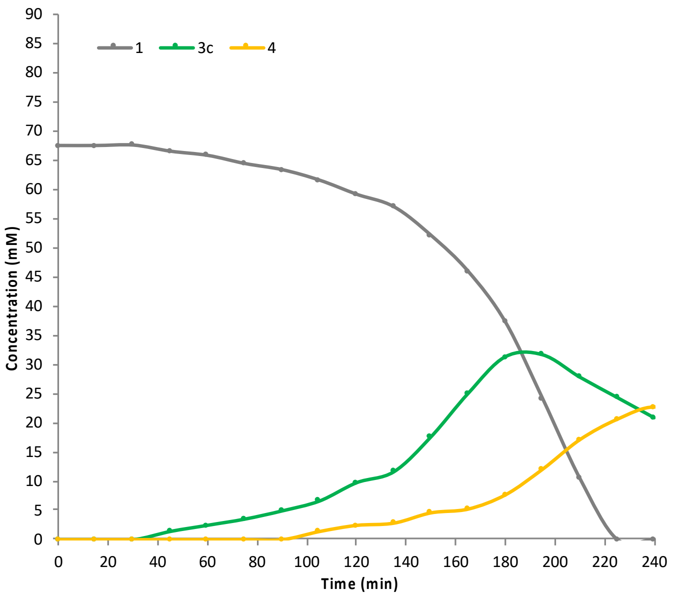

b

Time (min)	[**1**] (mM)	Rate	Rate / [**1**]
0	67.5722745	-	-
15	67.5722745	-0.0027001	-0.0399584
30	67.653277	0.032536	0.48092273
45	66.5961945	0.0587268	0.88183422
60	65.8914729	0.07047216	1.06951872
75	64.4820296	0.08221752	1.27504554
90	63.4249471	0.0943679	1.48786718
105	61.6509927	0.14040431	2.27740551
120	59.2128178	0.15228468	2.57181943
135	57.0824524	0.23544723	4.12468663
150	52.149401	0.37018136	7.09847779
165	45.9770115	0.49600523	10.7881137
180	37.2692442	0.72907761	19.5624469
195	24.1046832	0.89399745	37.0881226
210	10.4493208	0.80348944	76.8939394
225	0	0.34831069	-
240	0	0	-
255	0	-	-
260	0	-	-

c

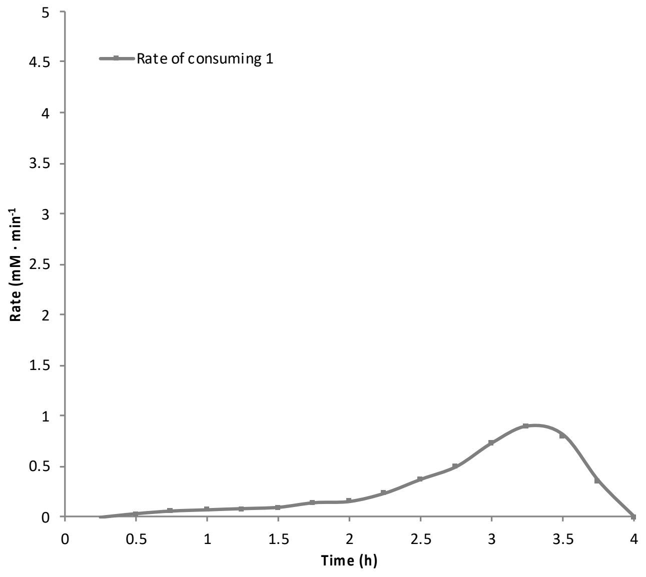

d

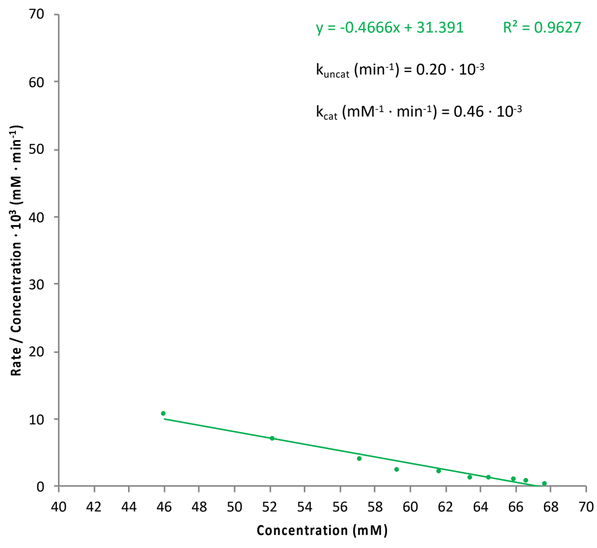

Supplementary Figure 37 Kinetic study of **3c** formation. **(a)** Kinetic analysis representing concentration vs. time of **1** (grey), **3c** (green) and **4** (yellow). **(b)** Values of concentration of reactant **1**, rate at every data point and calculation of rate/[**1**]. **(c)** Plot of rate of consuming 1 vs. time, showing characteristic bell-shape profile for autocatalytic reactions. **(d)** Plot of rate/[**1**] vs. [**1**], showing linear fitting and rate constants values. Source data are provided as a Source Data file.

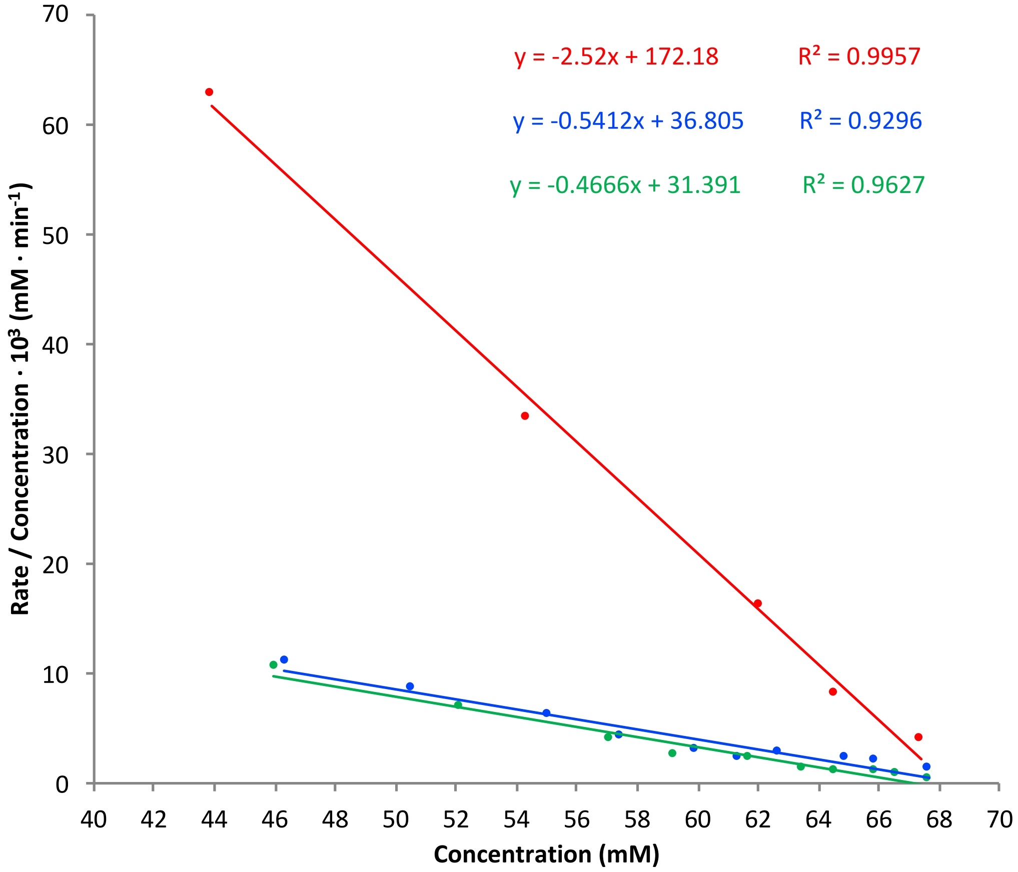

$$\text{Rate} = k_{\text{uncat}} \cdot [\mathbf{1}] + k_{\text{cat}} \cdot [\mathbf{1}] \cdot [\mathbf{3}]$$

	**2a/3a**	**2b/3b**	**2c/3c**
k_{uncat} (min^{-1})	2.79×10^{-3}	0.23×10^{-3}	0.20×10^{-3}
k_{cat} ($\text{mM}^{-1}\ \text{min}^{-1}$)	2.52×10^{-3}	0.54×10^{-3}	0.46×10^{-3}

Supplementary Figure 38 Overlapped plots of rate/[1] vs. [1] for all three replicators. Linear fitting and rate constants are displayed. Source data are provided as a Source Data file.

Critical micelle concentration (CMC) determination

An established fluorimetric method reported in the literature[7] was used for the CMC determination of surfactants. Analyses were performed using an Edinburgh Instruments Spectrofluorometer FS5 model. Instrument control and data processing were performed using Fluoracle software. Measurements were done using an equilibrated heating probe at 60 °C in quartz cuvettes with 3.0 mL of sample solution. Excitation wavelength was 358nm and emission wavelength was 430nm as reported in the literature using 1,6-diphenyl-1,3,5-hexatriene (DPH) as fluorescent molecule. From the representation of the Emission vs. Concentration the CMC can be extracted for alkenes **3a-d** (Supplementary Figures 39-42).

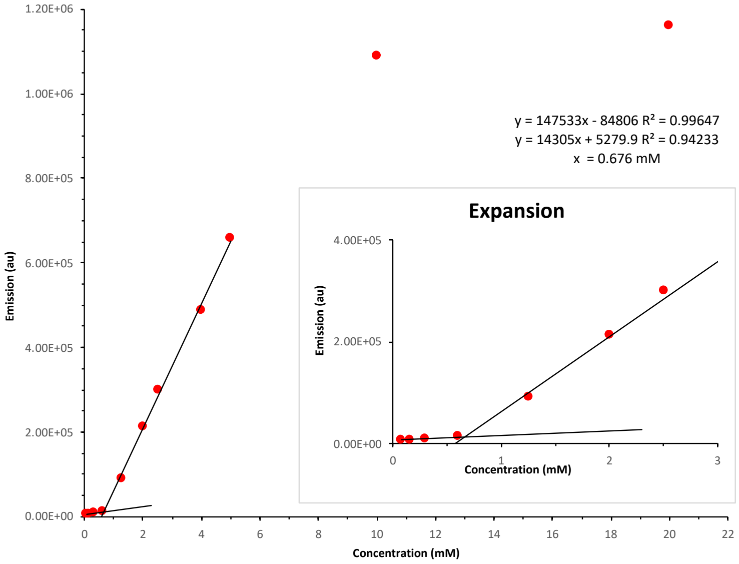

Supplementary Figure 39 Emission vs. concentration and CMC determination for **3a**. CMC of 0.676 mM was obtained. Source data are provided as a Source Data file.

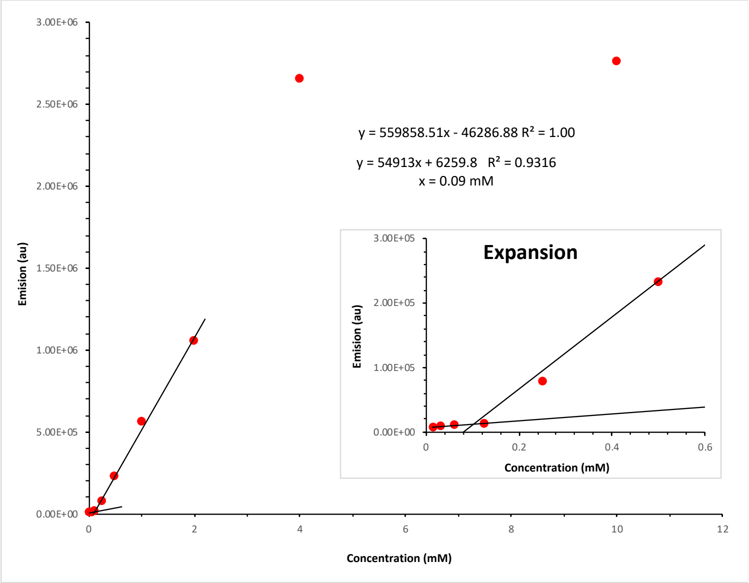

Supplementary Figure 40 Emission vs. concentration and CMC determination for **3b**. CMC of 0.09 mM was obtained. Source data are provided as a Source Data file.

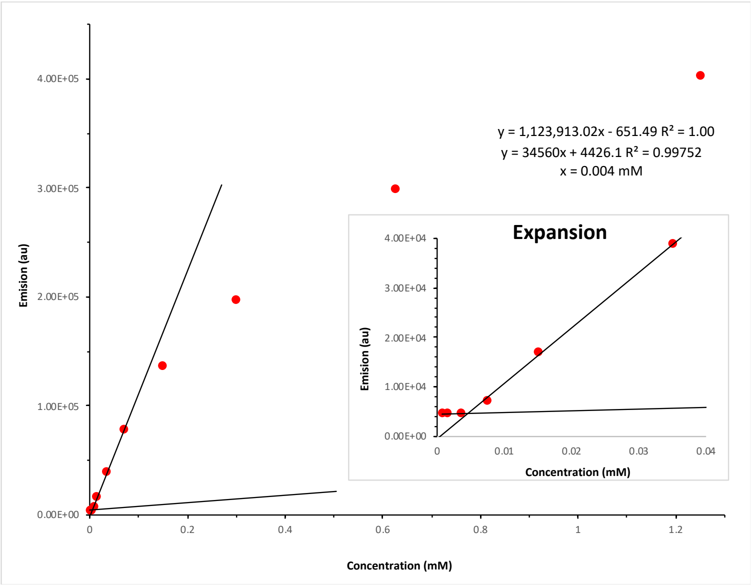

Supplementary Figure 41 Emission vs. concentration and CMC determination for **3c**. CMC of 0.004 mM was obtained. Source data are provided as a Source Data file.

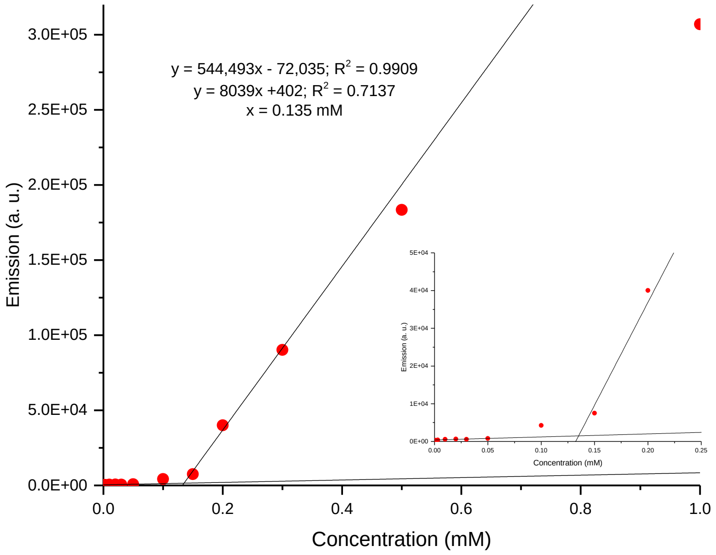

Supplementary Figure 42 Emission vs. concentration and CMC determination for **3d**. CMC of 0.135 mM was obtained. Source data are provided as a Source Data file.

Particle size measurement using DLS

Analyses were performed using a Malvern Zetasizer Nano ZEN5600 model system recording particle and molecule size. Instrument control and data processing were performed using Zetasizer software. Disposable plastic cuvettes were used with 1.0 mL of sample solution. Three repetitions of ten measurements were done for every concentration, starting from 20mM with subsequent dilutions. Measurements were done using an equilibrated heating probe at 60 °C, setting the following parameters for water:

· Dielectric constant = 66.74 · Refractive index = 1.327 · Viscosity = $0.466 \cdot 10^{-3}$ m · Pa / s

(a) For alkene **3a** a reproducible, consistent and monodisperse size of particle of 28-30nm is detected from 7.5mM to 0.675mM (Supplementary Figure 43).

a

b

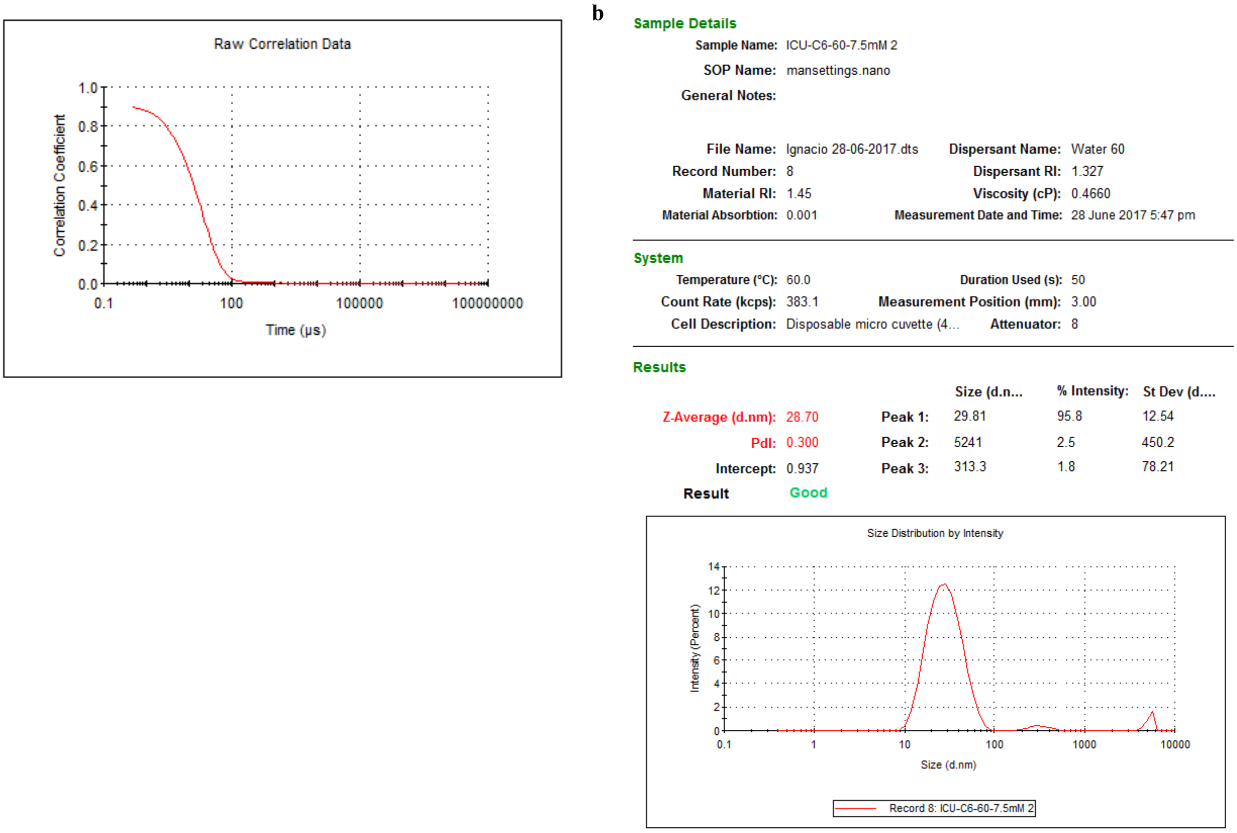

Supplementary Figure 43 DLS experiment using **3a.** Alkene **3a** at 7.5mM **(a)** correlation data. **(b)** Size distribution.

(b) For amphiphile **3b**, at high concentrations, from 10.0mM to 0.3mM, large particles of 200-240nm were observed (Supplementary Figure 44). At lower concentrations, below 0.3mM, polydispersion started being detected.

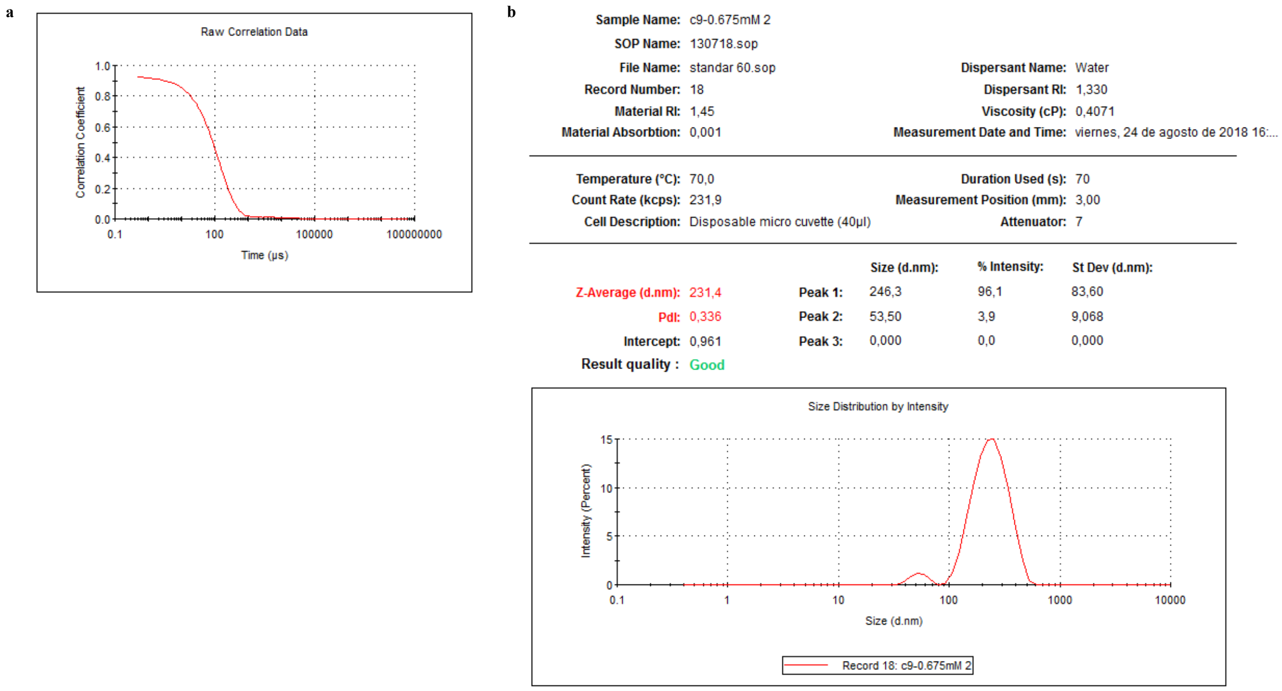

Supplementary Figure 44 DLS experiment using **3b**. Alkene **3b**, at 0.675mM, **(a)** correlation data. **(b)** Size distribution.

(c) For amphiphile **3c**, at high concentrations, from 10.0mM to 0.3mM, large particles of 300-340nm were revealed (Supplementary Figure 45). At lower concentrations, below 0.3mM, particles with an average diameter of 30nm could be detected.

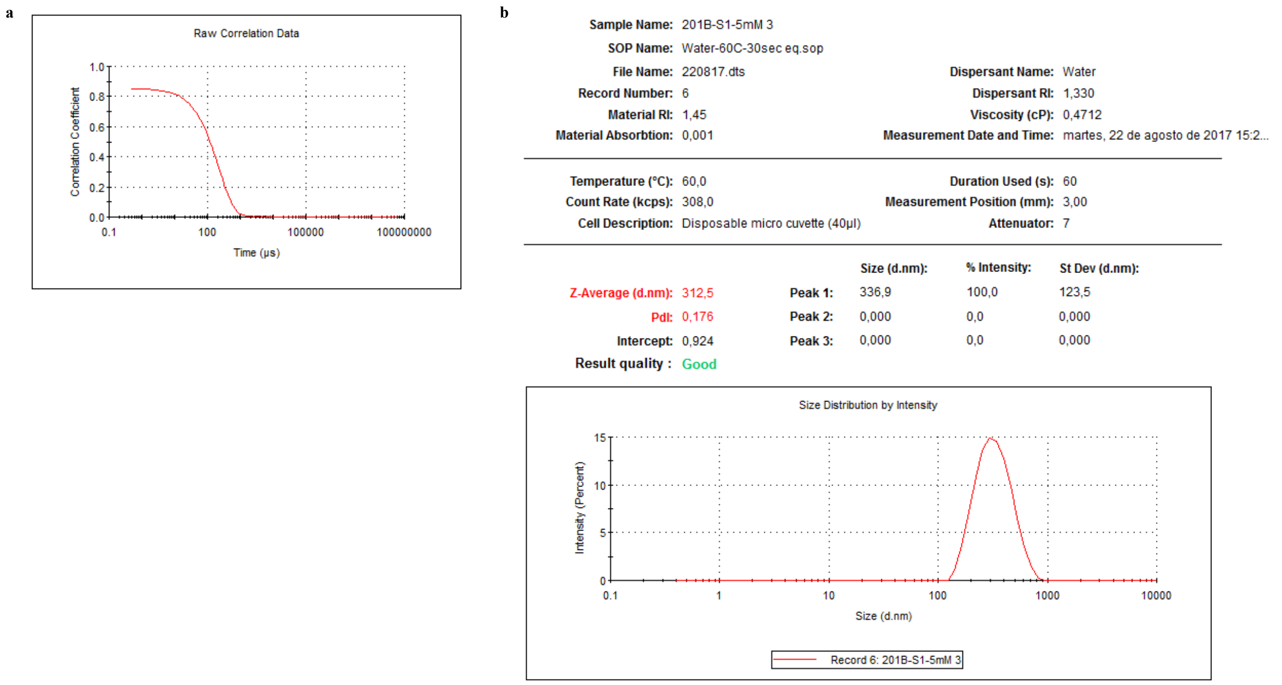

Supplementary Figure 45 DLS experiments using **3c**. Alkene **3c,** at 5 mM **(a)** correlation data. **(b)** Size distribution.

(d) Using a 1:1:1 mixture of amphiphiles **3a:3b:3c**, at concentrations ranging from 10.0mM to 0.3mM, large particles of 200nm were observed (Supplementary Figure 46). At lower concentrations, below 0.3mM, polydispersion is increasingly observed.

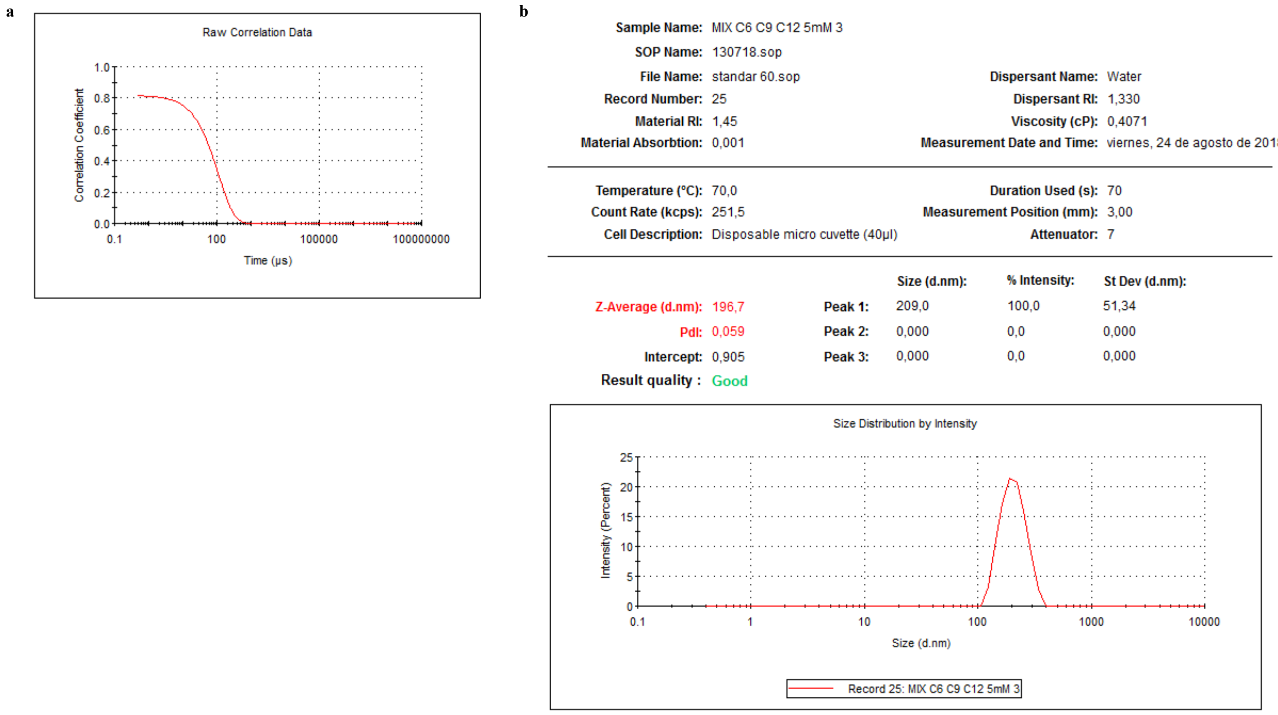

Supplementary Figure 46 DLS experiments using a mixture of alkenes **3a-c**. A mixture of alkenes **3a:3b:3c** in 1:1:1 molar ration and 5 mM concentration in every alkene. **(a)** correlation data **(b)** distribution size.

e) For amphiphile **3d**, particle size of 90-110 nm was consistently observed at concentrations between 0.25 mM and 2 mM (Supplementary Figure 47).

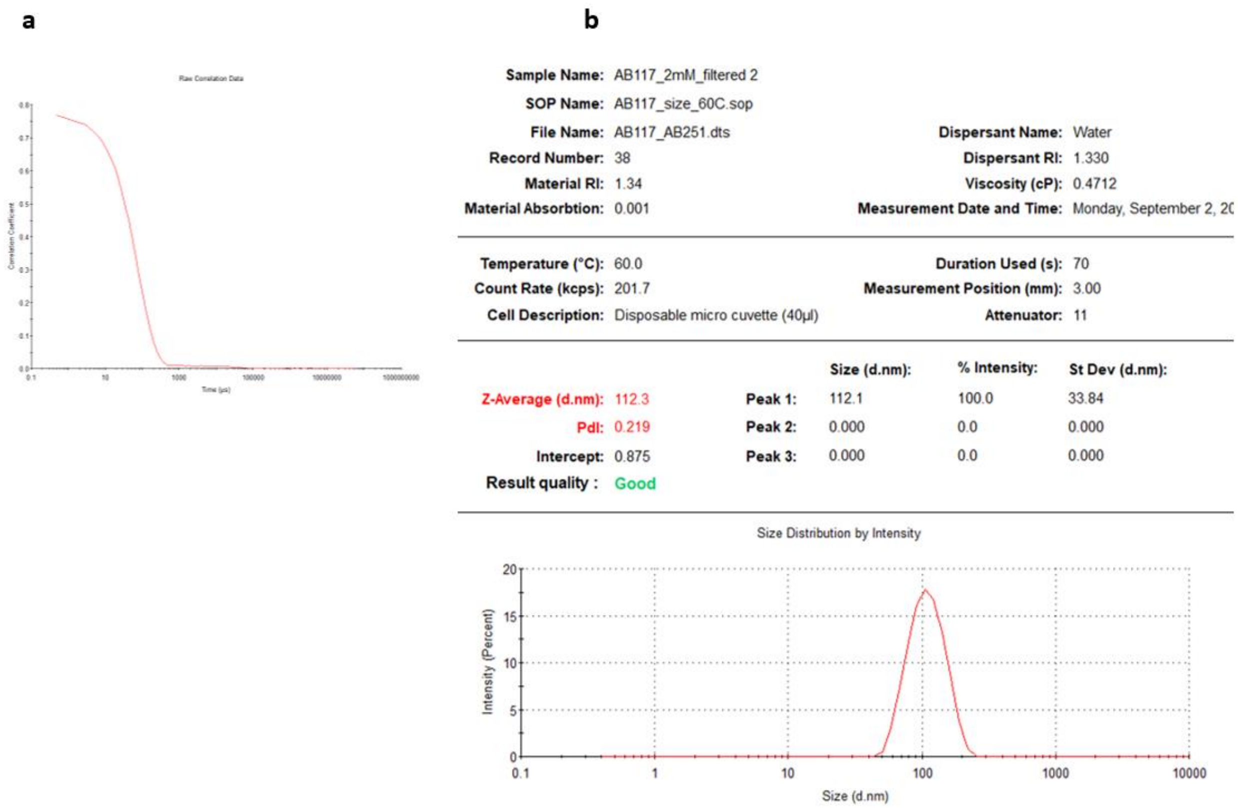

Supplementary Figure 47 DLS experiments using **3d**. Alkene **3d,** at 2 mM **(a)** correlation data. **(b)** Size distribution.

TEM experiments

TEM images were produced using negative staining. 10 µl of sample was applied to freshly glow discharged carbon Formvar 200 mesh copper grids for 2 mins, blotted with filter paper and stained with 2% uranyl acetate for 10 s, then blotted and air dried. Grids were imaged in a FEI Tecnai 12 TEM at 120 kV using a Gatan OneView CMOS camera.

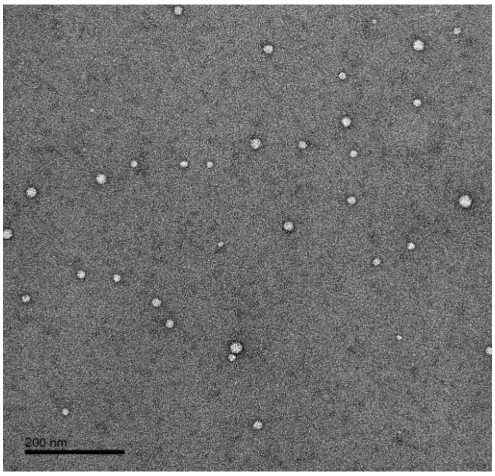

Supplementary Figure 48 TEM images for amphiphile **3a**. Showing particles with a diameter average size of 27nm for amphiphile **3a** in a 2.0 mM solution. Including a scale bar of 200nm.

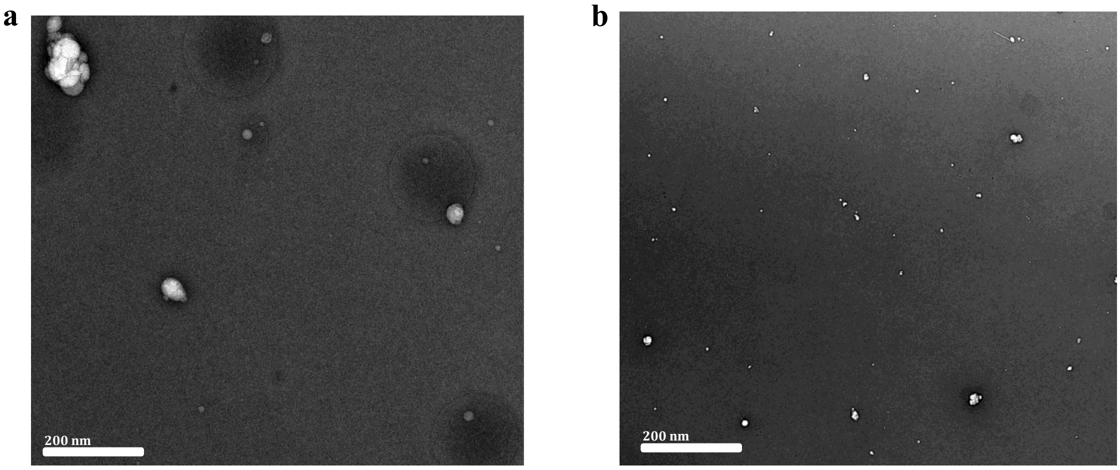

Supplementary Figure 49 TEM images for amphiphile **3b. (a)** At a concentration of 2.0 mM, aggregates of a 120nm size are observed with a scale bar of 200nm. **(b)** At lower concentration, 0.3mM, smaller particles with a diameter average size of 28nm are detected with a scale bar of 200nm.

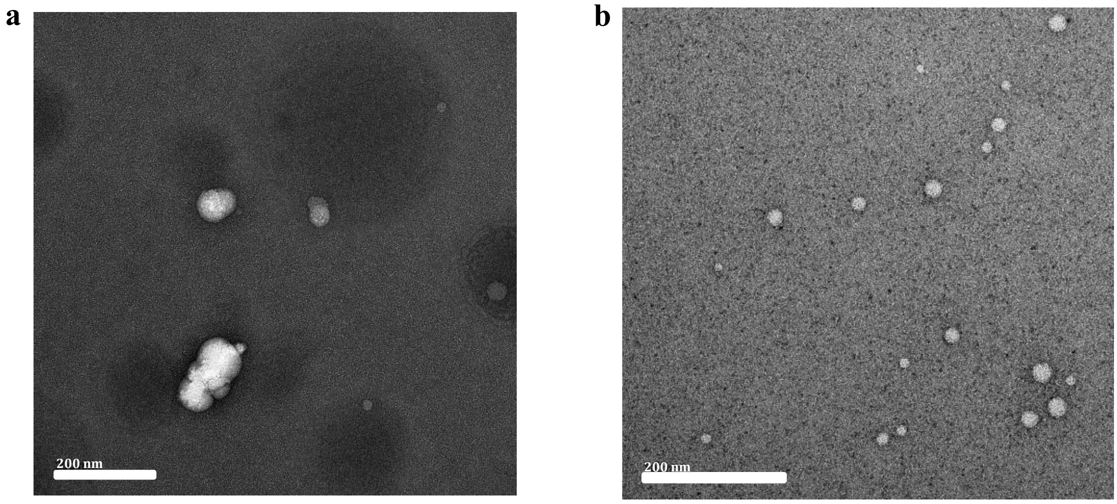

Supplementary Figure 50 TEM images for amphiphile **3c. (a)** At a concentration of 2.0 mM, aggregates of a 190nm size are observed with a scale bar of 200nm. **(b)** At lower concentration, 0.3mM, smaller particles with a diameter average size of 30nm are detected with a scale bar of 200nm.

Supplementary References

1. Jiang, A. J., Zhao, Y., Schrock, R. R. & Hoveyda, A. H. Highly Z-selective metathesis homocoupling of terminal olefins. *J. Am. Chem. Soc.* **131**, 16630–16631 (2009).

2. Twidle, A. M. *et al.* Identification of in situ flower volatiles from kiwifruit (Actinidia chinensis var. deliciosa) cultivars and their male pollenisers in a New Zealand orchard. *Phytochemistry* **141**, 61–69 (2017).

3. Czaban, J., Schertzer, B. M. & Grela, K. Low catalyst loadings in self-metathesis of 1-dodecene. *Adv. Synth. Catal.* **355**, 1997–2006 (2013).

4. Oulmi, D., Maillard, P., Guerquin-Kern, J. L., Huel, C. & Momenteau, M. Glycoconjugated Porphyrins. 3. Synthesis of Flat Amphiphilic Mixed meso- (Glycosylated aryDarylporphyrins and Mixed meso- (Glycosylated aryl)alkylporphyrins Bearing Some Mono- and Disaccharide Groups. *J. Org. Chem.* **60**, 1554–1564 (1995).

5. Colomer, I., Morrow, S. M. & Fletcher, S. P. A transient self-assembling self-replicator. *Nat. Commun.* **9**, 2239 (2018).

6. Mata-Perez, F. & Perez-Benito, J. F. The kinetic rate law for autocatalytic reactions. *J. Chem. Educ.* **64**, 925 (2009).

7. Chattopadhyay, A. & London, E. Fluorimetric determination of critical micelle concentration avoiding interference from detergent charge. *Anal. Biochem.* **139**, 408–412 (1984).
